# Comprehensive genetic profiling of sensorineural hearing loss using an integrative diagnostic approach

**DOI:** 10.1016/j.xcrm.2025.102206

**Published:** 2025-06-30

**Authors:** Sang-Yeon Lee, Seungbok Lee, Seongyeol Park, Sung Ho Jung, Yejin Yun, Won Hoon Choi, Ju Hyuen Cha, Hongseok Yun, Sangmoon Lee, Myung-Whan Suh, Moo Kyun Park, Jae-Jin Song, Byung Yoon Choi, Jun Ho Lee, Tong Mook Kang, Young Seok Ju, June-Young Koh, Jong-Hee Chae

**Affiliations:** 1 Department of Otorhinolaryngology, Seoul National University College of Medicine, Seoul National University Hospital, Seoul, South Korea; 2 Department of Genomic Medicine, Seoul National University Hospital, Seoul, South Korea; 3 Sensory Organ Research Institute, Seoul National University Medical Research Center, Seoul, South Korea; 4 Department of Pediatrics, Seoul National University College of Medicine, Seoul National University Children’s Hospital, Seoul, South Korea; 5 Inocras, Inc., San Diego, CA, USA; 6 Department of Otorhinolaryngology, Seoul National University College of Medicine, Seoul National University Bundang Hospital, Seongnam, South Korea; 7 Department of Physiology, Sungkyunkwan University School of Medicine, Samsung Medical Center, Suwon, South Korea; 8 Graduate School of Medical Science and Engineering, Korea Advanced Institute of Science and Technology, Daejeon, South Korea

**Keywords:** sensorineural hearing loss, whole-genome sequencing, stepwise genomic approach, genotype-phenotype correlations, rare genetic disorder, precision medicine, molecular diagnostics

## Abstract

Despite the advent of next-generation sequencing, diagnosing genetic disorders remains challenging. We perform comprehensive genomic profiling of 394 families (752 individuals) with sensorineural hearing loss (SNHL) using a systematic multi-tiered approach, from single-gene analysis to whole-genome sequencing (WGS), complemented by functional assays and bioinformatic analysis. Our strategy achieves a cumulative diagnostic yield of 55.6% (219 families), with automated WGS analysis identifying pathogenic variants in an additional 20 families, primarily structural variants. Comparative analysis reveals higher frequencies of single pathogenic alleles in recessive genes within our cohort compared to controls. Subsequent analysis, including *in silico* predictions and *in vitro* validation, identifies three deep intronic pathogenic variants on opposite alleles. These findings demonstrate the value of comprehensive genomic analysis in resolving undiagnosed cases. Finally, we map the genome-phenome landscape of SNHL at the level of inner ear function. Our results highlight WGS as a transformative tool for precision medicine in genetic diseases.

## Highlights

• A multi-tiered approach resolves 55.6% of SNHL cases in 394 families
• WGS-based diagnostics show clinical utility in real-world SNHL settings
• Functional categorization reveals the genome-phenome landscape of SNHL
• Integrative diagnostic approaches will advance precision medicine for SNHL

## Graphical abstract

**Figure:**
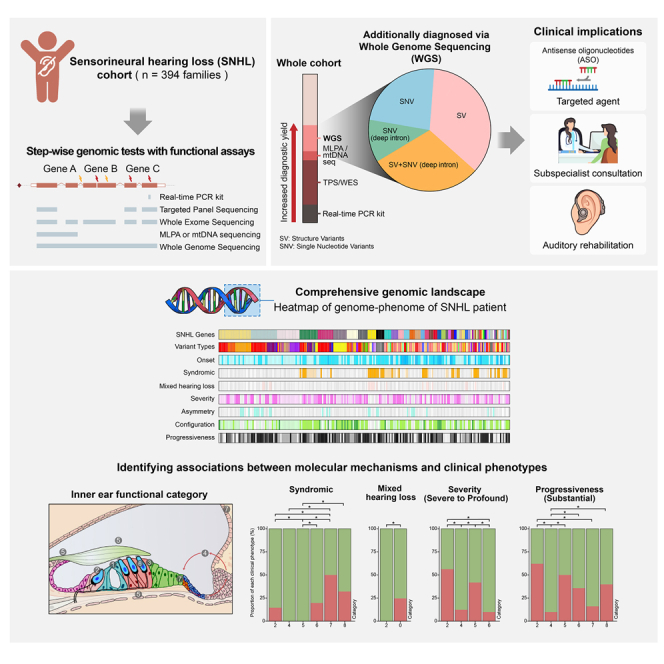

## Teaser

We performed comprehensive genomic profiling of 394 families with SNHL using a stepwise diagnostic approach. Whole-genome analysis and functional assays enabled diagnosis in challenging cases, including those with structural or intronic variants. These findings underscore the clinical value of an integrative approach in diagnosing SNHL and uncovering genotype-phenotype relationships.

## Introduction

Hearing is the primary sense used for human communication and an important component in the development of language and music. Thus, hearing impairment, the most common sensory deficit in humans, is a major public health problem, affecting approximately 466 million people worldwide (World Health Organization, https://who.int/news-room/fact-sheets/detail/deafness-and-hearing-loss). Sensorineural hearing loss (SNHL)—i.e., defective sound signaling in the auditory sensory system—can be caused by multiple etiologies, including genetic causes, congenital infections, trauma, ototoxic medications, and autoimmune disorders.^1^ Since the 2010s, advances in high-throughput next-generation sequencing (NGS) technologies have facilitated extensive elucidation of the genetic backgrounds of SNHL, with a focus on monogenic forms of deafness. Notably, mouse genetics studies have helped reveal the physiological basis of SNHL in humans and the associated molecular functions.^2^

Despite growing recognition of the significance of genetic diagnosis of SNHL, it remains challenging to identify a genetic diagnosis in SNHL with substantial genetic heterogeneity.^3^^,^^4^ NGS is increasingly favored for genetic diagnosis due to its capacity for simultaneous large-scale genetic loci screening, and methods like targeted panel sequencing (TPS) and whole-exome sequencing (WES) are widely used in real-world practice.^5^^,^^6^^,^^7^ In the literature, targeted sequencing for SNHL has achieved diagnostic yields of between 12.7% and 64.3%.^8^^,^^9^^,^^10^^,^^11^ However, even after exome sequencing, approximately 50% of cases remain genetically elusive.

As the cost of sequencing dramatically declines,^12^ the clinical application of whole-genome sequencing (WGS), which has a higher capability to detect a more diverse spectrum of genomic variants that had not previously been captured by exome sequencing or other targeted approaches,^13^^,^^14^ becomes more feasible. Recent studies have shown the clinical utility of WGS for the genetic diagnosis of several disorders and effectively shortening their diagnostic odyssey, which is increasingly considered as a first-line genetic test.^15^^,^^16^^,^^17^^,^^18^^,^^19^ However, WGS is not yet widely applied in routine clinical settings for diagnosing patients with rare diseases, including SNHL, due to several limitations, such as the difficulties of rapid bioinformatic analysis and accurate clinical interpretation. Additionally, although whole genomes are sequenced, the analysis is often limited to *in silico* gene panels or the coding regions of the genome.^20^

In the present study, we comprehensively explored the genetic landscape of 394 prospective SNHL families. Using a stepwise approach from single target gene analysis to WGS, we evaluated the additional diagnostic value of WGS. We implemented an automated WGS bioinformatics pipeline, integrating in-house algorithms with manual curation by both otologists and medical geneticists. This approach allowed a comprehensive analysis of all variant types and improved the diagnostic yield for previously undiagnosed patients. Further analysis of deep intronic regions identified novel pathogenic variants. These findings refined the genotype-phenotype landscape of SNHL, uncovering gene groups related to inner ear molecular functions that correlate with phenotypes. Our results demonstrate the clinical utility of an integrated molecular diagnostic approach, including WGS, in real-world SNHL practice, paving the way toward precision medicine.

## Results

### A stepwise approach of genetic testing for patients with SNHL

**Figure 1:**
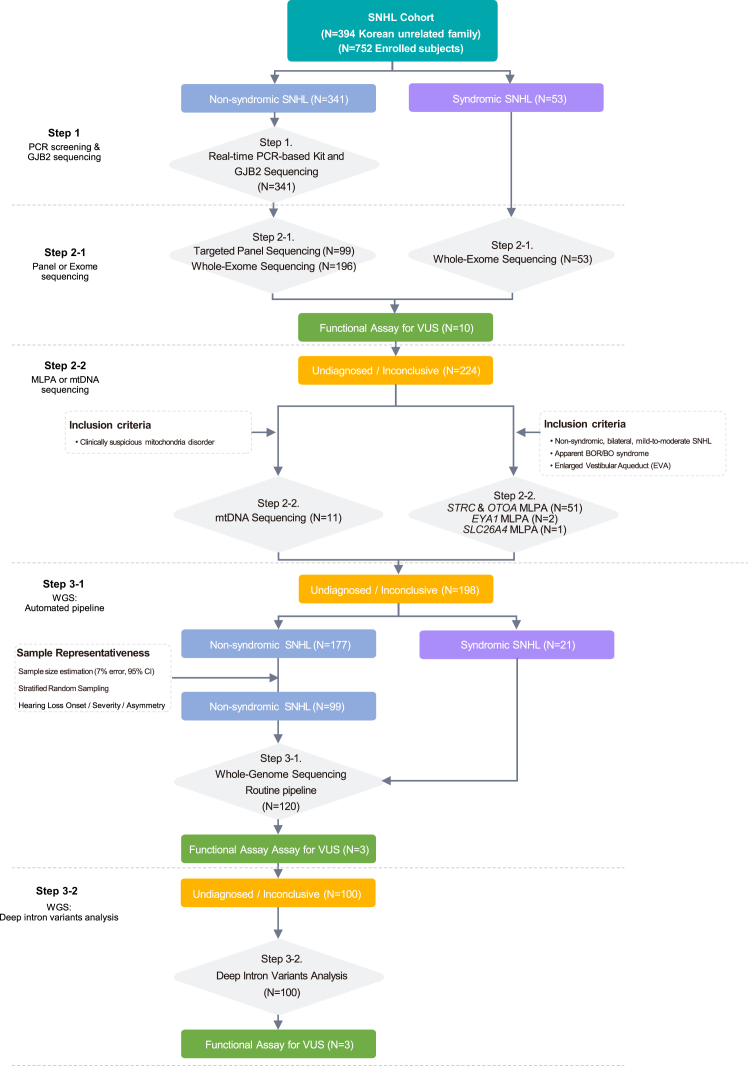
Study design and diagnostic pipeline Flow diagram illustrating a prospective, step-by-step genetic approach of 394 unrelated SNHL families and 752 individuals, including probands, in our cohort study.

We conducted a stepwise approach of genetic testing, including PCR-based screening, Sanger sequencing, TPS, WES, mitochondrial DNA (mtDNA) sequencing, multiplex ligation-dependent probe amplification (MLPA), and WGS, in a prospectively recruited SNHL cohort (*n* of probands = 394; *n* of participants including probands and their family members = 752; Table S1), including non-syndromic SNHL (ns-SNHL; *n* = 341, 86.5%) and syndromic SNHL (s-SNHL; *n* = 53, 13.5%). Genetic testing was structured into five sequential steps (Figures 1 and S1A). In step 1, we performed PCR screening for 22 variants from 10 classical deafness genes (*GJB2*, *SLC26A4*, *TMPRSS3*, *CDH23*, *OTOF*, *TMC1*, *ATP1A3*, *MPZL2*, *COCH*, and 12S rRNA) and *GJB2* single-gene sequencing (Figure S2).^21^^,^^22^ Among the members of the cohort, patients with ns-SNHL (*n* = 341) were initially subjected to step 1, and the patients with ns-SNHL who remained undiagnosed after step 1 (*n* = 295) were then subjected to the next steps (steps 2-1 and 2-2). In contrast, patients with s-SNHL (*n* = 53) underwent step 2-1 as their initial test. In step 2-1, 99 and 249 patients were subjected to TPS (including 246 hearing loss-related genes, Figure S3A) and WES, respectively. After that, undiagnosed patients with suggestive clinical features were subjected to step 2-2. In this step, patients with bilateral, symmetric, mild-to-moderate ns-SNHL were tested using *STRC/OTOA* MLPA.^23^ Undiagnosed patients with apparent branchio-oto-renal/branchio-otic (BOR/BO) syndrome (*n* = 2) underwent *EYA1* MLPA.^24^ One undiagnosed patient with radiological evidence of enlarged vestibular aqueducts (EVAs) underwent *SLC26A4* MLPA. Additionally, 11 undiagnosed patients with suspected mitochondrial phenotypes underwent mtDNA panel sequencing. In step 3-1, among patients who remained undiagnosed, all patients with s-SNHL and a representative subset of patients with ns-SNHL were selected for WGS using a well-thought-out sample size estimation with a stratified sampling approach (Figure S1B). For the remaining undiagnosed patients (*n* = 100), deep intronic regions of SNHL-related genes were screened to identify additional pathogenic variants in step 3-2. After stepwise genetic testing, patients with suspected pathogenic genetic causes underwent subsequent bioinformatic analyses and curation. Multidisciplinary molecular board meetings, comprising both clinicians and genome scientists, were then conducted to confirm genetic diagnosis.

### Incremental improvement in the diagnostic yield through stepwise genetic testing for SNHL

**Figure 2:**
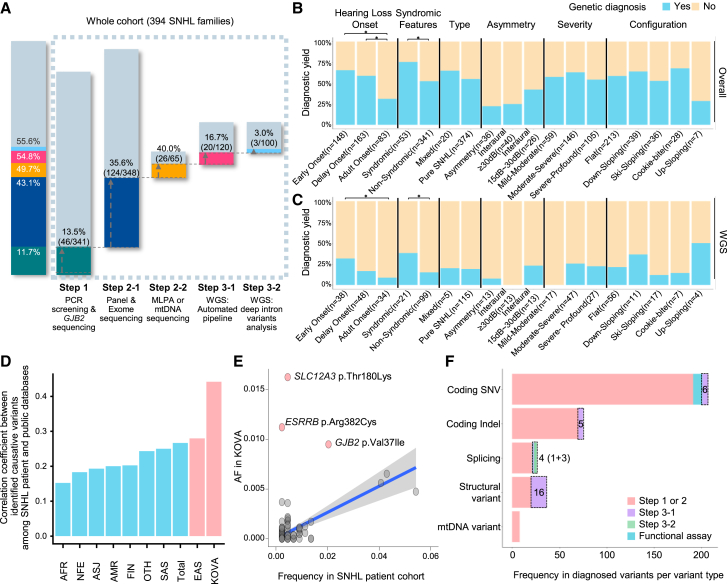
Stepwise genetic diagnosis outcomes in patients with SNHL (A) Diagnostic yield of each genetic test for the whole SNHL cohort. Bar graph showing the cumulative diagnostic rate according to genetic diagnosis steps. (B and C) Diagnostic yield based on SNHL phenotypes and comparative analysis within the whole SNHL cohort (*n* = 394 families) (B) and within the WGS cases (*n* = 120 families) (C). Statistical significance for hearing loss onset and syndromic features was determined using one-way ANOVA with Bonferroni’s multiple comparisons tests and the t test, respectively. Significance levels are indicated as ∗*p* < 0.05. (D) Pearson’s correlation coefficient values for causative variants between the allele frequencies (AFs) in our cohort and those from other populations. (E) Dot plot shows the AF correlation between our cohort and KOVA. There are three variants (pink dots) showing higher AFs in KOVA. A fitted line of linear regression model (blue line) and 95% confidence intervals (gray area) are displayed. (F) Distribution of variant subtypes identified at each diagnostic step. WGS, whole-genome sequencing; MLPA, multiplex ligation-dependent probe amplification; mtDNA, mitochondrial DNA; SNHL, sensorineural hearing loss; AFR, African American; NFE, non-Finnish European; ASJ, Ashkenazi Jewish; AMR, admixed Americans or Latino; FIN, Finnish European; OTH, other ethnic origin; SAS, South Asian; EAS, East Asian; SNV, single-nucleotide variant; indel, insertion/deletion.

Through comprehensive genetic testing, we incrementally improved the diagnostic yield in the cohort, identifying disease-causing variants in 219 families (55.6%; Figure 2A). Following the stepwise approach, in step 1, we identified causal variants in 46 out of 341 patients (diagnostic yield for step 1: 13.5%; cumulative yield: 11.7%). Specifically, for *GJB2* diagnostics, the PCR-based screening kit alone produced a 3.81% yield (13/341), whereas the combined PCR-based screening kit and *GJB2* single-gene sequencing resulted in a 6.75% yield (23/341) in patients with ns-SNHL (Figure S2C). In step 2-1, causal variants were found in 124 out of 348 patients (diagnostic yield for step 2-1: 35.6%; cumulative yield: 43.1%). Among patients with ns-SNHL, the diagnostic yields of TPS and WES were 30.3% (30/99) and 34.7% (68/196), respectively (Figure S3B). The distribution of diagnostic genes between TPS and WES was largely consistent, except for two variants that were detected only by WES: a heterozygous *ANKRD11* variant from SNUH 748 and from homozygous *SLC12A3* variants in SNUH 971 (Figures S3C and S3D). In step 2-2, 26 out of 65 patients received a confirmed molecular diagnosis (yield for step 2-2: 40%; cumulative yield: 49.7%). WGS was subsequently performed on 120 patients, identifying additional causal variants in 23 probands (20 in step 3-1 and 3 in step 3-2; yield for step 3: 19.2%; cumulative yield: 55.6%). Details of the individual patients, including the tests performed, diagnostic outcomes, and identified variants, are summarized in Table S2.

Multivariate analysis revealed that the rate of genetic diagnosis was higher among patients with early identification of SNHL (adjusted odds ratio [OR], 1.32; 95% confidence interval [CI], 1.11–1.57) and those with a family history (adjusted OR, 1.55; 95% CI, 1.31–1.82; Figure 2B; Table S3). Similarly, patients with syndromic features were more likely to have identified genetic variants (adjusted OR, 1.44; 95% CI, 1.17–1.70). In contrast, a lower rate of genetic diagnosis was observed in patients with adult-onset SNHL (adjusted OR, 0.50; 95% CI, 0.35–0.68), asymmetric hearing loss (adjusted OR, 0.38; 95% CI, 0.18–0.64), and interaural asymmetry (adjusted OR, 0.43; 95% CI, 0.23–0.68). Additionally, in the context of WGS, several features were associated with a higher likelihood of achieving a genetic diagnosis (Figure 2C; Table S4). The multivariate analyses suggested that genetic diagnosis was significantly related to the presence of syndromic features (adjusted OR, 2.51; 95% CI, 1.15–5.04) and early identification through a failed newborn hearing screening (NHS) (adjusted OR, 2.35; 95% CI, 1.13–4.97). In addition, the diagnostic yield significantly varied depending on the WGS approach. Trio-based WGS had a higher likelihood of identifying causal variants (adjusted OR, 3.71; 95% CI, 1.67–9.68), whereas singleton WGS was less effective (adjusted OR, 0.32; 95% CI, 0.12–0.71).

Further, we conducted the correlation analysis between the frequencies of causative variants among our cohort and population allele frequencies of the variants from public databases, such as the gnomAD^25^ and KOVA^26^ (Korean Variant Archive: Korean population database; Figures 2D and S4A). We observed the strongest association with East Asians compared to other ethnic groups within gnomAD and a notably higher association with KOVA, suggesting that patient ethnicity should be considered in variant discovery. We discovered three outlier variants (*SLC12A3* p.Thr180Lys, *ESRRB* p.Arg382Cys, and *GJB2* p.Val37Ile) when comparing the variant frequencies with allele frequencies in KOVA (Figures 2E; Table S5). These variants have higher frequencies in the Korean population than in diagnosed patients, suggesting their low penetrance features and heterogeneous phenotypic manifestations.^27^ Despite their relatively high frequencies in the population, these variants could contribute to their disease-causing potential through pathogenic mechanisms that affect protein function or gene regulation. Functional assays of the *ESRRB* p.Arg382Cys variant revealed that it disrupts protein stability, reduces transcriptional activity, and alters the expression of downstream target genes essential for hearing function.^27^ In addition, the *GJB2* p.Val37Ile variant, classified as a hypomorph allele, results in a milder phenotype compared to other pathogenic variants. Notably, this variant is recognized as pathogenic but exhibits variable expressivity and incomplete penetrance.^28^ Likewise, although *SLC12A3* p.Thr180Lys demonstrates functional pathogenicity in the context of Gitelman syndrome or ns-SNHL mimics, its high allele frequency may be explained by variable expressivity, incomplete penetrance, or late-onset progressive nature.^29^

In conclusion, from step 1 to step 3-2, we identified the genetic causes of SNHL in 219 out of 394 probands (55.6%), with 23 diagnoses made through WGS. Among the identified variants, deep intronic variants (3 out of 3 variants) and structural variants (SVs) (16 out of 36 variants) were predominantly detected through WGS, and 13 variants of uncertain significance (VUSs) and 3 SVs required functional assays to validate their pathogenicity (Figures 2F and S4B).

### Comprehensive characterization of causative variants in SNHL

**Figure 3:**
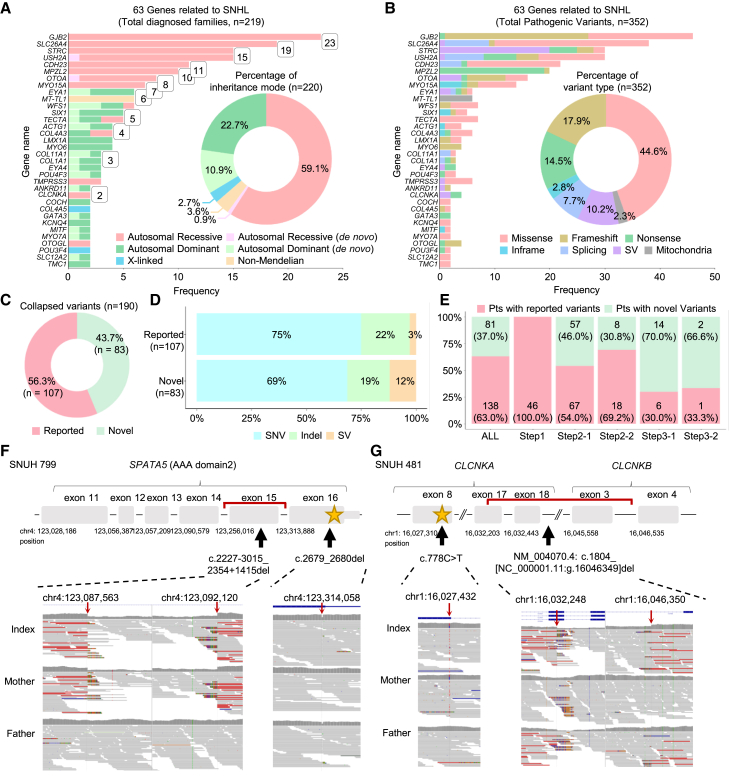
Genomic landscape of the SNHL cohort (A) Bar plot showing the frequencies and inheritance patterns of 63 SNHL-associated genes from 219 genetically diagnosed families. Pie chart showing the percentages of inheritance patterns. (B) Bar plot showing the mutational landscape of the total 352 likely pathogenic or pathogenic variants among the 63 SNHL genes. Pie chart showing the percentages of variant types. (C) Proportion of novel variants among identified causal variants. (D) Structural variants (SVs) were more common among novel variants compared to previously reported variants. (E) Novel variants were frequently identified through WGS (step 3-1) and SpliceAI-based deep intronic variant analysis (step 3-2). (F and G) Schematic illustrations showing the pathogenic variant that deletes exons in *SPATA5* and *CLCNKA* in the probands (top, respectively). The genomic regions corresponding to each variant are visualized using the Integrative Genomics Viewer (bottom) for each figure. The detailed reconstruction of the *CLCNKA* deletion, including nearby copy-number variations, is illustrated in Figure S10. SNHL, sensorineural hearing loss; SNV, single-nucleotide variant; indel, insertion/deletion; SV, structural variation.

Using the genetic findings obtained through comprehensive stepwise genetic tests, we illustrated a mutational landscape of SNHL. Collectively, 63 genes were identified as disease causing in 219 genetically diagnosed families (Figures 3A and S5A). *GJB2* was the most frequently affected gene (10.5%, 23/219), followed by five genes (*SLC26A4*, *STRC*, *USH2A*, *CDH23*, and *MPZL2*) that were found in at least ten unrelated families (collectively >40% of all diagnosed cases). Conversely, 29 SNHL-associated genes were detected from only one family (collectively 13.2%; 29/219; Figure S5A), suggesting that many more rare genes can cause SNHL. The inheritance patterns of the 63 genes included autosomal recessive (26/63 genes; affecting 132 families; found double hits, including homozygote and compound heterozygote variants), autosomal dominant (33/63 genes; affecting 73 families; found single hit), X-linked (4/63 genes; affecting 6 families), and mitochondrial (3/63 genes; affecting 8 families) (Figure 3A). Within our cohort, three identified genes—*TECTA* (ATS3A and ATS3B), *COL4A3* (DFNA8/12 and DFNB21), and *WFS1* (DFNA6/14/38 and Wolfram syndrome)—are known to have exhibited both autosomal recessive and autosomal dominant inheritance patterns (https://hereditaryhearingloss.org/). To elucidate the mode of inheritance for each patient in our cohort, we analyzed their pedigree information and conducted cascade screening using Sanger sequencing. Among 15 unrelated families carrying *COL4A3*, *TECTA*, and *WFS1* variants, we comprehensively characterized the inheritance patterns, which were segregated as either recessive or dominant traits (Figure S6). Moreover, one patient exhibited dual genetic etiologies, inherited from their parents, harboring compound heterozygous variants (c.299del:p.His100LeufsTer12 and c.235del:p.Leu79CysfsTer3) in *GJB2* and a heterozygous variant (c.113G>A; p.Gly38Asp) in *COCH* (Figure S7).

Figures 3B and S5B display the mutational landscapes of the 352 causative variants, including the variant type. Among the 352 identified variants, we found missense variants (157, 44.6%), nonsense variants (51, 14.5%), frameshift variants (63, 17.9%), and inframe variants (10, 2.8%) within the exome region. Additionally, splicing variants (27, 7.7%), SVs (36, 10.2%), and mitochondrial variants (8, 2.3%) were identified. Interestingly, we observed differences in the distribution of variant types across genes. In particular, consistent with previous reports, SVs accounted for (20/30, 66.7%) of the total variants in the *STRC* gene.

Among the 190 causative variants (collapsed from 352 total variants by merging identical ones), 83 variants were novel (Figure 3C). All of the 352 variants were either classified as pathogenic (P) or likely pathogenic (LP) according to the American College of Medical Genetics and Genomics (ACMG) and Association for Molecular Pathology (AMP) guidelines or remained as VUSs but were considered highly suspected based on strong clinical and/or functional evidence (Table S6).^30^^,^^31^ A variety of functional studies—ranging from molecular modeling to minigene splicing assay—were performed to test 16 highly suggestive variants (Tables S7, S8, and S9), leading to the reclassification of 13 variants from “uncertain significance” to “likely pathogenic.” An analysis of the variant-type distribution among the 83 novel variants revealed that SVs, detected mostly through WGS, accounted for a much larger proportion (12.0%) compared to previously reported variants (2.8%) (Figure 3D). In addition, we observed an increasing trend in the proportion of patients with novel variants as the steps progressed from step 1 to step 3-2 (Figure 3E).

Novel SVs identified from WGS include exonic deletions on *SPATA5* and *CLCNKA* (Figures 3F, 3G, and S10). In one patient (SNUH 799), WGS identified a small SV (c.2227-3015_2354+1415del) exclusively involving exon 15 of *SPATA5* (Figure 3F), along with an in *trans* short frameshift deletion in exon 16 (c.2679_2680del). Notably, consistent with a previous case report,^32^ this patient exhibited systemic clinical manifestations, including bilateral moderately severe SNHL, intractable epilepsy with diffuse brain atrophy, and global developmental delay (Figure S11A). Next, we investigated the molecular consequences of the small SV (c.2227-3015_2354+1415del) on *SPATA5*-dependent bioenergetics using Seahorse assays (Figure S11B). The oxygen consumption rate (OCR) showed reduced respiratory function in patient fibroblasts, with significant decreases observed in basal respiration (*p* < 0.001), maximal respiration (*p* < 0.001), and ATP production (*p* < 0.001) compared to control mother fibroblasts (Figure S11C). These findings suggest that the small deletion in *SPATA5* (c.2227-3015_2354+1415del) contributes to impaired mitochondrial function, leading to SNHL.

In two additional patients (SNUH 420 and SNUH 481), WGS identified a deletion spanning *CLCNKA* and *CLCNKB* (g.[16032250_16046349del]) along with an in *trans* nonsense variant (c.778C>T; p.Gln260Ter) on *CLCNKA* (Table S2; Figure 3G). Although the deletion involved both genes, a combined analysis with nearby copy-number variations (CNVs) revealed an additional duplication in the *CLCNKB* region, resulting in a copy-number loss only in *CLCNKA*, not in *CLCNKB* (Figure S10B). These patients showed ns-SNHL without hypokalemic alkalosis or renal anomalies, suggesting a distinct ClC-K channel-related phenotype in which SNHL is the predominant feature.

### Advanced WGS approach revealed additional deep intronic variants

**Figure 4:**
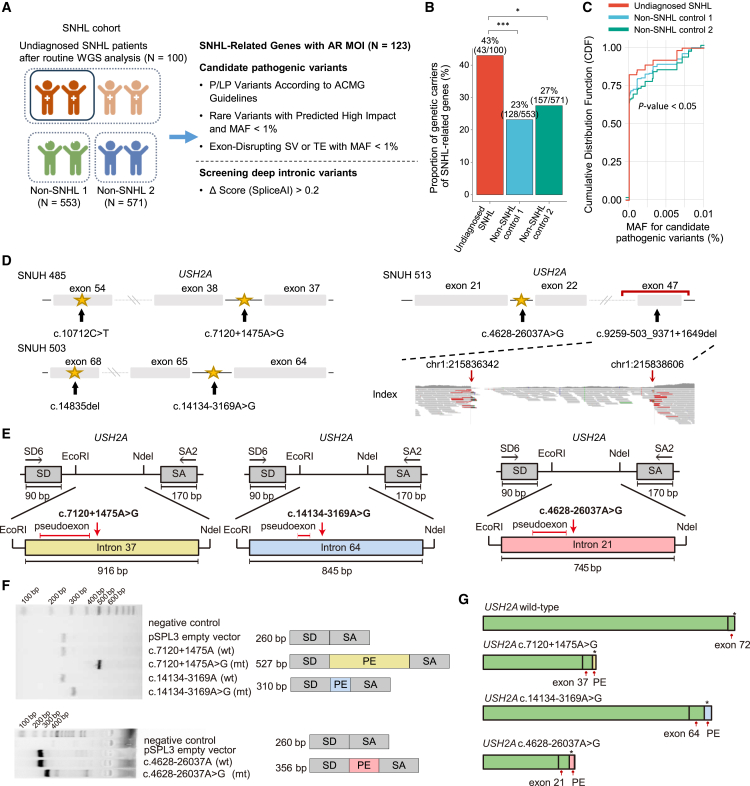
Analysis of carrier and deep intronic variants in undiagnosed patients with SNHL (A) Schematic diagram illustrating carrier status identification and screening for pathogenic deep intronic variants. (B) Bar plot showing the proportion of genetic carriers for SNHL-related genes across each cohort. (C) Cumulative distribution plot showing the MAF of candidate pathogenic variants across cohorts. (D) Schematic illustration of the location of each identified pathogenic variant within *USH2A* in each patient. (E) Schematic diagram of the pSPL3 vector with *USH2A* c.7120+1475A>G (left), c.14134-3169A>G (middle), and c.4628-26037A>G (right). (F) Electrophoresis gel image showing the bands corresponding to the pSPL3 empty vector (263 bp), each variant, and wild type. Representative results from at least three independent transfections and RT-PCR experiments are shown. (G) Schematic representation of the splice products with the wild-type splicing profile and the splice variant profiles for each mutant type. SNHL, sensory neural hearing loss; WGS, whole-genome sequencing; AR, autosomal recessive; MOI, mode of inheritance; P, pathogenic; LP, likely pathogenic; MAF, minor-allele frequency; SV, structural variation; TE, transposable element; SD, splicing donor; SA, splicing acceptor; PE, pseudoexon.

We sought to analyze the variant status of undiagnosed patients with SNHL, even after the automated WGS pipeline (step 3-1 in Figure 1) had been performed. We hypothesized that some undiagnosed patients with SNHL carrying a single heterozygous variant in autosomal recessive SNHL-related genes might harbor an additional, undetected pathogenic variant. Based on this hypothesis, we examined the carrier frequency of autosomal recessive SNHL-related genes (Figure S12A) in these undiagnosed patients, comparing it with that of control cohorts (non-SNHL1: *n* = 553, non-SNHL2: *n* = 571; Figure 4A). To identify candidate pathogenic variants, we focused on ACMG-classified pathogenic/likely pathogenic variants and rare variants (minor-allele frequency [MAF] < 1%) predicted to have a high functional impact. These variants with a high functional impact included those affecting transcript ablation, splicing, start codon loss, premature stop codon (gained/lost), frameshift, transcript amplification, structural modifications (elongation/truncation), and exon-disrupting SVs or transposable elements (TEs). For patients with SNHL who carried a single pathogenic variant after step 3-1, we further investigated whether an additional pathogenic variant existed on the opposite allele in step 3-2. To achieve this, we systematically screened intronic variants (MAF < 1%) in these SNHL carriers using *in silico* splicing predictions (e.g., SpliceAI)^33^ to detect potential splice-disrupting variants that may have been overlooked in prior analyses.

The analysis revealed that the carrier rate for pathogenic variants in autosomal recessive SNHL-related genes was significantly higher in the undiagnosed SNHL patient group compared to the two control cohorts (Figure 4B; Table S8). Additionally, an evaluation of the MAF of the identified candidate pathogenic variants showed that variants found in the undiagnosed SNHL group were rarer than those in the control group (Figure 4C). Subsequent screening for deep intronic variants in undiagnosed patients with SNHL identified three in *trans* variants with SpliceAI prediction scores greater than 0.2. These included deep intronic variants in *USH2A* (c.7120+1475A>G, c.14134-3169A>G, and c.4628-26037A>G), each found in different patients (SNUH 485, SNUH 503, and SNUH 513, respectively; Figures 4D, S12B, S12C, and S13). To evaluate their pathogenicity, minigene assays were designed with specific splice donor (SD) and splice acceptor (SA) sites. The assays revealed that these variants induce aberrant splicing that leads to the inclusion of pseudoexons of varying sizes: 267 bp for c.7120+1475A>G, 50 bp for c.14134-3169A>G, and 96 bp for c.4628-26037A>G (Figures 4 and 4F). The resulting aberrant transcripts are predicted to contain premature stop codons, leading to truncated, non-functional usherin protein (Figures 4G and S14D). Supporting this, a restriction enzyme digestion assay using EcoRI and NdeI confirmed the successful insertion of *USH2A* intron sequences into the empty vector (Figure S14). Combined with the first hits of the coding variant in this *USH2A* gene (c.10712C>T, c.14835del, and coding deletion, respectively), these alleles of *USH2A* were inactivated in these patients.

### Cost and turnaround time analysis for clinical practice

When diagnosing rare genetic diseases, cost and turnaround time (TAT) play a crucial role in clinical practice alongside final diagnostic yield. To assess the efficiency of different genetic diagnostic strategies, we analyzed the cost and TAT associated with each step in the testing process (Figure S15). The simulations were designed to compare three different genetic diagnostic approaches and determine the most time- and cost-effective strategy for identifying specific clinical scenarios. In our comparison, simulation 1 involved a stepwise diagnostic process used in our cohort, simulation 2 consisted of a direct WES followed by WGS without intermediate tests, and simulation 3 was a direct WGS without any intermediate tests. According to the results of the simulations, the cost was consistently estimated at approximately $1,000–$1,100 across different simulations, showing minimal variation. However, in terms of TAT, simulation 1 and simulation 2 required approximately 6–7 weeks, whereas simulation 3 yielded a shorter TAT of 4 weeks. Based on these findings, direct WGS without intermediate testing (simulation 3) appears to be the most efficient strategy, providing the shortest TAT while maintaining comparable costs.

### Genotype-phenotype correlations through molecular-function-based gene clustering

Based on the genetic diagnoses identified through comprehensive genetic analysis, we investigate the relationship between the affected genes and clinical manifestations. We found that causal genes of SNHL were closely linked to specific clinical manifestations, with 22 genes contributing to clinical manifestations in ≥3 families (Figure S16A). Through this analysis, we found many examples where mutations in the same gene resulted in similar phenotypes (Figure S16B). Information about the genes associated with distinct clinical phenotypes may aid in conducting in-depth genetic analyses within the highly heterogeneous genetic landscape of SNHL. However, variable expressivity and incomplete penetrance were documented even within identical genes and variants. Specifically, we investigated phenotypic heterogeneity in our cohort’s five most recurrent genes (*GJB2*, *SLC26A4*, *STRC*, *USH2A*, and *CDH23*) (Figure S17A). In particular, *USH2A* variants displayed genotype-phenotype segregation (e.g., allelic hierarchy) (Figure S17B)^34^: functional null alleles were associated with earlier, severe hearing loss, while two hypomorph alleles in *trans* often caused delayed, progressive hearing loss with retinitis pigmentosa. Variable expressivity was also documented among individuals with identical pathogenic variants in *GJB2* and *SLC26A4* (Figure S17B). For example, individuals homozygous for *SLC26A4* p.His723Arg showed considerable residual hearing yet experienced pronounced progression, interaural asymmetry, and variability in onset. Additionally, patients carrying at least one *GJB2* p.Val37Ile hypomorph allele^28^^,^^35^ had significantly milder hearing loss compared to those without this allele (*p* = 0.001 by Fisher’s exact test) (Figure S17C).

**Figure 5:**
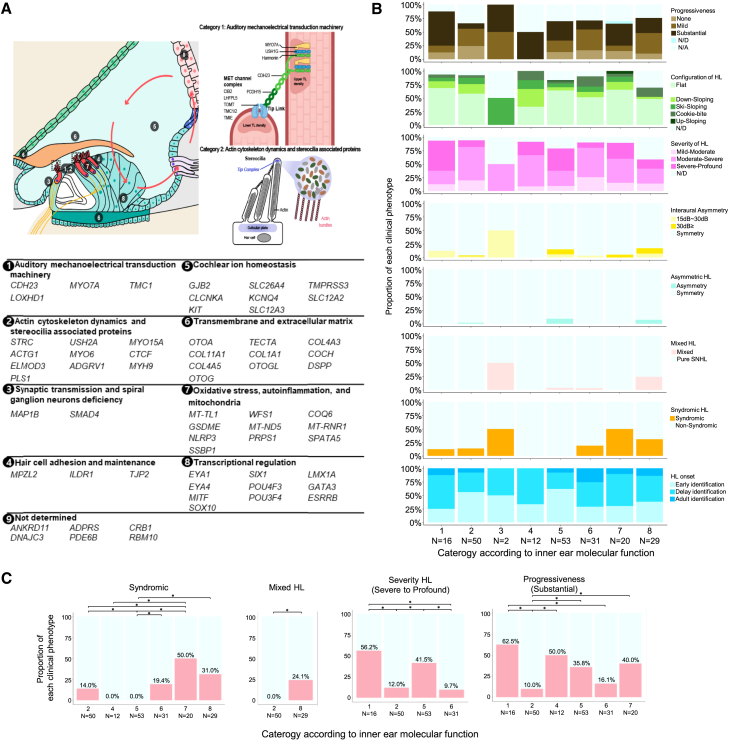
Functional classification and clinical relevance of SNHL-related genes (A) Schematic illustration of nine functional categories based on molecular mechanisms underlying inner ear function (top), along with a list of 63 genes identified in this study grouped into these categories (bottom). The dashed box in the stereocilia is zoomed to highlight category 1 and category 2. (B) Comparison of clinical phenotypic features across eight inner ear functional categories (excluding category 9: not determined). Cases with only one audiogram or a follow-up duration of less than 1 year were classified as “not available” (N/A) for hearing loss progression. Profound SNHL with thresholds ≥ 90 dB at 500 Hz on the initial audiogram was classified as “not determined” (N/D) for hearing loss progression. In cases with bilateral ear discrepancies in progressiveness, the overall category was defined as “substantial” if at least one ear showed substantial progression (e.g., substantial/mild, substantial/none, and substantial/N/D). If no ear was classified as substantial but at least one ear was classified as mild (e.g., mild/none and mild/N/D), the overall category was defined as “mild.” Furthermore, patients with mixed or asymmetric hearing loss were classified as N/D when analyzing severity and configuration of hearing loss. (C) Significant associations between clinical phenotypes and the eight functional categories; red bars indicate the proportion of affected probands within each category (refer to details in Table S10). Asterisks (∗) denote statistical significance (*p* < 0.05).

Based on these genotype-phenotype correlations, we grouped causal genes by their known function in the inner ear (Figure 5A). The functional framework used here derives from previously established categorizations for ns-SNHL genes based on mouse models and functional assays.^36^^,^^37^ Additionally, we refined the classification of the 63 deafness-related genes identified in our cohort by evaluating 21 newly identified genes that had not been previously categorized (Table S9). For four genes (*KCNQ4*, *MYO6*, *LMX1A*, and *POU4F3*), which could belong to multiple categories, we assigned them to the most dominant category. Overall, the 63 SNHL-related genes were classified into nine categories based on their molecular functions in the inner ear: (1) auditory mechanoelectrical transduction (MET) machinery; (2) actin cytoskeleton dynamics and stereocilia-associated proteins; (3) synaptic transmission; (4) hair cell adhesion and maintenance; (5) cochlear ion homeostasis; (6) transmembrane and extracellular matrix; (7) oxidative stress, autoinflammation, and mitochondrial defect; (8) transcriptional regulation; and (9) not determined. To evaluate the relationships among these categories, we compared gene expression patterns using publicly available transcriptome data from human inner ear organoids and from human cochlear and vestibular tissues.^38^ A comparative analysis of gene expression patterns using perturbation testing suggested a potential trend (*p* = 0.12) across the gene categories, though statistical significance was not reached (Figure S18A).

Interestingly, a post hoc analysis revealed that four phenotypic attributes (syndromic features, mixed hearing loss, severity of hearing loss, and progression) were significantly associated with pathogenic variants across the categories (Figures 5B and 5C; Table S10). Notably, genes more prevalent among patients with syndromic features were significantly enriched in categories 7 and 8. These observations align with the cell-type specificities of the genes, as those in categories 7 and 8 are broadly expressed across multiple organs and cell types (Figure S18B). Genes associated with mixed-type manifestations (e.g., *POU3F4*, *EYA1*, and *SIX1*) were more prevalent in category 8 than in category 2 (*p* = 0.02 by Fisher’s exact test with false discovery rate adjustment). We speculate that genes involved in transcriptional regulation influence the development and morphology of mammalian middle ear ossicles^39^ and the presence of a “third window” in the inner ear (i.e., EVAs),^40^ thus contributing to the conductive component of hearing loss. Furthermore, genes associated with severe or profound hearing loss were more predominant in categories 1 and 5 than in categories 2 and 6 (*p* = 0.01 by Fisher’s exact test with false discovery rate adjustment). Additionally, genes associated with substantial progressive hearing loss were more predominant in categories 1, 4, 5, and 7 than in categories 2 and 6 (*p* = 0.01 by Fisher’s exact test with false discovery rate adjustment). To support Figure 5C, additional plots have been provided in Figure S19, illustrating the proportions of causative genes in each category that align with specific phenotypes (e.g., penetrance), along with corresponding statistical significance. Further, we observed that zygosity (e.g., autosomal dominant vs. autosomal recessive/X-linked hemizygous) and variant type (e.g., truncating vs. non-truncating) influenced clinical phenotypes within the same functional category (Figures S20A and S20B). Collectively, these data support the development of a comprehensive genotype-phenotype map of SNHL and shed light on insights of previously undefined genotype-phenotype correlations.

## Discussion

Unlike previous cohort studies limited by the heterogeneous nature of phenotypes of various rare diseases,^41^^,^^42^ our present findings provide a robust estimate of diagnostic rates through a stepwise genetic testing approach from single-gene analysis to WGS, integrated with functional assays and bioinformatic analysis, in a relatively large cohort of patients with a single phenotype of SNHL. Herein, we demonstrated an additional diagnostic yield of 19.2% (23/120) through the systematic application of WGS in previously undiagnosed patients with SNHL who had undergone exome sequencing and targeted assays. Specifically, even among undiagnosed patients with SNHL following an automated WGS pipeline (step 3-1), we observed that the frequency of genome-wide single pathogenic alleles in known recessive deafness genes was higher than in control cohorts. Based on these findings, we hypothesized that this elevated frequency of single pathogenic alleles (43%) is less likely to be due to incidental carrier variants and instead suggests the presence of a second, undetected hit in the opposite allele. Consequently, through SpliceAI-based deep intronic variant analysis (step 3-2), we identified three meaningful deep intronic variants in *USH2A* among 100 patients. These findings also suggest the potential for further diagnosis of undiagnosed cases through additional molecular diagnostic approaches, such as methylation or proteomics analyses, thereby accelerating the overall diagnostic yield. Supporting this, Lunke et al. have shown the potential of the integration of multi-omic approaches into genomic testing, leading to additional diagnoses and changed critical care management.^43^

We identified the genetic causes of SNHL in 55.6% of our cohort families, with WGS and SpliceAI-based deep intronic variant analysis increasing the overall diagnostic yield by more than 5%. The improved diagnostic yield through WGS and in-depth analysis has been made possible by screening regions that are challenging to detect with conventional methods, primarily including deep intronic variants, small SVs, copy-neutral inversions, and complex genomic rearrangements. Among the 23 families with additional diagnoses identified through WGS and in-depth analysis, 16 cases (69.6%) could be diagnosed through reanalysis of targeted sequencing data and exome-based CNV algorithms. However, 7 (30.4%) of these cases required WGS for a definitive diagnosis. Our study further provides clinical guidelines for selecting patients with SNHL who are most likely to benefit from WGS. WGS shows a tendency to be more effective for genetic completion in patients with SNHL with early-onset or syndromic features, particularly when trio-based WGS is performed, leading to a higher diagnostic yield. However, these findings should be interpreted with caution due to potential selection bias, as WGS was performed only in patients who remained undiagnosed after prior analyses. Further studies are needed to validate the applicability and utility of WGS across different SNHL phenotypes.

Using the simple simulation, we demonstrated that direct WGS can be an efficient strategy to quickly diagnose patients with SNHL (Figure S15). Given the ongoing clinical trials of AAV-OTOF gene therapy worldwide,^44^^,^^45^ direct WGS without intermediate testing may be necessary for pediatric patients exhibiting the phenotype of auditory neuropathy. Since WGS enables accurate identification of bi-allelic *OTOF* variants, it is expected to facilitate more rapid and precise identification of eligible patients for enrollment in AAV-OTOF gene therapy clinical trials. Furthermore, the timing of pediatric cochlear implantation has been progressively advancing, with procedures now being performed as early as 6 months of age.^46^^,^^47^ Identifying the precise genetic cause of hearing loss before cochlear implantation plays a crucial role in predicting outcomes and optimizing post-implantation management.^48^ Therefore, in patients with congenital hearing loss with bilateral profound SNHL, direct WGS without intermediate testing is anticipated to expedite the decision-making process for cochlear implantation and facilitate earlier intervention.

Genetic information could serve as a guide to clinical phenotypes and their natural course, highlighting the importance of WGS in identifying additional genetic causes in undiagnosed patients. In detail, early identification of genetic causes may be necessary for detecting preclinical symptoms (e.g., ns-SNHL mimics)^34^ and for providing reproductive counseling, including guidance on next-baby planning and options for preimplantation genetic testing, even in cases of non-syndromic hearing loss.^49^ Although targeted agents based on genotype are not yet commonly established in many human genetic disorders, there have been significant advances in personalized, targeted therapy in recent years, including in the field of genetic hearing loss.^44^^,^^45^^,^^50^^,^^51^^,^^52^ For example, three presently identified genomic variants in the deep intronic region of *USH2A* are targetable by splice-switching antisense oligonucleotide (ASO) therapy, offering an opportunity to slow down or even halt disease progression in these patients. According to a framework for individualized splice-switching ASO therapy,^53^ the *USH2A* deep intronic variants that induce pseudoexon inclusion without disrupting cryptic splicing sites were highly amenable to ASO splice modulation. Furthermore, in theory, a subset of SVs—such as the *EYA1* paracentric inversion (SNUH 734) and *EYA1* complex genomic rearrangements (SNUH 536) linked to haploinsufficiency detected in WGS—can be corrected using CRISPR-based editing approaches, including Cas9 nuclease with paired gRNAs, CRISPR activation, and the prime editing strategy.^54^^,^^55^^,^^56^ The present study provides good examples of the potential of inner ear precision medicine for SNHL treatment, with broadened therapeutic targets identified through WGS and in-depth analysis.

Our comprehensive genomic investigation further refined the genotype-phenotype landscape of SNHL, revealing gene signatures based on phenotypes. The distribution of causative genes identified in this study largely aligns with findings from exome-based SNHL cohort studies. While this genetic information could support genetic diagnosis and provide a rationale for in-depth analysis in the clinical setting, further studies with larger cohorts are essential to establish more specific phenotype-genotype correlations that account for variant effects (e.g., allelic hierarchy).

Furthermore, we found that genotype-phenotype correlations were also present at the level of the molecular pathways of the genes in the inner ear. Many of these relationships are consistent with previous functional studies. For example, major genes in category 1 are the most frequent causes of congenital or prelingual severe-to-profound hearing loss (e.g., *CDH23*-associated DFNB12),^57^ which often necessitates cochlear implantation. Given that MET current is essential for maintaining the functional properties of hair cells during maturation^58^ and for regulating membrane homeostasis of the MET channel, which correlates with deafness phenotype,^59^ it is reasonable to see that category 1 genes result in severe and substantial progression of hearing loss. Related to this, a *Cdh23* exon 68 deletion leading to a loss-of-function allele compromises tip-link stability and causes progressive hearing loss,^60^ and *Myo7a* is critical for preserving the structural and functional integrity of the MET complex in adult cochlear hair cells.^61^ Additionally, *MPZL2* in category 4,^62^ *SLC26A4* in category 5,^63^ and mitochondrial machinery-related genes in category 7^64^^,^^65^^,^^66^ are associated with progressive hearing loss in the previous studies. Further, major genes in category 5 (i.e., *GJB2*, *SLC26A4*, and *TMPRSS3*) also represent the most common and primary candidates for cochlear implantation.^48^ By contrast, genes in categories 2 and 6 are predominantly associated with mild-to-moderate SNHL. For example, *STRC* in category 2, the most common gene implicated in mild-to-moderate SNHL, is generally considered non-progressive.^67^^,^^68^ Likewise, *TECTA*, *OTOG*, and *OTOGL* in category 6, which encode proteins forming links between outer hair cell stereocilia and the tectorial membrane, are reported to exhibit a little or very slow progression of hearing loss,^69^ consistent with preclinical evidence from genetically engineered mouse models.^70^^,^^71^

This categorization of genetic hearing loss suggests that genes within each functional class exhibit not only distinct inner ear molecular functions but also relatively homogeneous spatial expression patterns in the cochlea. Unlike traditional genotype-phenotype correlations, which are often limited by specific ethnicities, genotypes, or phenotypes, the classification based on inner ear molecular pathways expands beyond the current understanding of SNHL and provides insights for predicting phenotypes associated with newly identified deafness genes.

Since the introduction of the ACMG guidelines, variant classification has become more standardized, with ongoing refinements through ClinGen enabling disease-specific adaptations.^72^^,^^73^ Various tools incorporating these standards are now available and were utilized in this study for variant classification (Table S6).^30^^,^^31^ However, manual curation remains indispensable, as no single tool currently offers a fully comprehensive solution. Certain variants remain challenging to classify, particularly in conditions with overlapping syndromic and non-syndromic manifestations, such as SNHL. The alignment between genetic findings and clinical presentation remains a critical factor in interpretation. As more patient data accumulate and analytical tools advance, the ability to resolve ambiguous cases will continue to improve, ultimately enhancing genotype-phenotype correlations in SNHL.

Collectively, our results provide evidence for the clinical utility of the integrated diagnostic approaches, including WGS, in real-world SNHL practice. However, despite these systematic evaluations, more than 40% of patients still lack a definitive genetic diagnosis, highlighting the need for further research to identify additional causative variants. Future studies incorporating multi-omics approaches, long-read sequencing, and advanced computational tools will be essential to addressing these gaps. These advancements will lead to a more thorough understanding of genomic architectures and their associated phenotypic attributes, paving the way for the future of precision medicine.

### Limitations of the study

While this study demonstrates the clinical utility of an integrated genomic approach in SNHL, several limitations should be acknowledged. First, WGS was applied only to cases that remained undiagnosed after prior targeted or exome-based analyses, potentially introducing selection bias and limiting the generalizability of our findings across the full spectrum of SNHL phenotypes. Second, although the stepwise pipeline enabled the identification of additional diagnoses, more than 40% of cases remained genetically unresolved, underscoring current limitations in sequencing technologies and variant interpretation frameworks. Certain pathogenic variants—including deep intronic, structural, and complex rearrangements—may still escape detection due to the resolution limits of short-read WGS and the lack of robust analytical tools. Lastly, although our genotype-phenotype correlations revealed informative trends at the functional category level, larger and more diverse cohorts will be required to refine these associations and validate their predictive power in broader clinical contexts.

## Resource availability

### Lead contact

Any further information or requests should be directed to and will be fulfilled by the lead contact, June-Young Koh (jy.koh@inocras.com).

### Materials availability

This study did not generate new unique reagents.

### Data and code availability

Statistical analyses and visualizations were conducted using R v.4.2.2, and the corresponding code can be accessed at https://github.com/SNUH-hEARgeneLab/WGS_analysis.

The individual patient and sequencing data reported in this paper cannot be deposited in a public repository because of the patient’s genetic information, the possibility of privacy invasion, psychosocial risks (such as social stigma and discrimination), and the institutional review board (IRB)’s restrictions on public data release. To access the data, please submit a request to the lead contact (jy.koh@inocras.com). The request will be reviewed, and, if approved, the lead contact will work with the requestor on sharing the data and by adhering to the consent agreements established with the study participants. Any additional information required to reanalyze the data reported in this paper is available from the lead contact upon request.

## Acknowledgments

This research was supported and funded by the SNUH Kun-hee Lee Child Cancer & Rare Disease Project, Republic of Korea (grant nos. 25C-059-0100 to S.-Y.L. and 22B-001-0100 to J.-H.C.); a Phase III (postdoctoral fellowship) grant of the SPST (SNU-SNUH Physician Scientist Training) Program (S.-Y.L.); the National Research Foundation of Korea (NRF), funded by the Ministry of Education (grant no. 2022R1C1C1003147 to S.-Y.L.); the SNUH Research Fund (grant nos. 37-2023-0120 and 04-2021-0670 to S.-Y.L.); and the Seoul R&BD Program (grant no. BT240056 to S.-Y.L.). This research was supported by a grant of the Korea Health Technology R&D Project through the Korea Health Industry Development Institute (KHIDI), funded by the Ministry of Health & Welfare, Republic of Korea (grant no. RS-2024-00440385 to J.-Y.K.).

## Author contributions

Concept and design, S.-Y.L. and J.-H.C.; acquisition, analysis, or interpretation of data, S.-Y.L., Seungbok Lee, S.P., J.-Y.K., S.H.J., Y.Y., W.H.C., J.H.C., and T.M.K.; drafting of the manuscript, S.-Y.L., Seungbok Lee, S.P., and J.-Y.K.; critical review of the manuscript for important intellectual content, S.-Y.L., Seungbok Lee, S.P., and J.-Y.K.; statistical analysis, S.-Y.L., S.P., and J.-Y.K.; obtaining funding, S.-Y.L. and J.-Y.K.; administrative, technical, or material support, H.Y., Sangmoon Lee, M.-W.S., M.K.P., J.-J.S., B.Y.C., and J.H.L.; supervision, J.-H.C. and Y.S.J.

## Declaration of interests

Y.S.J. is the founder of Inocras, Inc., a genome analysis and interpretation company. Y.S.J., J.-Y.K., S.P., and Sangmoon Lee hold stocks or stock options in Inocras, Inc.

## STAR★Methods

### Key resources table

**Table:** 

REAGENT or RESOURCE	SOURCE	IDENTIFIER
**Antibodies**		

Monoclonal ANTI-FLAG® M2 antibody	Sigma-Aldrich	F3165; RRID: AB_259529
Goat anti-Mouse IgG (H + L) Highly Cross-Adsorbed Secondary Antibody, Alexa Fluor™ Plus 488	Invitrogen	A32723; RRID: AB_2633275

**Chemicals, Peptides, and Recombinant Proteins**		

Dulbecco’s modified Eagle’s medium	WELGENE	LM001-05
Fetal bovine serum	Gibco	12483–020
Penicillin/streptomycin	WELGENE	LS015-01
L-glutamine	WELGENE	LS002-01
Dulbecco’s Phosphate-Buffered Salines (D-PBS)	WELGENE	LB001-02-500
Mounting Medium With DAPI	Abcam	ab104139
Bovine Serum Albumin	GenDEPOT	A0100-010
Concanavalin A (Con A) Conjugates (Alexa Fluor 633)	Invitrogen	C21402
Platinum™ SuperFi II PCR Master Mixes	Invitrogen	12369010
Taq DNA Polymerase	BIONEER	E−2011-1
Agarose	Promega	V3125
Dyne LoadingSTAR	Dyne Bio	A760

**Critical Commercial Assays**		

Lipofectamine 3000	Invitrogen	L3000008
EZ™ Total RNA Miniprep Kit	Enzynomics	EP301-50N
TRIzol™ Reagent	Invitrogen	15596026
AccuPower® RT PreMix	BIONEER	K-2041
AllPrep DNA/RNA Mini Kit	Qiagen	80204
Seahorse XF Cell Mito Stress Test Kit	Agilent	103015–100
TruSeq DNA PCR-Free Library Prep Kits	Illumina	20041715

**Experimental Models: Cell Lines**		

Human: HEK293T	ATCC (American Type Culture Collection)	CRL-3216

**Oligonucleotides**		

Minigene splicing assay primer pair: SD6 5′-TCTGAGTCACCTGGACAACC-3′ SA2 5′-ATCTCAGTGGTATTTGTGAGC-3′	This study	N/A
Human mt1 primer pair: hmtF1 569 5′-AACCAAACCCCAAAGACACC-3′ and hmtR1 9819 5′-GCCAATAATGACGTGAAGTCC-3'	This study	N/A
Human mt2 primer pair: htmF2 9611 5′- TCCCACTCCTAAACACATCC-3′ and hmtR2 626 5′- TTTATGGGGTGATGTGAGCC-3'	This study	N/A
GJB2_Exon1 primer pair F5′-CAGTCTCCGAGGGAAGAGG-3′ and R5′-GCAACCGCTCTGGGTCTC-3′	This study	N/A
GJB2_Exon2a primer pair F5′-GCATGCTTGCTTACCCAGAC-3′ and R5′-AGCCGTCGTACATGACATAGAAG-3′	This study	N/A
GJB2_Exon2b primer pair F5′-CTGCAGCTGATCTTCGTGTC-3′ and R5′-ATCCCTCTCATGCTGTCTATTTCT-3′	This study	N/A
KCNQ4_Exon9 primer pair F5′-GATGTCAGGGCCACTGCT-3′ and R5′-GCATGGACATCTCTCCCACT-3′	This study	N/A
COCH_Exon4 primer pair F5′-TTGCCAAAATCTGGAATGGT-3′ and R5′-TGTGTTTGGGCTTACCTGTG-3′	This study	N/A
USH2A_Exon54 primer pair F5′-TCAAGAATCCATCTCCTCCA-3′ and R5′-TTGGTTGGTGAGGAAAGAATG-3′	This study	N/A
USH2A_Intron37 primer pair F5′-CCGAACAGCCCTTGTAGAAA-3′ and R5′-TCCTCCAGCTCTACATAAATCACA-3′	This study	N/A
USH2A_Exon68 primer pair F5′-CACACCTGCACTCCTTCAAA-3′ and R5′-TAACTTTTGTCCGCCGTTCT-3′	This study	N/A
USH2A_Intron64 primer pair F5′-CCTGAATAAATCAAAGATGAAGAACA-3′ and R5′-CTGAGAAACAATGCCCCAAG-3′	This study	N/A
USH2A_Intron21 primer pair F5′-GCATATTCATTTCTCTCTCC-3′ and R5′-GCTTTTCAAGACTGAAGTC-3′	This study	N/A

**Recombinant DNA**		

Plasmid: pRK5-KCNQ4 wild-type-myc-flag	This study	N/A
Plasmid: pRK5-KCNQ4 R390C-myc-flag	This study	N/A
Plasmid: pSPL3 empty vector	NovoPro	V001167
Plasmid: pSPL3-USH2A c.7120 + 1475A (wt)	This study	N/A
Plasmid: pSPL3-USH2A c.7120 + 1475A>G (mt)	This study	N/A
Plasmid: pSPL3-USH2A c.14134-3169A (wt)	This study	N/A
Plasmid: pSPL3-USH2A c.14134-3169A>G (mut)	This study	N/A
Plasmid: pSPL3-USH2A c.4628-26037A (wt)	This study	N/A
Plasmid: pSPL3-USH2A c.4628-26037A>G (mt)	This study	N/A
Plasmid: pSPL3-DSPP c.51+5G (wt)	This study	N/A
Plasmid: pSPL3-DSPP c.51 + 5G>A (mt)	This study	N/A

**Software and Algorithms**		

CFX Manager	Bio-rad	Ver 3.1.1621
NextGene	Softgenetics	Ver 2.4.0.1
R	R-Project	Ver 4.2.2
Python	Python	Ver 3.10.4
SnapGene	SnamGene	Ver 8.0.2
RareVision	Inocras Inc.	N/A
BWA-MEM	https://github.com/lh3/bwa	Ver 0.7.15 (r1140)
SAMBLASTER	https://github.com/GregoryFaust/samblaster	Ver 0.1.26
HaplotypeCaller	https://gatk.broadinstitute.org/hc/en-us/articles/360037225632-HaplotypeCaller	Ver 4.1.4.1
Strelka2	https://github.com/Illumina/strelka	Ver 2.9.10
Delly	https://github.com/dellytools/delly	Ver 1.1.7
SpliceAI	https://github.com/Illumina/SpliceAI	Ver 1.3.1
IGV	Integrative Genomics Viewer	Ver 2.16.2
Code	This paper	https://github.com/SNUH-hEARgeneLab/WGS_analysis
Bio-Rad CFX manager	Bio-Rad Laboratories, Inc.	Ver 1.6
GeneMarker	SoftGenetics	Ver 1.91

### Experimental model and study participant details

#### Human subjects

This study included 750 individuals from 394 unrelated SNHL families, who were prospectively recruited at the Hereditary Hearing Loss Clinic within the Otorhinolaryngology division of the Center for Rare Diseases, Seoul National University Hospital, Korea, between March 2021 and February 2023. Clinical data, including onset of hearing loss, audiological profiles, and syndromic features, were retrieved from electronic medical records. Hearing loss onset was categorized into early identification (through failed newborn hearing screening), delayed pediatric identification (onset by age 18 but not detected at birth), or adult identification (onset after 18 years). Syndromic features were evaluated during the first outpatient clinic visit based on clinical manifestations and medical history, and associated medical conditions were classified using ICD-10 codes. Gender was recorded from medical records; however, no analyses based on gender were performed. All procedures involving human participants were approved by the Institutional Review Board of Seoul National University Hospital (approval numbers: IRB-H-0905-041-281 and IRB-H-2202-045-1298). Written informed consent was obtained from all participants or their legal guardians.

#### Cell lines

HEK293T cells were used for minigene splicing assays and *KCNQ4* functional studies. Cells were obtained from the American Type Culture Collection (ATCC) and authenticated based on morphology and growth characteristics; no short tandem repeat (STR) profiling was performed. Cells were regularly tested for mycoplasma contamination and consistently tested negative. HEK293T cells were cultured in Dulbecco’s Modified Eagle Medium (DMEM) supplemented with 10% fetal bovine serum (FBS) and 1% penicillin/streptomycin, and maintained at 37°C in a humidified atmosphere containing 5% CO_2_.

Primary fibroblast cultures were established from skin biopsies collected under local anesthesia from participants who provided written informed consent. The fibroblasts were authenticated based on morphology without additional genetic testing. Fibroblast cultures were regularly tested for mycoplasma contamination and confirmed to be negative. Cells were maintained in DMEM supplemented with 20% fetal bovine serum and incubated at 37°C in a 5% CO_2_ atmosphere.

### Method details

#### Study cohort

In this study, we utilized a prospective research design and focused on participants attending the Hereditary Hearing Loss Clinic within the Otorhinolaryngology division of the Center for Rare Diseases, Seoul National University Hospital, Korea, between March 2021 and February 2023. Patients were not included if they were referred from other centers with confirmed genetic diagnoses or diagnosed as conductive hearing loss. In total, our SNHL cohort comprised 394 unrelated families and 750 individuals including probands, who exhibited hearing loss with sensorineural components, and their family members. The demographic data and clinical phenotypes were retrieved from the electronic medical records. The onset of hearing loss was classified into three distinct categories^74^: early identification (i.e., congenital or prelingual deafness identified through failed newborn hearing screening test), delay identification (i.e., pediatric-onset deafness occurring by age 18 that does not meet the criteria for early identification, regardless of newborn hearing screening confirmation), and adult identification (i.e., documented adult-onset hearing loss). The syndromic features of the patients in the cohort were evaluated during their first outpatient clinic visit based on their medical histories and/or features in their clinical manifestations. The presence of associated medical conditions (e.g., syndromic hearing loss) was determined using the Tenth Revision of the International Statistical Classification of Diseases and Related Health Problems (ICD-10) codes. All procedures were approved by the Institutional Review Board of Seoul National University Hospital (no. IRB-H-0905-041-281 and IRB-H-2202-045-1298).

#### Audiological evaluation

Depending on the participant’s age, the hearing thresholds for six different octaves (0.25, 0.5, 1, 2, 4, and 8 kHz) were evaluated using pure-tone audiometry (PTA).^75^ For patients under 3 years of age or having neurodevelopmental delay, auditory brainstem response threshold (ABRT) and auditory steady-state response (ASSR) were used to gauge the thresholds at four-octave frequencies (0.5, 1, 2, and 4 kHz). The conductive components were evaluated using comprehensive tests, including tympanic membrane examination, tympanometry (probe tones of 226 and 1000 Hz), and/or bone conduction ABRT, particularly in younger subjects. Auditory profiles were retrieved—such as asymmetry, severity, configuration, and progression. The mean hearing threshold was determined using an average of the thresholds at 0.5, 1, 2, and 4 kHz, and the degree of hearing loss was categorized as mild-to-moderate (21–40 dB or ≤20 dB with high-frequency hearing loss), moderate-to-severe (41–70 dB, and severe-to-profound (≥71 dB).^75^ Audiogram configurations were categorized into one of five subtypes: down-sloping (i.e., consistent downward trend observed across 250, 500, 1000, 2000, and 4000 Hz frequencies, with an average threshold at 250 and 500Hz ≤ 40 dB), ski-sloping (i.e., thresholds at 250 Hz are ≤25 dB, with a decrease of ≥40 dB between 250 and 1 kHz or 500–2 kHz, or a decrease of ≥70 dB across 250–4 or 8 kHz), cookie-bite (i.e., U-shaped), up-sloping (i.e., rising), and flat (i.e., audiograms that does not fit down-sloping, ski-slope, cookie-bite, or up-sloping configurations).^76^ Asymmetric hearing loss was defined as severe-to-profound hearing loss in the poorer ear, with an average hearing threshold >30 dB HL and <55 dB HL in the better ear. The presence of interaural asymmetry (a difference in average between the poorer ear and the better ear of 15 to less than 30 dB, and a difference in average between the poorer ear and the better ear of 30 or more than 30 dB) was also assessed.^77^ To analyze hearing loss progression, serial audiograms were used to retrieve the hearing threshold at all frequencies. Hearing loss progression was assessed in cases with two or more audiograms documented during the follow-up period, with at least a one-year interval between documentation. Cases with only one audiogram or follow-up duration of less than 1-year were classified as not available (i.e., N/A). Profound SNHL with thresholds ≥90 dB at 500 Hz was classified as not determined (i.e., N/D). Hearing loss progression in this study was categorized as substantial (≥10 dB deterioration at three or more frequencies), mild (≥5 dB deterioration at three or more frequencies or ≥10 dB deterioration at one or two frequencies), and none (if neither substantial nor mild criteria were met).

#### Real-time PCR and GJB2 sequencing

Genomic DNA was extracted from peripheral blood samples utilizing the Chemagic 360 instrument (PerkinElmer, Baesweiler, Germany). Real-time PCR (PCR) was performed using the U-TOP HL Genotyping Kit Ver1 and Ver2, along with a CFX96 Real-Time PCR Detection System (Bio-Rad Laboratories, Inc., Hercules, CA, USA).^78^^,^^79^ This process was used to examine 22 pathogenic variants across 10 deafness genes. The data collected from this procedure were analyzed using Bio-Rad CFX manager v1.6 software. Variants were identified through the fluorescence signals from the detection probes, which corresponded to the melting temperature (Tm), as specified by the standard protocol in the manufacturer’s manual. We additionally conducted sequencing of the *GJB2* single gene, following a previously described method.^80^

#### Targeted panel sequencing and whole-exome sequencing

We utilized TPS or WES to sequence the exonic regions of SNHL-related genes. Specifically, ns-SNHL patients were initially recommended TPS; however, due to the relatively high cost of TPS (∼$1000) despite insurance coverage, some patients declined TPS and instead underwent WES supported by a rare disease project (FP-2022-00001-004). The target regions were captured using a SureSelect DNA targeted sequencing panel for TPS, and a SureSelectXT Human All Exon V5 for WES (Agilent Technologies, Santa Clara, CA, USA). A library was prepared following the manufacturer’s instructions, and was paired-end sequenced using a NovaSeq 6000 sequencing system (Illumina, San Diego, CA, USA).

Sequence reads were aligned to the human reference genome (GRCh38) and processed according to the Genome Analysis Toolkit (GATK) best-practice pipeline for calling single nucleotide variants (SNVs) and short insertions/deletions (indels).^81^ The ANNOVAR program was used for variant annotation, such as the RefSeq gene set and Genome Aggregation Database (gnomAD).^25^^,^^82^ Rare non-silent variants were selected as candidates, including nonsynonymous SNVs, coding indels, and splicing variants. We also used the Korean Reference Genome Database (KRGDB) and KOVA databases for further filtration of ethnic-specific variants.^26^^,^^83^ Additionally, the ClinVar and HGMD databases were screened to check whether candidate variants had been previously identified in other patients.^84^^,^^85^

We classified candidate variants according to the ACMG-AMP guidelines using the InterVar and VarSome programs,^30^^,^^31^ and manually curated the classifications following the modified guidelines for SNHL.^72^^,^^73^

#### Multiplex ligation-dependent probe amplification and mitochondria panel sequencing

For individuals displaying non-syndromic, symmetric, mild-to-moderate SNHL, we evaluated CNVs using the SALSA MLPA Probemix P461-B1 *STRC*-*CATSPER2*-*OTOA* (MRC-Holland, Amsterdam, Netherlands).^23^ Additionally, for patients showing evidence of EVA on temporal bone CT and/or internal acoustic canal MRI and clinical features of BOR/BO syndrome, we performed *SLC26A4* and *EYA1* MLPA tests, respectively, using the SALSA MLPA Probemix P280-B4 *SLC26A4* and the SALSA MLPA Probemix P153-B2 *EYA1* (MRC-Holland). We analyzed the amplification products using an ABI PRISM 3130 Genetic Analyzer (Applied Biosystems, Foster City, CA, USA) and interpreted the results using GeneMarker 1.91 software (SoftGenetics, State College, PA, USA).

For mitochondria panel sequencing, DNA was extracted from peripheral blood samples using the Chemagic 360 instrument (PerkinElmer, Baesweiler, Germany). The complete human mitochondrial genome was amplified in two overlapping fragments: fragment I (spanning 9,289 bp), and fragment II (spanning 7,626 bp). Fragment 1 was amplified using the primer pair hmtF1 569 (5′-AACCAAACCCCAAAGACACC-3′) and hmtR1 9819 (5′-GCCAATAATGACGTGAAGTCC-3′), and fragment II was amplified using the primer pair htmF2 9611 (5′-TCCCACTCCTAAACACATCC-3′) and hmtR2 626 (5′-TTTATGGGGTGATGTGAGCC-3′).^86^ PCR reactions were conducted using the following cycling parameters: initial denaturation at 94°C for 2 min; 10 cycles of 94°C for 15 s, 65°C for 30 s, and 68°C for 5 min; 25 cycles of 94°C for 15 s, 65°C for 30 s, and 68°C for 5 min; and a final extension at 68°C for 7 min. Subsequently, a library was generated using the Nextera DNA Flex Library Prep Kit (Illumina) following the manufacturer’s instructions. Paired-end sequencing was performed with the generation of 150-bp reads on the MiSeq platform (Illumina). Bioinformatic processes, including alignment and annotation, were performed using NextGene Version 2.4.0.1 (Softgenetics).

#### Selection of the target population for whole-genome sequencing

All patients with s-SNHL (*n* = 21) who remained undiagnosed after exome sequencing and other techniques underwent WGS. Conversely, in patients with ns-SNHL (*n* = 177) who remained undiagnosed, we determined the sample representativeness for WGS. First, we estimated the sample size with a 7% margin of error and a 95% CI. Second, we employed a probability sampling method, specifically stratified sampling, considering a significant heterogeneity of SNHL patients with respect to audiological characteristics. Relevant covariates, including SNHL onset, severity, and asymmetry phenotypes, were used as criteria for stratification. Thus, for the 177 undiagnosed ns-SNHL patients, a representative validation was conducted, and WGS was ultimately performed on 99 families, exceeding the required sample size of 94, which corresponds to a 7% margin of error and a 95% CI. A representative sample was then obtained by randomly sampling within each stratum. Chi-square tests were conducted to assess differences across these strata. Overall, our methodology combines a well-thought-out sample size estimation with a stratified sampling approach and proper statistical validation, making it a robust approach for selecting a representative sample for WGS in undiagnosed ns-SNHL patients.

#### Library construction and automated analytic pipeline for whole-genome sequencing

To obtain genomic DNA, peripheral blood samples were collected from probands with or without their parents. The entire process of genome sequencing, analysis, and interpretation was performed using the RareVision system (Inocras, San Diego, CA, USA). Genomic DNA was extracted from blood samples using the Allprep DNA/RNA kits (Qiagen, Venlo, Netherlands). DNA libraries were prepared using TruSeq DNA PCR-Free Library Prep Kits (Illumina) and sequenced on the Illumina NovaSeq6000 platform with an average depth of coverage of 30×. The obtained genome sequences were aligned to the human reference genome (GRCh38) using the BWA-MEM algorithm. PCR duplicates were removed using SAMBLASTER.^87^ The initial mutation calling for base substitutions and short indels was performed using HaplotypeCaller and Strelka2, respectively.^88^ SVs were identified using Delly. Variants were filtered, and their Mendelian inheritance patterns were assessed. *De novo* mutations were detected, and their potential impacts were predicted. The pathogenicity prediction was further enhanced by using in-house-developed software that automatically integrates updated databases. The final evaluation of variant pathogenicity was determined by medical geneticists, considering the patient’s phenotype and familial history.

#### *In vitro* splicing analysis using minigene assay

Fragments carrying *USH2A* intron 37 (reduced to 916 bp) with c.7120 + 1475A or c.7120 + 1475A>G and intron 64 (reduced to 845 bp) with c.14135-3169A or c.14135-3169A>G were amplified and cloned into the pSPL3 vector between the exon splice donor (SD) and splice acceptor (SA), using the EcoRI and NdeI restriction sites. *USH2A* intron 21 (reduced to 745 bp) with c.4628-26037A or c.4628-26037A>G was cloned into the pSPL3 vector using EcoRI and BamHI restriction sites. For the *DSPP* variant (c.51 + 5G>A), exon 2 of the *DSPP* gene, along with its flanking intronic sequences containing wild-type alleles, was amplified using specific primers incorporating EcoRI and NdeI restriction sites. The amplified product was then cloned into the pSPL3 vector, and direct sequencing verified the accuracy of the constructs. Site-directed mutagenesis was subsequently performed on the pSPL3-DSPP wild-type vector to generate a construct carrying the mutant allele.Human epithelial kidney 293T (HEK293T) cells were seeded in a six-well culture plate and incubated at 37°C in a 5% CO_2_ atmosphere in Dulbecco’s modified Eagle’s medium (LM001-05; Welgene, Gyeongsan, Korea) containing 10% fetal bovine serum (12483-020; Gibco, Carlsbad, CA, USA), 100 units/mL penicillin/streptomycin (LS015-01; Welgene), and 2 mM L-glutamine (LS002-01; Welgene). On the next day, the cells were transfected with 2 μg pSPL3 plasmid per 1 well in 6-well plate using Lipofectamine 3000 reagent (L3000001; Invitrogen, Carlsbad, CA, USA), according to the manufacturer’s guidelines. After 24 h, the cells were harvested, and the total RNA was extracted using TRIzol Reagent (15596026; Invitrogen) and chloroform. From 1 μg RNA, cDNA was prepared by reverse transcription using the Accupower RT-preMix (K-2041; Bioneer, Daejeon, Korea). Splicing analysis was performed by PCR amplification with Taq DNA Polymerase (E−2011-1; Bioneer) or Platinum SuperFi II PCR Master Mixes (12369010; Invitrogen) using the following vector-specific primers: SD6 (5ʹ-TCTGAGTCACCTGGACAACC-3ʹ) and SA2 (5ʹ-ATCTCAGTGGTATTTGTGAGC-3ʹ).

#### Fibroblast cell culture

A skin biopsy was obtained from a donor under local anesthesia and preserved in Phosphate Buffered Saline (PBS). The biopsy was divided into 9–12 distinct segments and seeded into a 12-well plate containing DMEM supplemented with 20% FBS. Once the segments reached confluence, fibroblasts were harvested for further expansion. The fibroblast cultures were regularly tested for mycoplasma contamination and confirmed to be negative.

#### Oxygen consumption rate

Cellular OCR was measured in real-time using the Seahorse XF96 Extracellular Flux Analyzer (Seahorse Bioscience, North Billerica, MA, USA) per the manufacturer’s protocol. Cells (8.0 × 10ˆ3 fibroblasts) were seeded in 100 μL of growth medium in Seahorse 96-well microplates and incubated at 37°C with 5% CO2 for 24 h. Prior to the assay, cells were washed with assay running media (unbuffered DMEM supplemented with 25 mM glucose, 1 mM glutamine, and 1 mM sodium pyruvate) and equilibrated in a non-CO2 incubator overnight. Calibration of the assay plate was performed overnight in a non-CO2 incubator. Once calibrated, the cell plate replaced the assay plate, and OCR was measured simultaneously. The assay protocol involved sequential injection of four compounds to modulate mitochondrial function and determine parameters such as basal respiration, maximal respiration, and ATP production: oligomycin (1 μM), an ATP synthase inhibitor for maximal glycolytic metabolism; carbonyl cyanide p-(trifluoromethoxy) phenylhydrazone (FCCP) (1 μM), an ETC and OXPHOS uncoupler for peak oxygen consumption and oxidative metabolism; rotenone and antimycin A (both at 1 μM), inhibitors of ETC complexes I and III respectively, for non-mitochondrial respiration assessment. The Seahorse analyzer recorded OCR values throughout the assay to monitor cellular metabolic activity in real-time.

#### Restriction enzyme digestion assay

To confirm the successful ligation of inserts into the empty vector, a restriction enzyme digestion assay was performed. Each construct (1 μg) was incubated with EcoRI and NdeI restriction enzymes in FastCut Buffer at 37°C for 15 min. The digested constructs were then analyzed via agarose gel electrophoresis, with Loading STAR (DYNE Bio) used as the loading dye for visualization.

#### Cell culture and transfection

HEK293T cells were cultured and maintained in Dulbecco’s modified Eagle medium (DMEM) supplemented with 10% fetal bovine serum and penicillin (50 IU/mL)/streptomycin (50 μg/mL; Invitrogen), and were regularly tested for mycoplasma contamination with consistently negative results. The cells were transfected with WT or mutant KCNQ4 plasmids using Lipofectamine 2000 reagent (Invitrogen). Homotetrameric KCNQ4 channels, KCNQ4 wild-type (WT) and p.Arg390Cys (R390C) were cloned into the pRK5 vector and each plasmid (4.0 μg) was transiently expressed with pEGFPN-1 (0.4 μg, BD Biosciences) in HEK293T cells. HEK293T cells transfected with empty pRK5 vectors and green fluorescent protein (GFP) were used as a negative control group.

#### Immunocytochemistry

HEK293T cells were transfected with KCNQ4 WT and KCNQ4 R390C plasmids using Lipofectamine 3000 reagent (L3000008, Invitrogen, USA) and incubated in a humidified atmosphere with 5% CO_2_ at 37°C for 24 h. After incubation, the transfected cells were fixed with 4% paraformaldehyde for 15 min, followed by three washes with PBS. The fixed cells were then permeabilized with ice-cold 100% methanol for 10 min and washed three more times with PBS. Next, the cells were blocked with 2% BSA in PBS and incubated overnight at 4°C with an anti-Flag antibody (F3165, Sigma-Aldrich, USA; 1:100). After three washes with chilled PBS (4°C), the cells were incubated with an Alexa Fluor 488–conjugated secondary antibody (A32723, Invitrogen, USA; 1:1,000) at room temperature for 90 min. The plasma membrane was labeled using Alexa Fluor 633–conjugated concanavalin A (C21402, Invitrogen; 1:200) following the manufacturer’s guideline. Finally, the cells were mounted using a DAPI-containing mounting solution (ab104139, Abcam, USA). Fluorescent signals were primarily observed and captured using a confocal microscope (Leica Microsystems, STELLARIS 8).

#### Whole-cell patch clamp

The whole-cell patch clamp method for evaluating KCNQ4 channels was described in our previous publication.^89^ In brief, HEK293T cells were subcultured to 70–90% confluence and plated for transfection in 6-well culture dishes. KCNQ4 channel currents were recorded using the whole-cell patch-clamp technique. Patch pipettes, with a tip resistance of 1.5–3 MΩ, were pulled from borosilicate glass tubing (WPI, USA) and fire-polished with a microforge (MF-83, Narishige, Japan). The whole-cell currents were recorded with an Axopatch 200B amplifier (Molecular Devices, USA), filtered at 5 kHz, and sampled at 10 kHz. Series resistance was compensated, and membrane capacitance (C_m_) was measured and canceled before each recording. KCNQ4 K^+^ currents were elicited using 2-s depolarizing voltage steps (−70 to +40 mV, 10-mV increments), followed by a 1-s hyperpolarizing step to −50 mV. Current amplitudes at +40 mV were normalized to C_m_ and expressed as current densities (pA/pF). Steady-state activation curves were generated from tail current amplitudes, plotted against prepulse voltages, and fitted with the Boltzmann function to calculate half-activation voltages (V_0.5_). The V_0.5_ values of the GFP-transfected control cells were obtained from current-voltage (I-V) curves by calculating the normalized conductance (G/G_max_). Recordings were performed at room temperature (∼23°C) and analyzed with Clampex software (pCLAMP 10, Molecular Devices, USA). The external bath solution used for whole-cell patch-clamp recordings consisted of 147 mM NaCl, 5 mM KCl, 1.5 mM CaCl_2_, 1 mM MgCl_2_, 10 mM HEPES, and 10 mM D-glucose, with the pH adjusted to 7.4 using N-methyl-D-glucamine (NMDG). The internal patch pipette solution contained 130 mM KCl, 10 mM NaCl, 10 mM EGTA, 10 mM HEPES, 3 mM Mg-ATP, and 0.5 mM CaCl_2_, with the pH adjusted to 7.2 using KOH. The free Ca^2+^ concentration was calculated to be approximately 10 nM.

#### Comparative analysis of gene expression patterns

Publicly available transcriptome data from human inner ear organoids and human cochlear and vestibular organs (GSE213796) were used to compare gene expression patterns. Correlation trends within predefined gene categories were assessed by computing pairwise gene expression correlations using Spearman’s rank correlation coefficient. For each gene group, the mean correlation coefficient was calculated, excluding self-comparisons and redundant pairs. To evaluate statistical significance, a permutation test with 1,000 iterations was performed. In each iteration, gene labels were randomly reassigned while maintaining the original group sizes, and correlation coefficients were recalculated. The observed summary statistic (weighted mean of within-group correlations) was compared to the null distribution derived from the permutations to compute a *p* value. Groups with fewer than three genes were excluded from the analysis.

#### Statistical analyses

We used the Pearson chi-square test to identify variables that could potentially differentiate between the genetically diagnosed and undiagnosed groups. Following the computation of the OR and 95% CI for each value, we conducted a logistic regression analysis, considering only the variables with *p* values of <0.05. This approach facilitated the derivation of the adjusted OR. We also classified the 63 SNHL-associated genes that were identified during this study based on the molecular mechanisms of inner-ear function. To verify the variance between groups according to clinical phenotypes, we used the Pearson chi-square test. Finally, we used Fisher’s exact test based on the false discovery rate (FDR) to identify statistically significant groups characterized by an adjusted *p* value of <0.05. In this study, we define variables exhibiting *p* values of <0.05 as statistically significant.

## Supplemental information

Document S1. Figures S1–S20 and Tables S1, S3–S5, S7, S9, and S10

Table S2. Whole list of subjects and diagnosed genetic variants

Table S6. Genomic variants identified from patients with SNHL

Table S8. List of pathogenic and high-impact rare variants identified in undiagnosed patients

Data S1. Informed consent form for patient with SNHL

Document S2. Article plus supplemental information
