# Supplementary material for: Comprehensive genetic profiling of sensorineural hearing loss using an integrative diagnostic approach
Source: Cell Rep Med. 2025 Jun 30;6(7):102206. doi: 10.1016/j.xcrm.2025.102206 (PMC12281402; doi:10.1016/j.xcrm.2025.102206)
Supplement: Document S1. Figures S1–S20 and Tables S1, S3–S5, S7, S9, and S10 [file mmc1.pdf]

Cell Reports Medicine, Volume *6*

Supplemental information

Comprehensive genetic profiling of sensorineural hearing loss using an integrative diagnostic approach

Sang-Yeon Lee, Seungbok Lee, Seongyeol Park, Sung Ho Jung, Yejin Yun, Won Hoon Choi, Ju Hyuen Cha, Hongseok Yun, Sangmoon Lee, Myung-Whan Suh, Moo Kyun Park, Jae-Jin Song, Byung Yoon Choi, Jun Ho Lee, Tong Mook Kang, Young Seok Ju, June-Young Koh, and Jong-Hee Chae

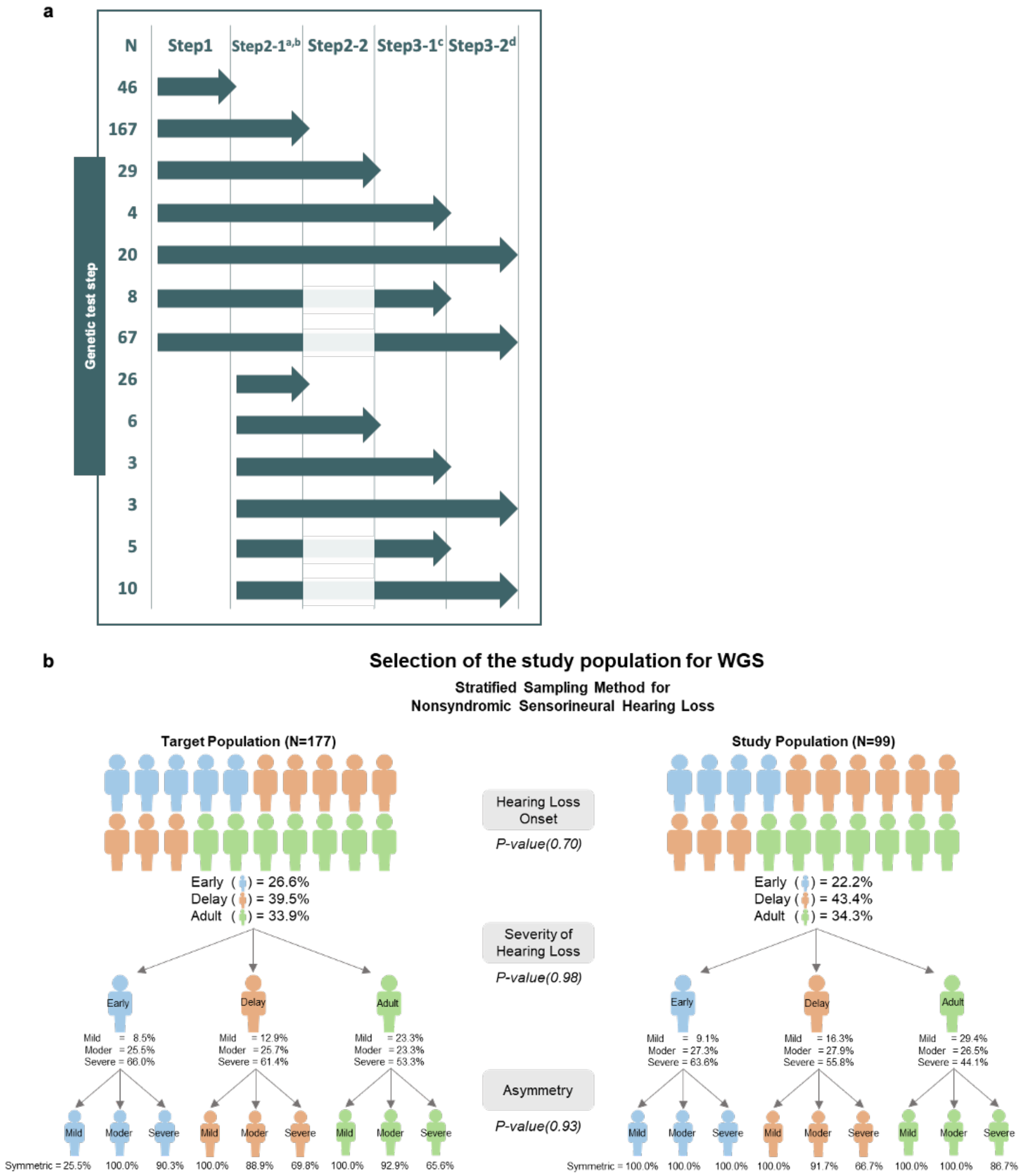

Figure S1. Stepwise genomic approach for the genetic diagnosis of SNHL patients.

(a) Numbers of patients for each genetic test step. (b) Selection of the study population for WGS (Step 3-1). Among patients who remained undiagnosed, all s-SNHL patients and a representative subset of ns-SNHL patients were selected for WGS using a well-thought-out sample size estimation with a stratified sampling approach.

[a] For patients with non-syndromic sensorineural hearing loss (ns-SNHL) who underwent Step 2-1, we initially recommended the TPS approach, which includes 246 hearing loss–related genes established in our center. However, ns-SNHL patients who decline TPS are referred to the WES program supported by a rare disease project. Conversely, all patients with syndromic sensorineural hearing loss (s-SNHL) are referred directly to the WES program. [b] Ten VUS identified in Step 2-1, including *TMPRSS3* c.743C>T (p.Thr248Met), *ESRRB* c.1144C>T (p.Arg382Cys), *SIX1* c.501G>C (p.Gln167His), *LMX1A* c.719A>G (p.Gln240Arg), *LMX1A* c.721G>A (p.Val241Met), *KCNQ4* c.1168C>T (p.Arg390Cys), *TMC1* c.1256T>C (p.Phe419Ser), *TMC1* c.1444T>C (p.Trp482Arg), *DSPP* c.51+5G>A (p.?), and *ELMOD3* c.640G>A (p.Gly214Ser), were reclassified as likely pathogenic or pathogenic based on functional assay results in accordance with the ACMG guidelines, and subsequently included in the final diagnostic list (**Table S7**). [c] Three structure variants identified in Step 3-1, including *EYA1* g.[71211857_71228236inv;71211857_71215145del], *CLCNKA_CLCNKB* c.1804_ [NC_000001.11:g.16046349]del, and *SPATA5* c.2227-3015_2354+1415del, were reclassified as likely pathogenic or pathogenic based on functional assay results and included in the final diagnostic list (**Table S7**). [d] Three deep intronic variants in *USH2A* (c.7120+1475A>G, c.14134-3169A>G, c.4628-26037A>G), originally identified in Step 3-2, were reclassified as likely pathogenic based on functional assay results in accordance with the ACMG guidelines for hearing loss, and subsequently included in the final diagnostic list (**Table S7**).

Abbreviations: SNHL, sensorineural hearing loss; VUS, variants of uncertain significance; BOR/BO syndrome, branchio-oto-renal/branchio-otic syndrome; MLPA, multiplex ligation-dependent probe amplification; mtDNA, mitochondrial DNA; CI, confidence interval.

a

1. GJB2(NM_004004.5)	2. SLC26A4(NM_000441.1)	3. OTOF(NM_194248.2)	4. TMC1(NM_138691.2)	5. COCH(NM_004086.2)
c.109G>A;p.Val37Ile	c.1229C>T; p.Thr410Met	c.2521G>A;p.Glu841Lys	c.1714G>A;p.Ast572Asn	c.113G>A;p.Gly38Asp
c.299_300delAT;p.His100ArgfsTer4	c.2027T>A; p.Leu676Gln	c.5566C>T;p.Arg1856Trp	c.100C>T;p.Arg34Ter	c.485G>A;p.Cys162Tyr
c.235del;p.Leu79CysfsTer3	c.2168A>G;p.His723Arg	c.3032T>C;p.Leu1011Pro		
c.427C>T;p.Arg143Trp	c.919-2A>G;p.?	c.3192C>G;p.Tyr1064Ter		
		c.5816G>A;p.Arg1939Gln		
6. CDH23(NM_022124.5)	7. TMPRSS3(NM_024022.2)	8. MT-RNR1(NC_012920.1)	9. ATP1A3(NM_152296.4)	10. MPZL2(NM_005797.3)
c.719C>T;p.Pro240Leu	c.916G>A;p.Ala306Thr	m.1555A>G	c.2452G>A;p.Glu818Lys	c.220C>T;p.Gln74Ter

b

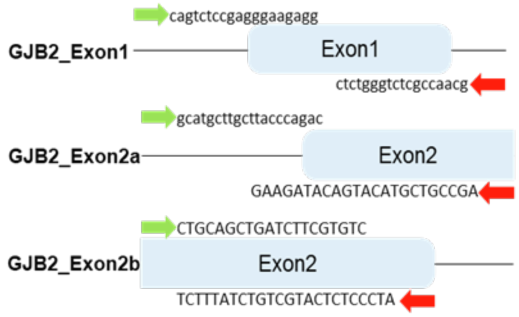

c

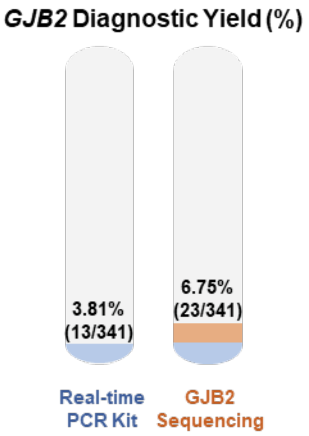

Real-time PCR Kit

Variant1	Variant2	N
c.109G>A;p.Valr37Ile	c.109G>A;p.Valr37Ile	1
c.109G>A;p.Val37Ile	c.427C>T;p.Arg143Trp	4
c.109G>A;p.Valr37Ile	c.235del;p.Leu79CysfsTer3	1
c.235del;p.Leu79CysfsTer3	c.235del;p.Leu79CysfsTer3	5
c.235del;p.Leu79CysfsTer3	c.427C>T;p.Arg143Trp	1
c.235del;p.Leu79CysfsTer3	c.299_300del;p.His100ArgfsTer4	1

GJB2 single-gene sequencing

Variant1	Variant2	N
c.235del;p.Leu79CysfsTer3	c.257C>G;p.Thr86Arg	2
c.235del;p.Leu79CysfsTer3	c.176_191del;p.Gly59AlafsTer18	2
c.235del;p.Leu79CysfsTer3	c.560_605dup;p.Cys202Ter	1
c.257C>G;p.Thr86Arg	c.299_300del;p.His100ArgfsTer4	2
c.109G>A;p.Valr37Ile	c.416G>A;p.Ser139Asn	1
c.176_191del;p.Gly59AlafsTer18	c.176_191del;p.Gly59AlafsTer18	1
c.109G>A;p.Valr37Ile	c.176_191del;p.Gly59AlafsTer18	1

d

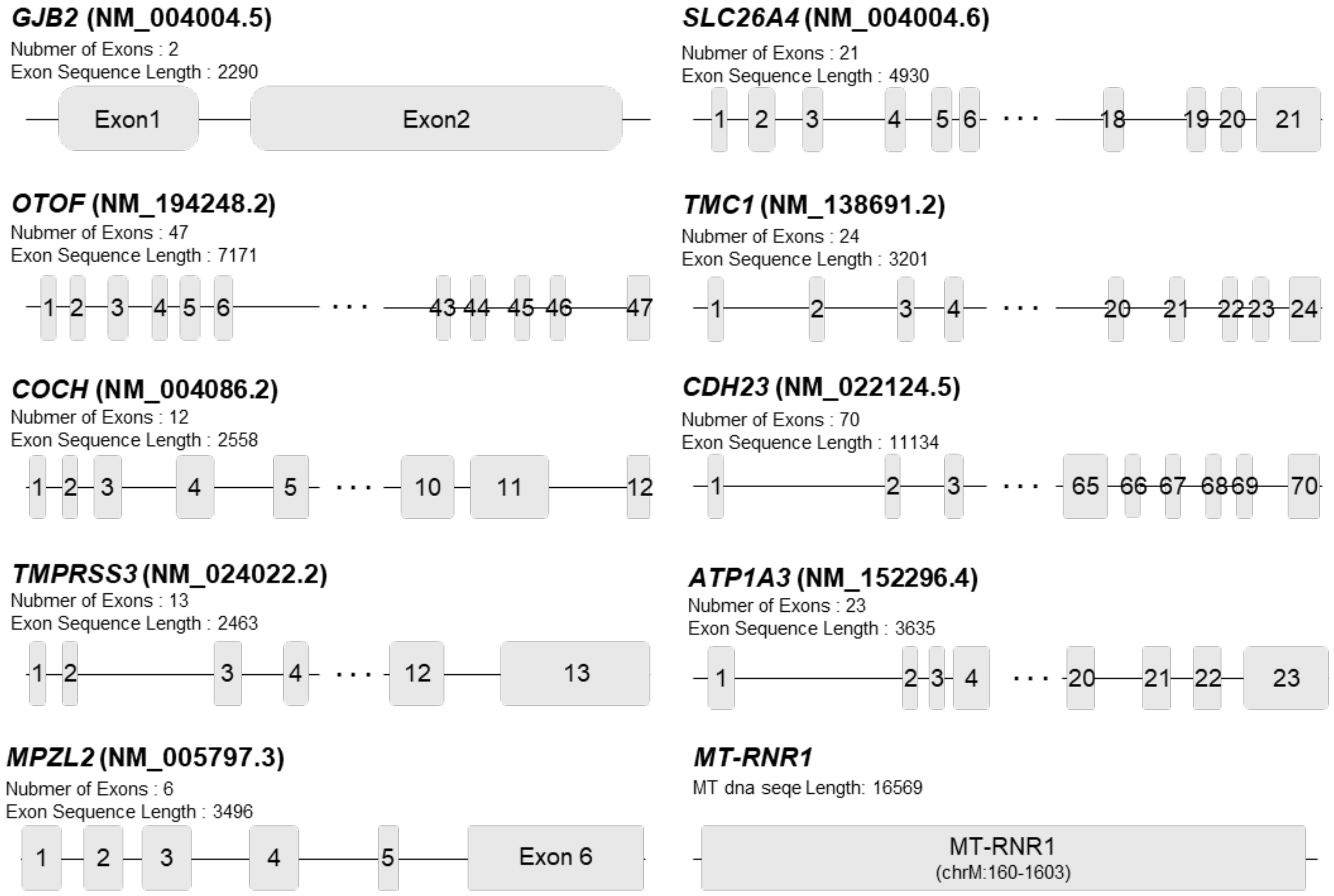

Figure S2. Detailed information of Step 1.

(a) Overview of 22 variants from 10 classical deafness genes (*GJB2, SLC26A4, TMPRSS3, CDH23, OTOF, TMC1, ATP1A3, MPZL2, COCH, and 12S rRNA*) included in the real-time PCR screening kit. (b) *GJB2* diagnostic yield in ns-SNHL patients using (i) the real-time PCR screening kit alone and (ii) the real-time PCR screening kit combined with GJB2 single-gene sequencing, along with detailed genotypes. (c) Primer sets used for *GJB2* single-gene sequencing. (d) Number of exons and total exon sequence length of the 10 classical deafness genes in the real-time PCR screening kit. Abbreviation: ns-SNHL, non-syndromic sensorineural hearing loss.

a

ACTB, ACTG1, ADCY1, ADGRV1, AIFM1, ALMS1, ANOS1, AP2M1, ATP1A2, ATP1A3, ATP6V1B1, ATP6V1C2, BCS1L, BDP1, BSND, BTD, CABP2, CACNA1D, CAPZA1, CAPZA2, CAPZB, CATSPER2, CCBE1, CCDC50, CD164, CDH23, CEACAM16, CEMIP, CEP78, CFL1, CHD7, CIB2, CISD2, CLDN14, CLDN9, CLIC5, CLPP, CLRN1, CLRN2, COCH, COL11A1, COL11A2, COL1A1, COL2A1, COL4A3, COL4A4, COL4A5, COL4A6, COL9A1, COL9A2, COL9A3, CRYM, CSPP1, DCAF17, DIABLO, DIAPH1, DIAPH3, DLX5, DMXL2, DSPP, DSTN, ECE1, EDN3, EDNRA, EDNRB, EEF1AKNMT, EFTUD2, ELMOD3, EPG5, EPS8, EPS8L2, ERAL1, ERCC2, ERCC3, ESPN, ESRRB, EYA1, EYA4, FANCA, FANCD2, FAS, FDXR, FGF3, FGFR1, FGFR2, FGFR3, FKBP14, FRAS1, FUCA1, GALE, GATA3, GIPC3, GJA1, GJB1, GJB2, GJB3, GJB4, GJC3, GPSM2, GRAP, GREB1L, GRHL2, GRXCR1, GRXCR2, GSDME, GSTP1, HAAO, HAL, HGF, HOMER2, HOXA2, HSD17B4, IFNLR1, ILDR1, JAG1, KARS1, KCNE1, KCNJ10, KCNK3, KCNQ1, KCNQ4, KIT, KITLG, LARS2, LHFPL5, LHX3, LMX1A, LOXHD1, LOXL2, LOXL3, LRP2, MAN2B1, MANBA, MARVELD2, MASP1, MCM2, MET, MFN2, MGP, MITF, MPZL2, MSRB3, MYH1, MYH14, MYH9, MYO15A, MYO1A, MYO1C, MYO1F, MYO3A, MYO6, MYO7A, NDP, NF2, NLRP12, NLRP3, OPA1, ORC1, OSBPL2, OTOA, OTOF, OTOG, OTOGL, OTOP1, OTOR, P2RX2, PAX2, PAX3, PCDH15, PDE1C, PDZD7, PEX1, PEX2, PEX6, PEX7, PIGV, PJVK, PLS1, PMP22, PNPT1, POU3F4, POU4F3, PRPS1, PTPN11, PTPRQ, RDX, REST, RIPOR2, RNF135, ROR1, RPS6KA3, S1PR2, SALL1, SALL4, SCD5, SCN9A, SEMA3E, SEMA3F, SERAC1, SERPINB6, SERPINF1, SGPL1, SIX1, SIX5, SLC12A2, SLC12A7, SLC17A8, SLC22A4, SLC26A2, SLC26A4, SLC26A5, SLC29A3, SLC4A11, SLITRK6, SMARCA4, SMPX, SNAI2, SOX10, SOX2, SPATA5, SPINK5, SPNS2, SUCLG1, SYNE4, STRC, TBC1D24, TBL1X, TBX1, TECTA, TECTB, TFAP2A, TFCP2, TH, TIMM8A, TJP2, TMC1, TMIE, TMPRSS3, TNC, TPRN, TRIOBP, TWNK, USH1G, USH2A, WFS1, WHRN

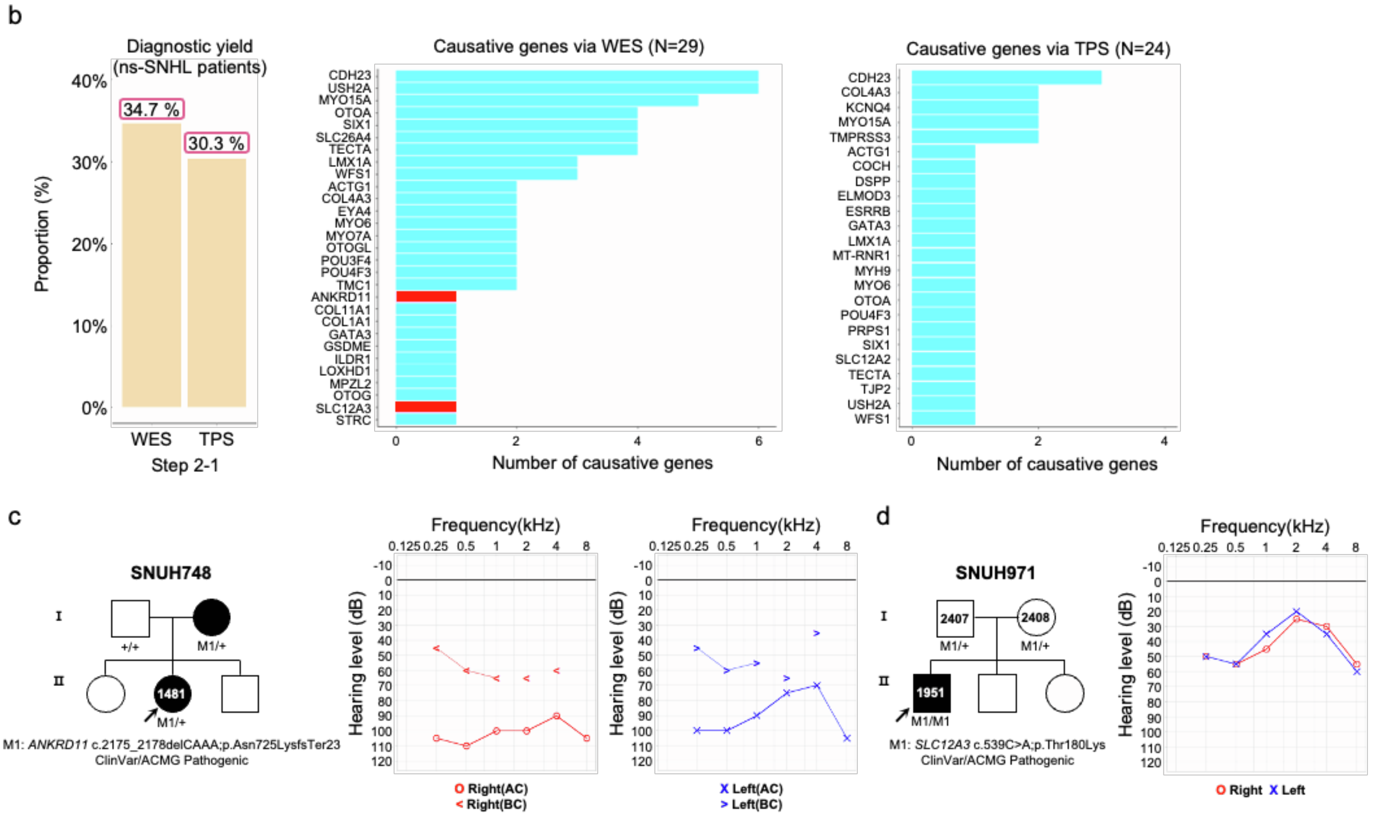

Figure S3. Diagnostic yield and gene profiles between TPS and WES in Step 2-1.

(a) List of 246 SNHL-related genes analyzed by TPS. (b) Comparison of diagnostic yield and gene profiles between TPS and WES in ns-SNHL patients. Two families (*ANKRD11* and *SLC12A3*) were identified via WES but were not included in the TPS panel. (c) Pedigrees, genotypes, and auditory phenotypes of two families segregating *ANKRD11* p.Asn725LysfsTer23 (SNUH 748) and *SLC12A3* p.Thr180Lys (SNUH 971). The arrow indicates the proband, and black-filled symbols represent affected individuals. Abbreviations: ns-SNHL, non-syndromic sensorineural hearing loss; TPS, targeted panel sequencing; WES, whole-exome sequencing.

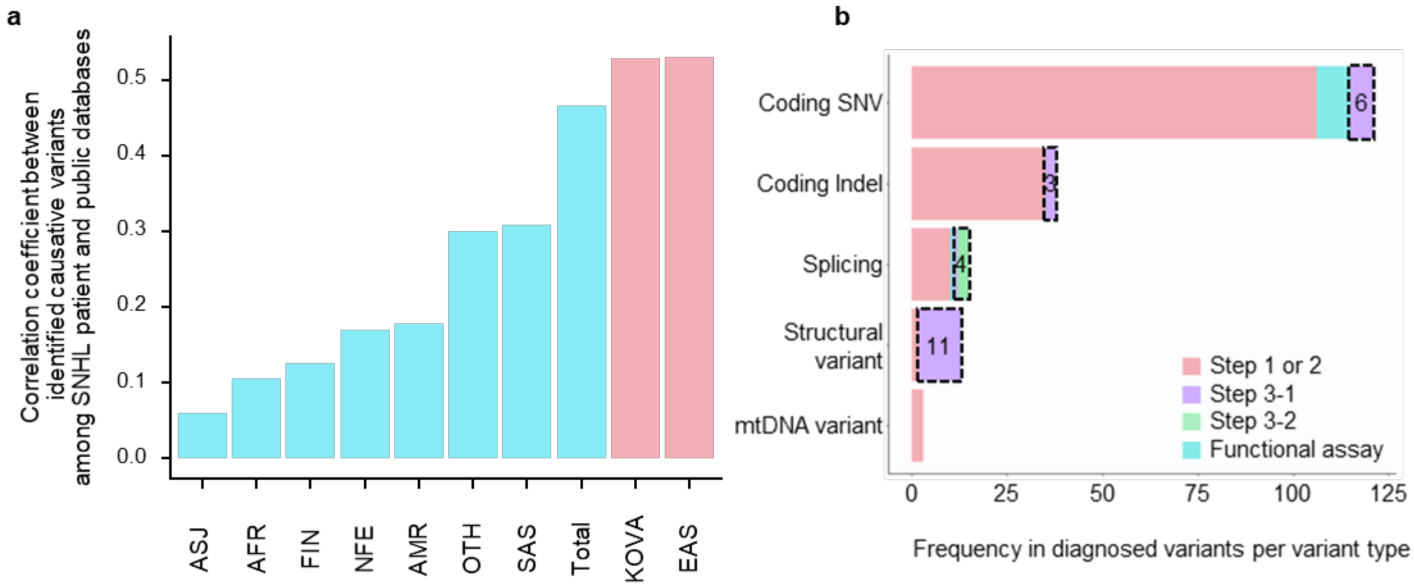

Figure S4. Detailed information on diagnostic outcomes in SNHL patients

(a) Spearman's correlation coefficient values for causative variants between the allele frequencies (AFs) in our cohort and those from other populations. (b) Distribution of variant subtypes identified at each diagnostic step. Duplicated variants were excluded in this figure, and visualization was based on the count of unique variants. SNHL, sensorineural hearing loss; mtDNA, mitochondrial DNA.

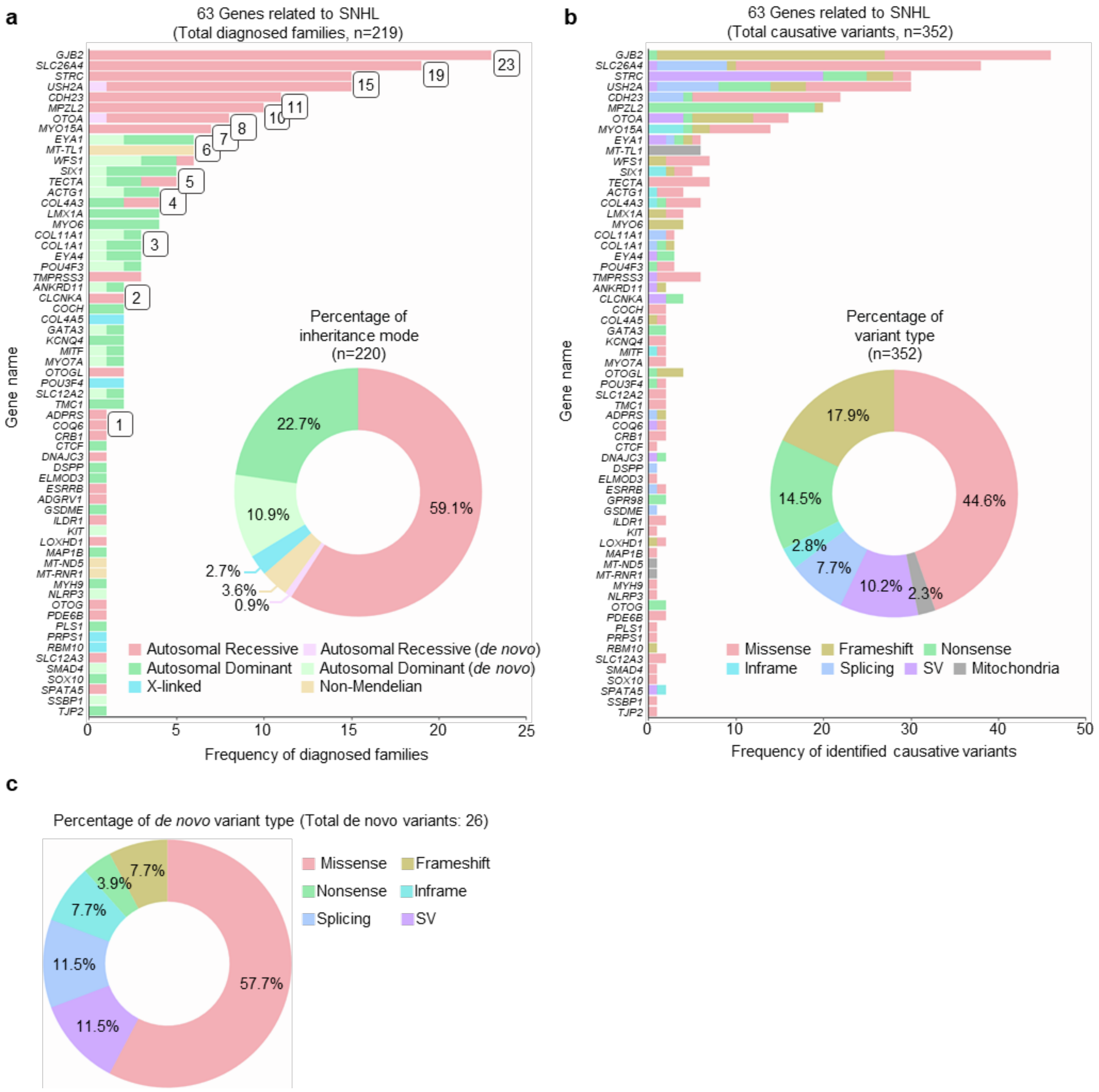

Figure S5. Distribution of likely pathogenic or pathogenic variants identified in our SNHL cohort.

(a) Bar plot showing the frequencies and inheritance patterns of 63 SNHL-associated genes from 219 genetically diagnosed families. Pie chart showing the percentages of inheritance patterns. (b) Bar plot showing the mutational landscape of the total 352 likely pathogenic or pathogenic variants among the 63 SNHL genes. Pie chart showing the percentages of variant types. (c) Pie chart showing the percentage distribution of variant types among de novo variants.

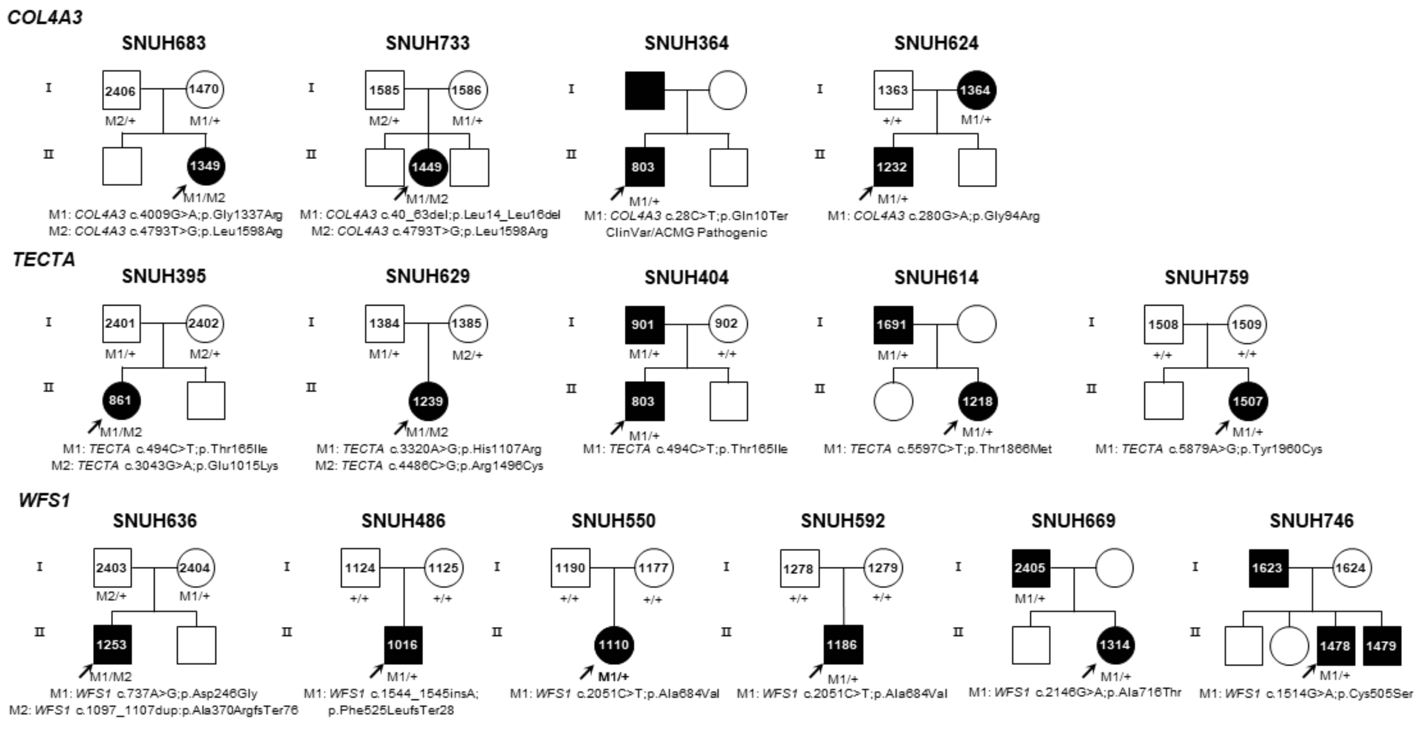

Figure S6. Pedigrees of families carrying *COL4A3*, *TECTA*, and *WFS1* variants.

Inheritance patterns of 15 unrelated families carrying *COL4A3*, *TECTA*, and *WFS1* variants are illustrated, with arrows indicating the probands. It is well established that the *TECTA* (DFNA8/12 and DFNB21), *COL4A3* (ATS3A and ATS3B), and *WFS1* (DFNA6/14/38 and Wolfram syndrome) can cause disease through both autosomal dominant (AD) and autosomal recessive (AR) inheritance.

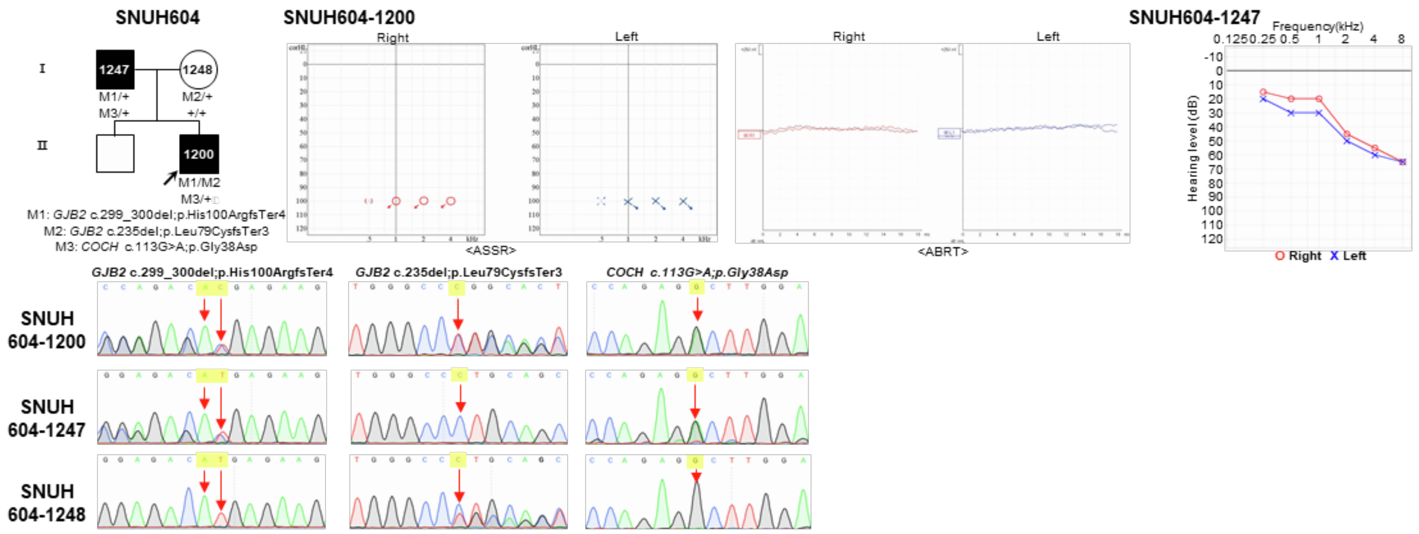

Figure S7. SNUH604 family with a dual primary genetic etiology.

Illustration of one family (SNUH604) with a dual primary genetic etiology, segregating *GJB2* compound heterozygous variants and a *COCH* heterozygous variant. These variants have been previously reported as pathogenic according to the ClinVar database and the ACMG-AMP guidelines.

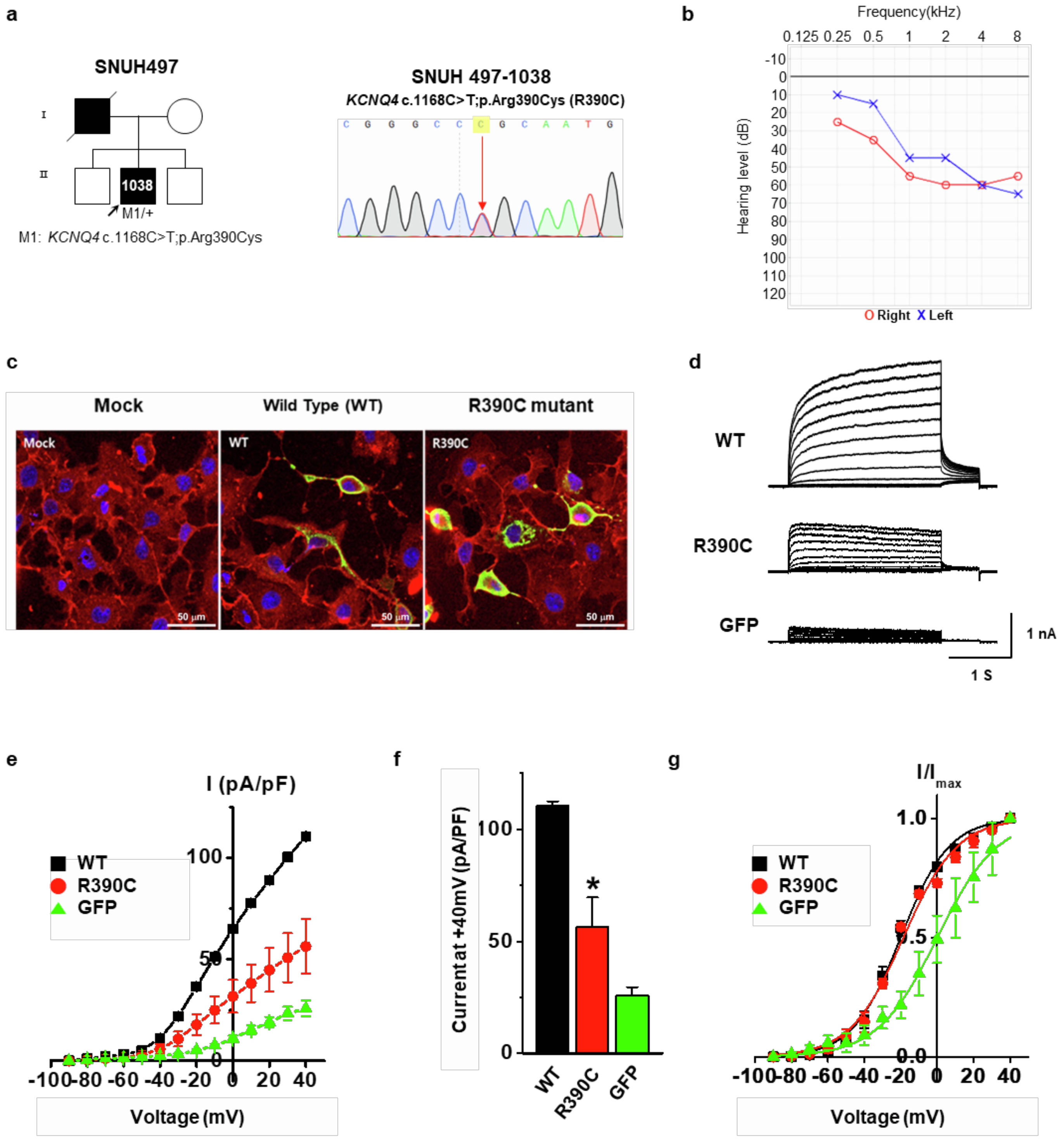

Figure S8. Functional pathogenicity of *KCNQ4* p.Arg390Cys variant.

(a) Pedigree of family SNUH497 and the corresponding Sanger sequencing chromatogram revealing segregation of the *KCNQ4* p.Arg390Cys variant (R390C). (b) Pure-tone audiogram of the proband (SNUH497-1038) showing a symmetric, high-frequency, down-sloping sensorineural hearing loss. (c) Immunofluorescence images of HEK293T cells transiently transfected with an N-terminally FLAG-tagged KCNQ4 wild-type (WT) or R390C mutant construct. Cells were co-stained with anti-FLAG (green) and anti-Concanavalin A (red) antibodies. Both WT and R390C channels are localized to the plasma membrane. Scale bar, 50um. (d) Whole-cell K^+ currents were recorded using the whole-cell patch clamp technique in HEK293T cells transiently expressing KCNQ4 WT or the R390C mutant. (e) Current-voltage (I–V) relationships of the recorded currents, with the comparison of maximum current amplitudes at +40 mV. (f) Current density at +40 mV in cells expressing KCNQ4 WT or R390C mutant. The mutant channel exhibited a current amplitude of 56.3 ± 13.3 pA/pF (n=30), approximately 51% of the WT channel amplitude (110 ± 2.1 pA/pF, n=30). The current of

the GFP-expressing cells was 25 ± 2.0 pA/pF (n=10). (g) Steady-state activation curves yielded half activation voltages ($V_{0.5}$) of −20 ± 0.7 mV for WT (n=30), −18.5 ± 1.0 mV for R390C mutant (n=30), and 0.0 ± 0.9 mV for GFP (n=10). Data are presented as mean ± standard error of the mean (SEM). Statistical significance was determined by one-way ANOVA with Bonferroni post-hoc analysis (*, $P < 0.05$). These results suggest that the *KCNQ4* p.R390C variant is a hypomorphic allele with partial loss-of-function channel activity.

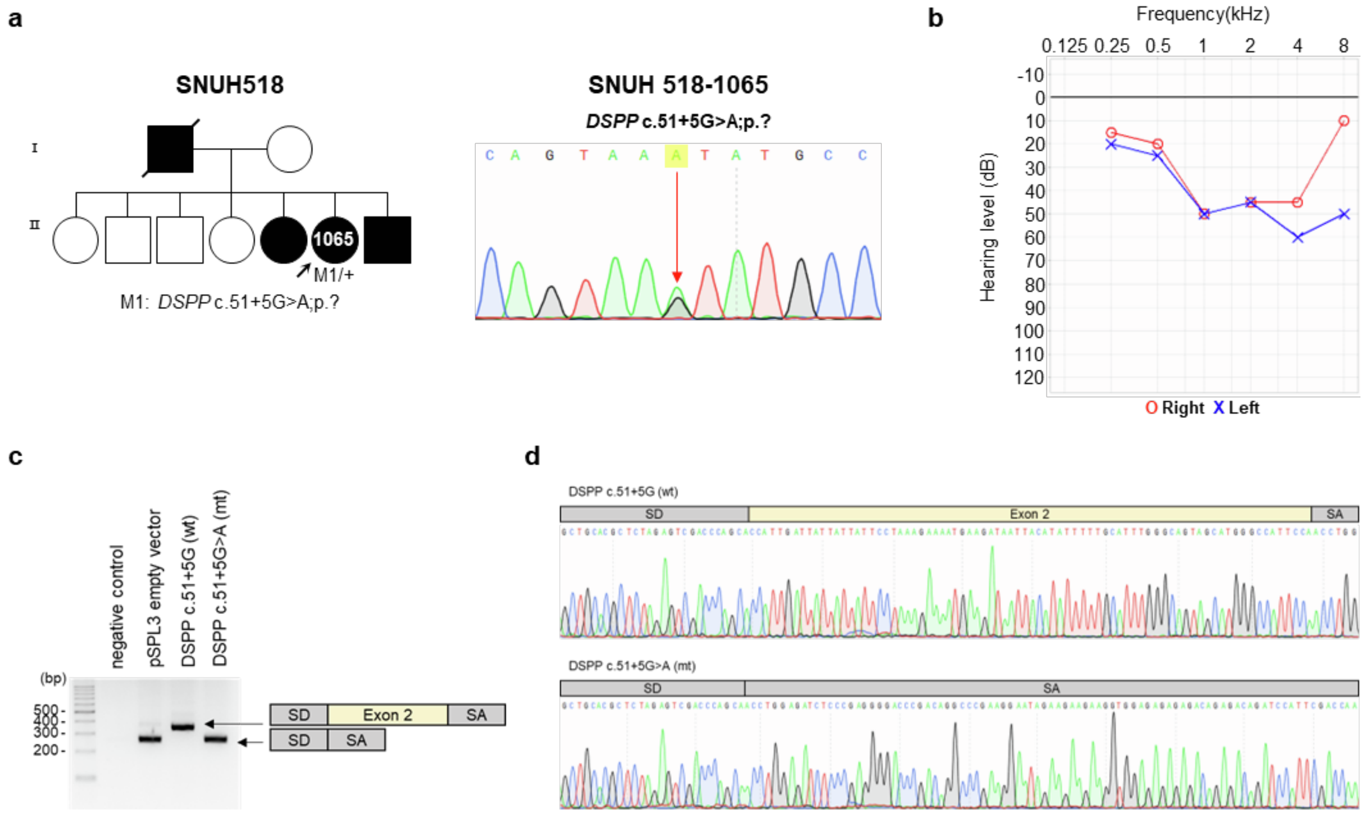

Figure S9. Functional pathogenicity of *DSPP* c.51+5G>A variant.

(a) Pedigree of family SNUH518 and the corresponding Sanger sequencing chromatogram revealing segregation of the *DSPP* c.51+5G>A variant. (b) Pure-tone audiogram of the proband (SNUH 518-1065) showing a symmetric, high-frequency sensorineural hearing loss. (c) HEK293T cells were transfected with either the wild-type *DSPP* minigene, the c.51+5G>A variant *DSPP* minigene, or an empty pSPL3 vector. Following RNA extraction and cDNA synthesis, PCR was performed using SD6 and SA2 vector-specific primers to amplify splicing products, which were then analyzed via agarose gel electrophoresis. The wild-type *DSPP* minigene produced a 339 bp splicing product, whereas the c.51+5G>A variant resulted in exon skipping, yielding a 260 bp product. (d) The DNA band identified in minigene splicing assay was subjected to Sanger sequencing, which confirmed exon 2 skipping in the c.51+5G>A variant *DSPP* minigene.

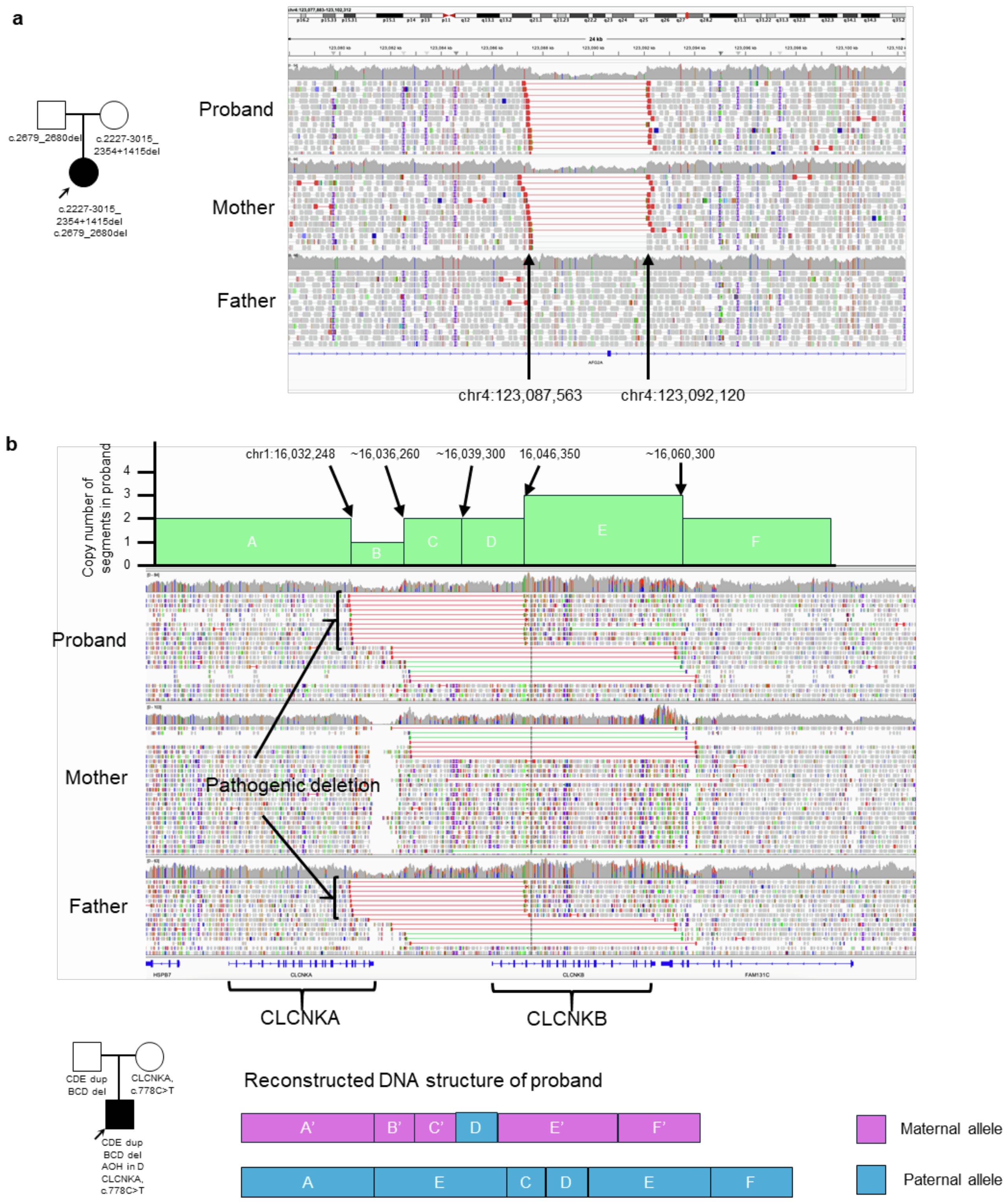

Figure S10. Detailed information of the *SPATA5* and *CLCNKA* deletions.

(a) Pedigree and screenshot of *SPATA5* deletion. (b) Pedigree, screenshot, and schematic illustration of *CLCNKA* deletion. The copy number and assigned ID of segments are illustrated in the top row. The allele-specific DNA structure was reconstructed using read depth and SNP information (bottom row). The proband received the CDE duplication and BCD deletion allele from his father, resulting in the truncation of *CLCNKA*. We also observed a uniparental disomy event in segment D, leading to an absence of heterozygosity in that segment.

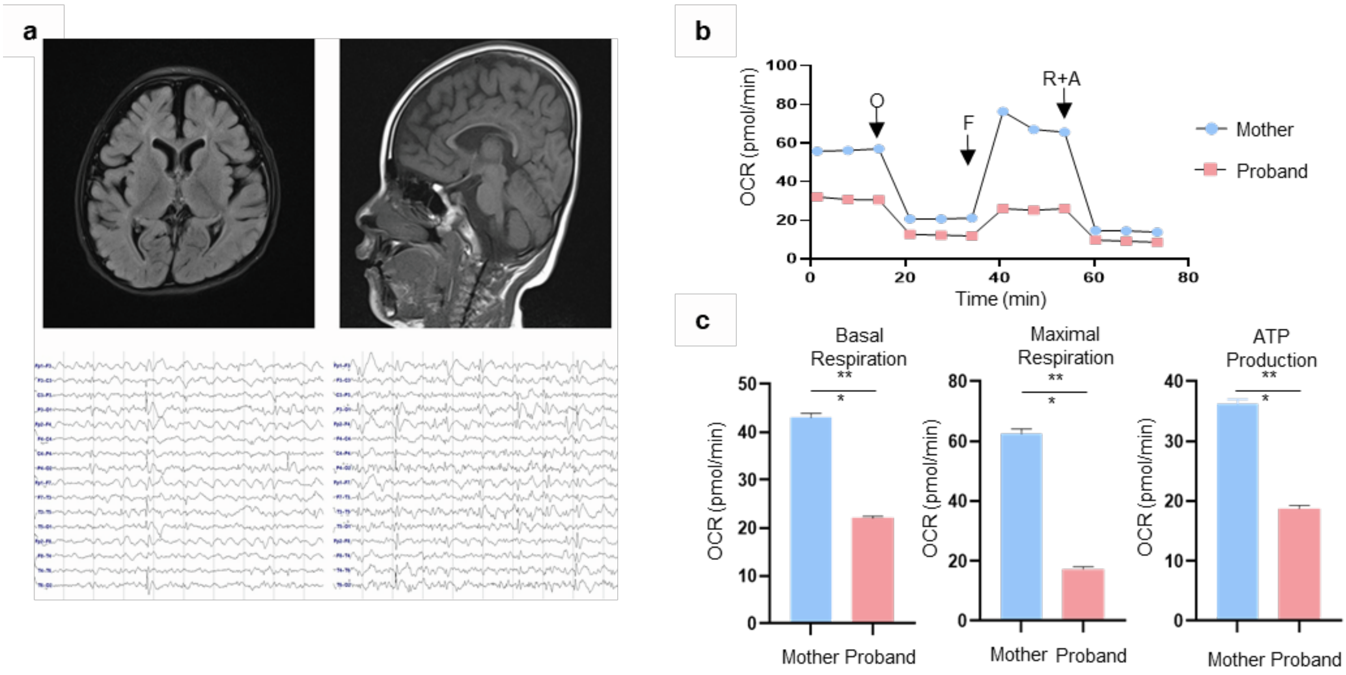

Figure S11. Phenotypic and molecular presentations of *SPATA5* mutations

(a) Brain MRI of the patient with *SPATA5* mutations (SNUH 799), revealing diffuse brain atrophy (T2-FLAIR axial image, top left) and diffuse thinning of the corpus callosum (T1 sagittal image, top right). The EEG shows frequent multifocal spike or spike-wave discharges (bottom). (b) Oxygen consumption rate (OCR) in fibroblast cells were measured under basal conditions and after injection of oligomycin (O), carbonyl cyanide 4-(trifluoromethoxy) phenylhydrazone (FCCP; F), rotenone (R) and antimycin A (AA). (c) Changes in basal respiration, maximal respiration, and ATP production in fibroblast cells were compared from OCR traces; ***, $p < 0.001$ (mean ± SEM, n = 5, unpaired Student's t test).

a

GJB2, GJB6, MYO7A, MYO15A, SLC26A4, TMIE, TMC1, TMPRSS3, OTOF, CDH23, GIPC3, STRC, USH1C, OTOG, TECTA, OTOA, PCDH15, RDX, GRXCR1, GAB1, TRIOBP, CLDN14, MYO3A, WHRN, CDC14A, ESRRB, ESPN, MYO6, HGF, ILDR1, ADCY1, CIB2, MARVELD2, BDP1, COL11A2, PDZD7, PJVK, SLC22A4, SLC26A5, LRTOMT, COMT2, DCDC2, LHFPL5, S1PR2, PNPT1, BSND, MSRB3, SYNE4, LOXHD1, TPRN, GPSM2, PTPRQ, OTOGL, TBC1D24, ELMOD3, KARS1, SERPINB6, CABP2, NARS2, MET, TSPEAR, TMEM132E, PPIP5K2, GRXCR2, EPS8, CLIC5, RIPOR2, FAM65B, EPS8L2, WBP2, ROR1, ESRP1, COCH, MPZL2, CEACAM16, GRAP, SPNS2, CLDN9, CLRN2, GDF6, AFG2B, MINAR2, GPR156, TMTC4, STX4, PKHD1L1, GAS2, CEMIP, USH1G, LRP5, GOSR2, CEP250, FOXI1, KCNJ10, USH2A, ADGRV1, CLRN1, EDNRB, PAX3, EDN3, KNCQ1, KCNE1, COL4A4, COL4A3, TWNK, LARS2, HARS2, HSD17B4, COL9A1, COL9A2, COL9A3, POLR1C, CLCNKA, CASP10, PDE6B, CRB1, WFS1, ADPRS, GPR98, SPATA5, SLC12A3, DNAJC3, COQ6

b

Gene symbol	Variant information	Δ Score	Δtype
USH2A	NM_206933.4:c.4628-26037A>G	0.96	Acceptor gain
USH2A	NM_206933.4:c.7120+1475A>G	0.28	Acceptor gain
USH2A	NM_206933.4:c.14134-3169A>G	0.38	Donor gain

c

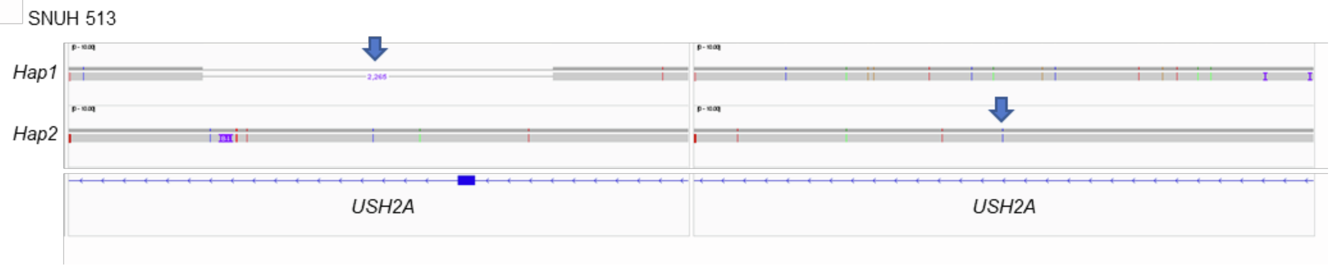

Figure S12. Carrier frequency analysis and identification of deep intronic variants from Step 3-2

(a) List of investigated SNHL-related genes with autosomal recessive (AR) mode of inheritance (MOI). (b) Table of identified candidate pathogenic variants located in deep intronic regions. (c) IGV visualization of long-read sequencing showing in trans status of identified pathogenic variants in SNUH 513.

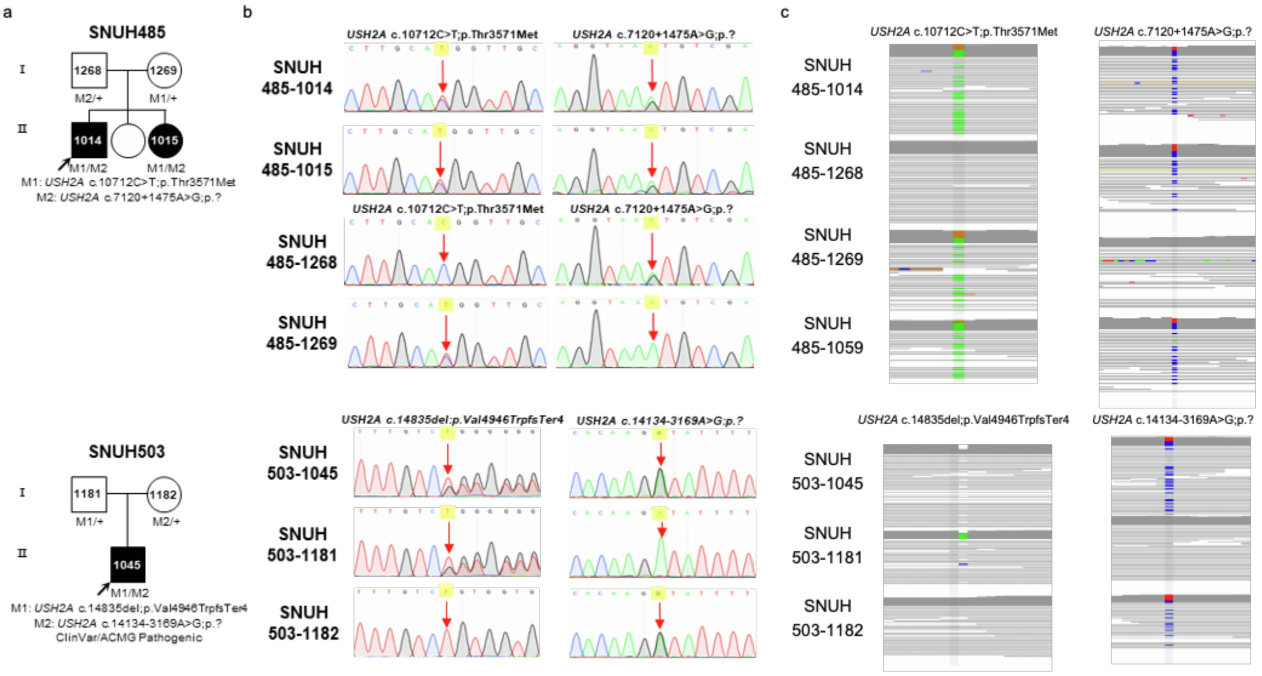

Figure S13. Pedigrees, Sanger sequencing chromatograms, and IGV snapshots of the SNUH485 and SNUH503 families.

(a) Pedigrees, (b) Sanger sequencing chromatograms, and (c) IGV sanpshots demonstrating the *in trans* configuration of *USH2A* compound heterozygous variants in the SNUH485 and SNUH503 families.

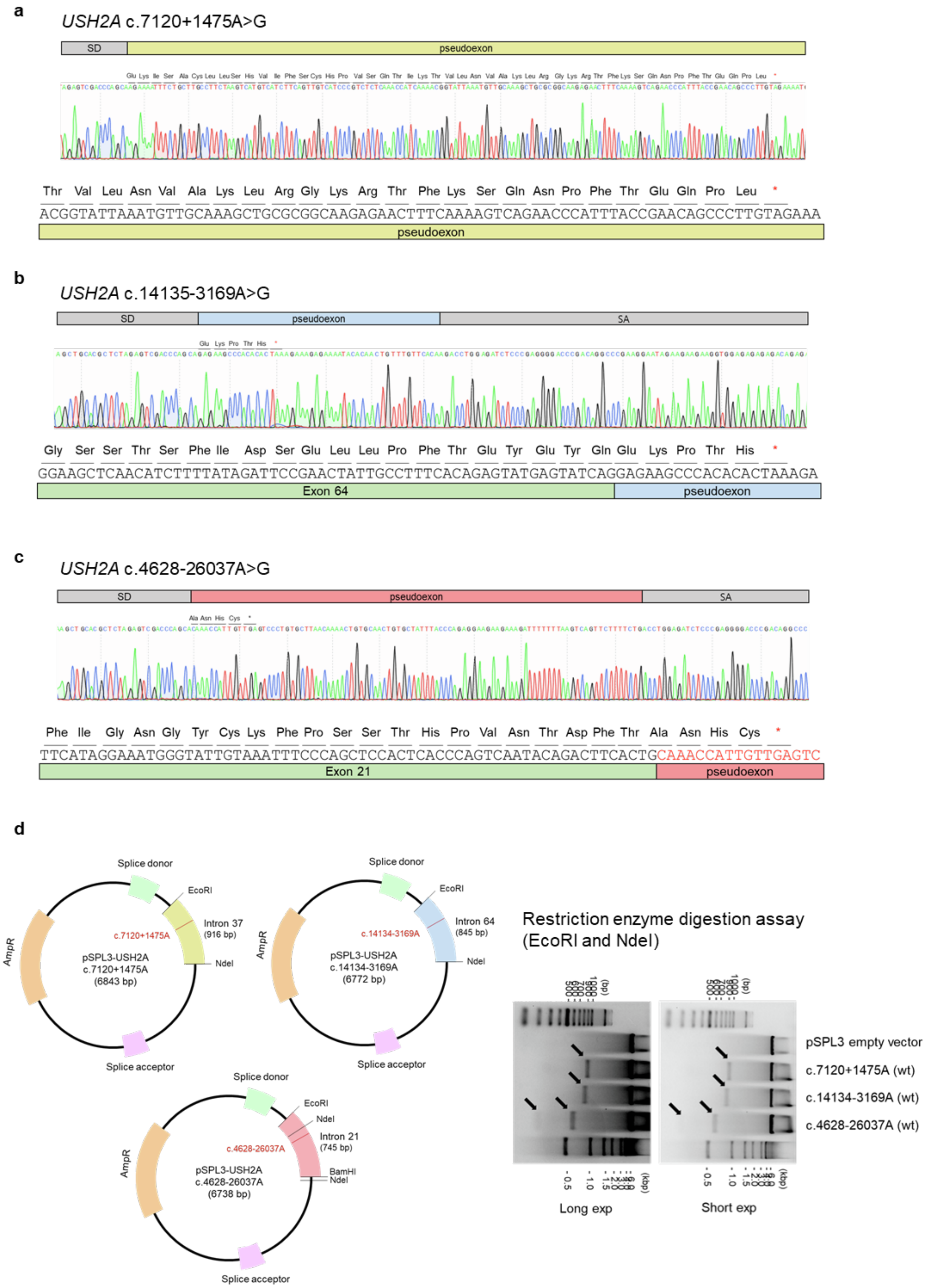
a
USH2A c.7120+1475A>G
SD
pseudoexon
Thr Val Leu Asn Val Ala Lys Leu Arg Gly Lys Arg Thr Phe Lys Ser Gln Asn Pro Phe Thr Glu Gln Pro Leu *
ACGGTATTAAATGTTGCAAAGCTGCGCGGCAAGAGAACTTTCAAAAGTCAGAACCCATTTACCGAACAGCCCTTGTAGAAA
pseudoexon
b
USH2A c.14135-3169A>G
SD
pseudoexon
SA
Gly Ser Ser Thr Ser Phe Ile Asp Ser Glu Leu Leu Pro Phe Thr Glu Tyr Glu Tyr Gln Glu Lys Pro Thr His *
GGAAGCTCAACATCTTTTATAGATTCCGAACTATTGCCTTTCACAGAGTATGAGTATCAGGAGAAGCCCACACACTAAAGA
Exon 64
pseudoexon
c
USH2A c.4628-26037A>G
SD
pseudoexon
SA
Phe Ile Gly Asn Gly Tyr Cys Lys Phe Pro Ser Ser Thr His Pro Val Asn Thr Asp Phe Thr Ala Asn His Cys *
TTCATAGGAAATGGGTATTGTAAATTTCCCAGCTCCACTCACCCAGTCAATACAGACTTCACTGCAAACCATTGTTGAGTC
Exon 21
pseudoexon
d
Splice donor
EcoRI
Intron 37
(916 bp)
NdeI
c.7120+1475A
pSPL3-USH2A
c.7120+1475A
(6843 bp)
AmpR
Splice acceptor
Splice donor
EcoRI
Intron 64
(845 bp)
NdeI
c.14134-3169A
pSPL3-USH2A
c.14134-3169A
(6772 bp)
AmpR
Splice acceptor
Splice donor
EcoRI
NdeI
Intron 21
(745 bp)
c.4628-26037A
pSPL3-USH2A
c.4628-26037A
(6738 bp)
BamHI
NdeI
AmpR
Splice acceptor
Restriction enzyme digestion assay
(EcoRI and NdeI)
(bp)
(kbp)
Long exp
Short exp
pSPL3 empty vector
c.7120+1475A (wt)
c.14134-3169A (wt)
c.4628-26037A (wt)

Figure S14. Analysis of *USH2A* intronic variants and their impact on splicing.

(a) *USH2A* c.7120+1475A>G, (b) c.14135-3169A>G, and (c) c.4628-26037A>G minigenes produced a pseudoexon containing a premature stop codon. Sanger sequencing confirmed the pseudoexon sequence. The splicing event leading to pseudoexon formation introduces a stop codon immediately after the normal exon sequence. (d) Schematic representation of USH2A minigene constructs containing intronic mutation sites. The constructs include intron 37 (c.7120+1475A), intron 64 (c.14135-3169A), and intron 21 (c.4628-26037A) cloned into the pSPL3 vector between the exon splice donor and splice acceptor sites. Restriction enzyme digestion using EcoRI and NdeI confirmed the successful insertion of *USH2A* intron sequences into the empty vector, as demonstrated by gel electrophoresis.

a

Genetic Testing Step	Cost ($)	TAT (Week)
Step1<br>(PCR-based screening kit & GJB2 sequencing)	100	1
Step 2-1<br>(TPS or WES)	500	4
Step 2-2<br>(MLPA or mtDNA sequencing)	300	2
Step 3<br>(WGS)	1000	4

b

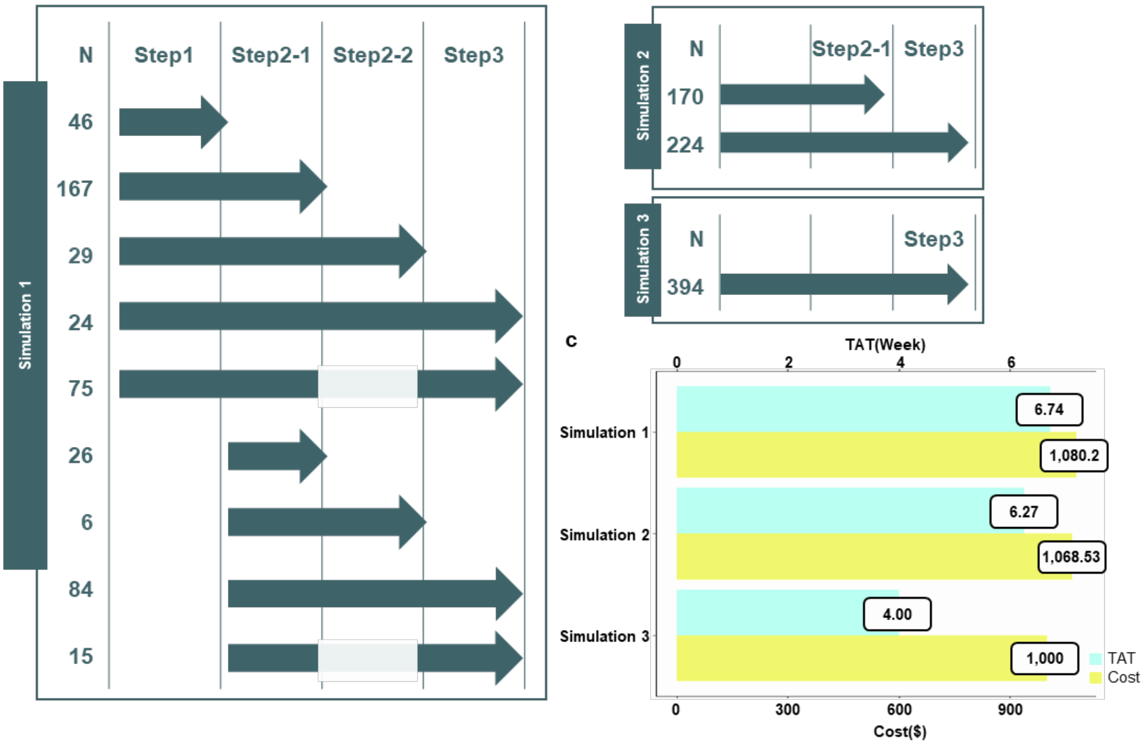

Figure S15. Cost and turnaround time (TAT) analysis across the three genetic diagnostic approaches.

(a) Summary of cost and TAT according to genetic test steps. (b) Details on flow according to three genetic diagnostic approaches. Simulation 1, stepwise diagnostic process used in our cohort. Simulation 2, direct WES followed by WGS without intermediate tests. Simulation 3, direct WGS without intermediate tests. (c) Comparison of cost and TAT by three genetic diagnostic approaches.

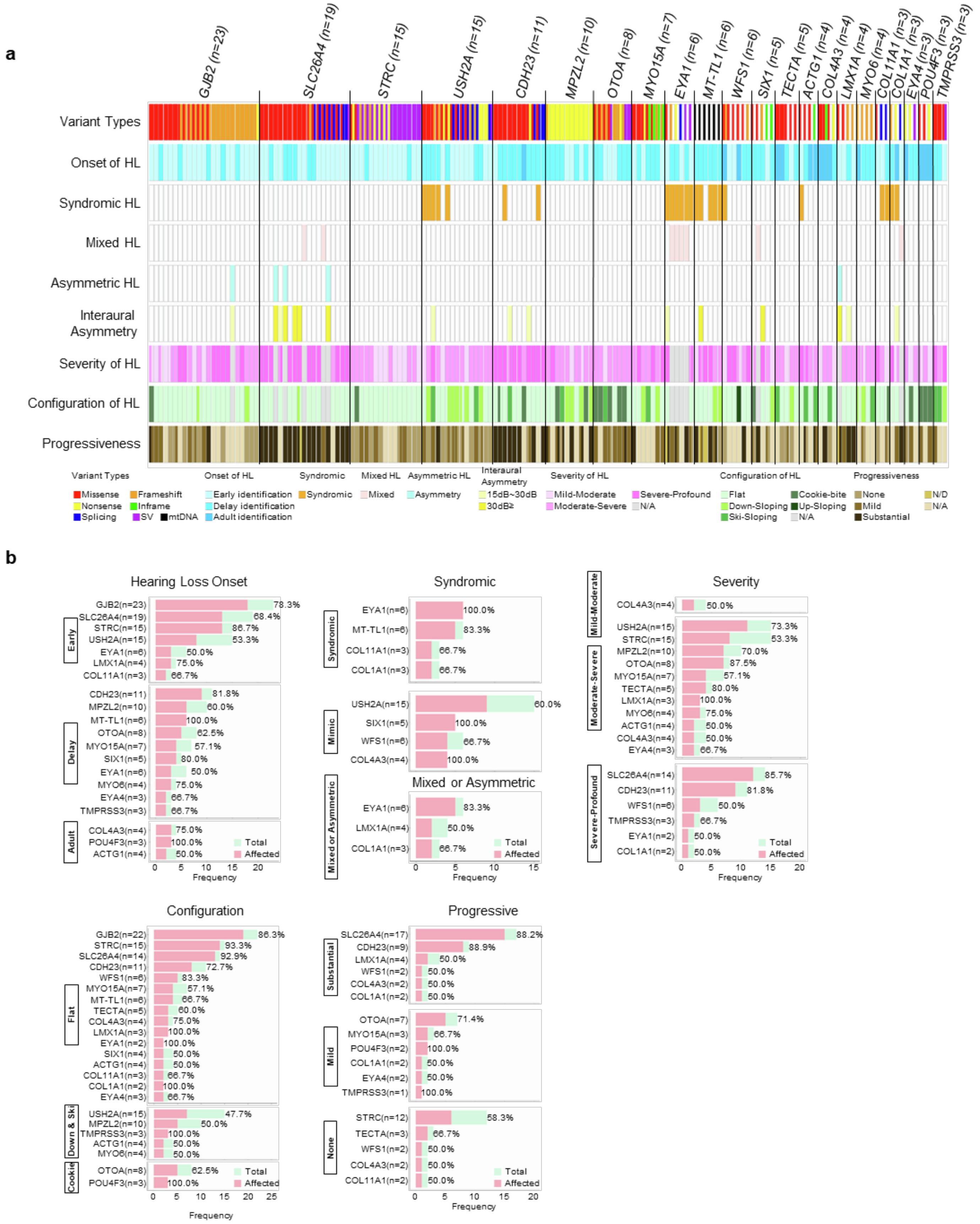

Figure S16. Heatmap and functional categorization of genetic variants associated with SNHL

(a) A heatmap illustrating the variant types and clinical phenotypes of each identified mutated gene in our SNHL cohort (i.e., Rare-grid plot). The “Rare-grid plot” includes 22 genes that were each detected in at least three unrelated families. Each column within this "Rare-grid plot" represents a proband with a genetic diagnosis. In the asymmetric hearing loss and interaural asymmetry columns, patients with a mixed hearing loss phenotype were excluded. In the severity and

configuration columns, both ears were separately depicted for patients without mixed hearing loss or asymmetric hearing loss phenotypes. In the progressiveness column, only patients meeting the criteria for hearing loss progression were included, categorized as substantial, mild, and none. (b) Gene signatures (i.e., over 50% of affected patients harboring variants in the same gene) according to hearing loss onset, syndromic features or nonsyndromic mimics, mixed or asymmetric hearing loss, hearing loss severity, hearing loss configuration, and progressiveness pattern. Green indicates the total count of genes corresponding to clinical phenotypes identified in our cohort study. Red indicates the subset of genes associated with specific clinical phenotypes. HL, hearing loss; SV, structure variant; mtDNA, mitochondrial DNA; N/A, not available; N/D, not determined.

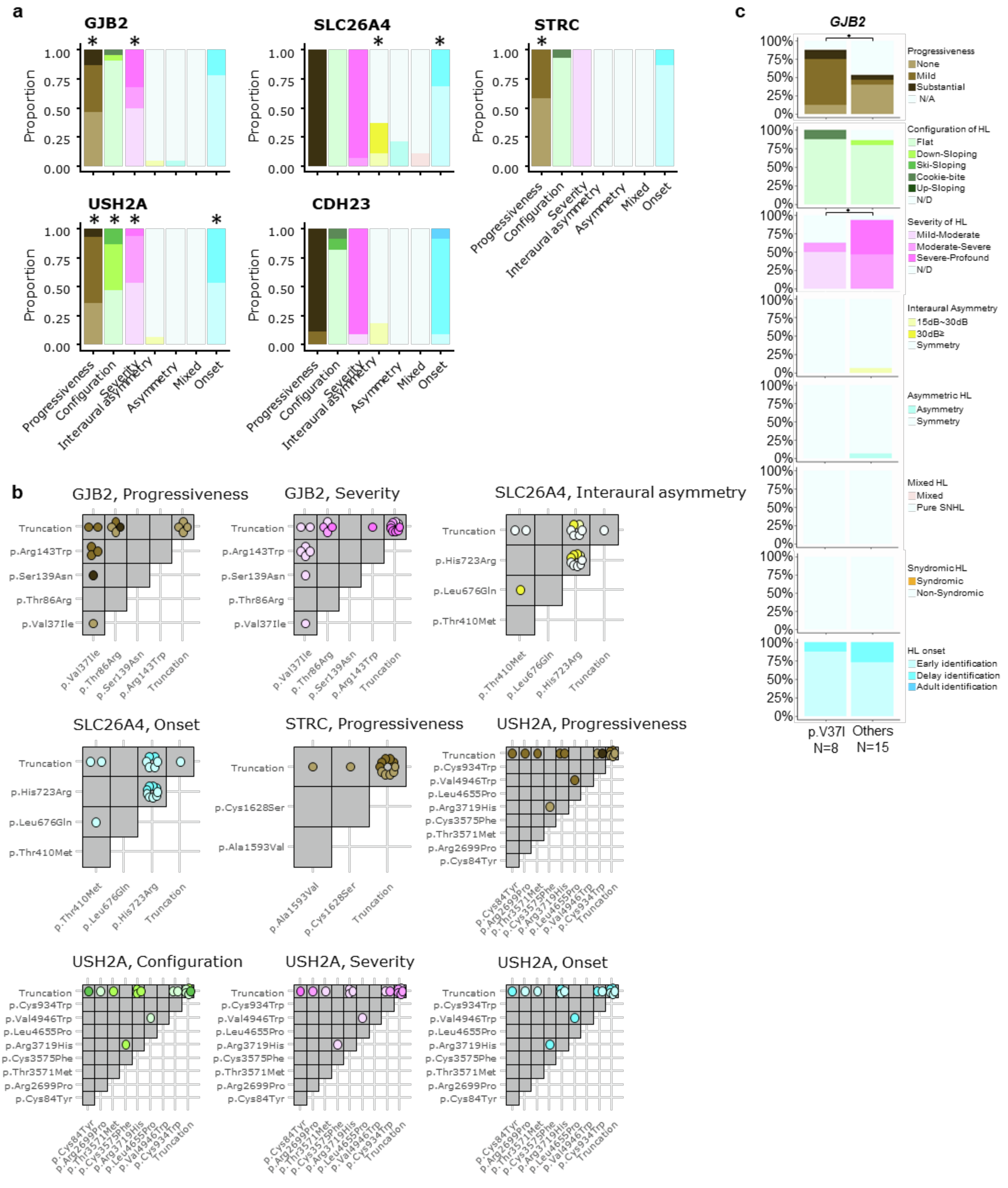

Figure S17. Phenotypic heterogeneity of recurrent pathogenic genes.

(a) Bar plots showing the composition of phenotypes in the top five recurrent genes within our cohort. Heterogeneous

gene-phenotype pairs, defined as 30% or more of a minor phenotype, are indicated with asterisks. *USH2A* displayed the highest heterogeneity in phenotypes among these five genes. (b) Grid plots illustrating the phenotype distributions for the combination of two variant types. The nine heterogeneous gene-phenotype pairs were selected from (a). The same combination of variants exhibited diverse phenotypes on many occasions. (c) Comparison of phenotypes by *GJB2* mutation allele (p.Val37Ile vs. other variants). Among probands with at least one p.Val37Ile allele, severity and progression of hearing loss were significantly lower and milder than in those without p.Val37Ile allele (P = 0.001 by Fisher's test).

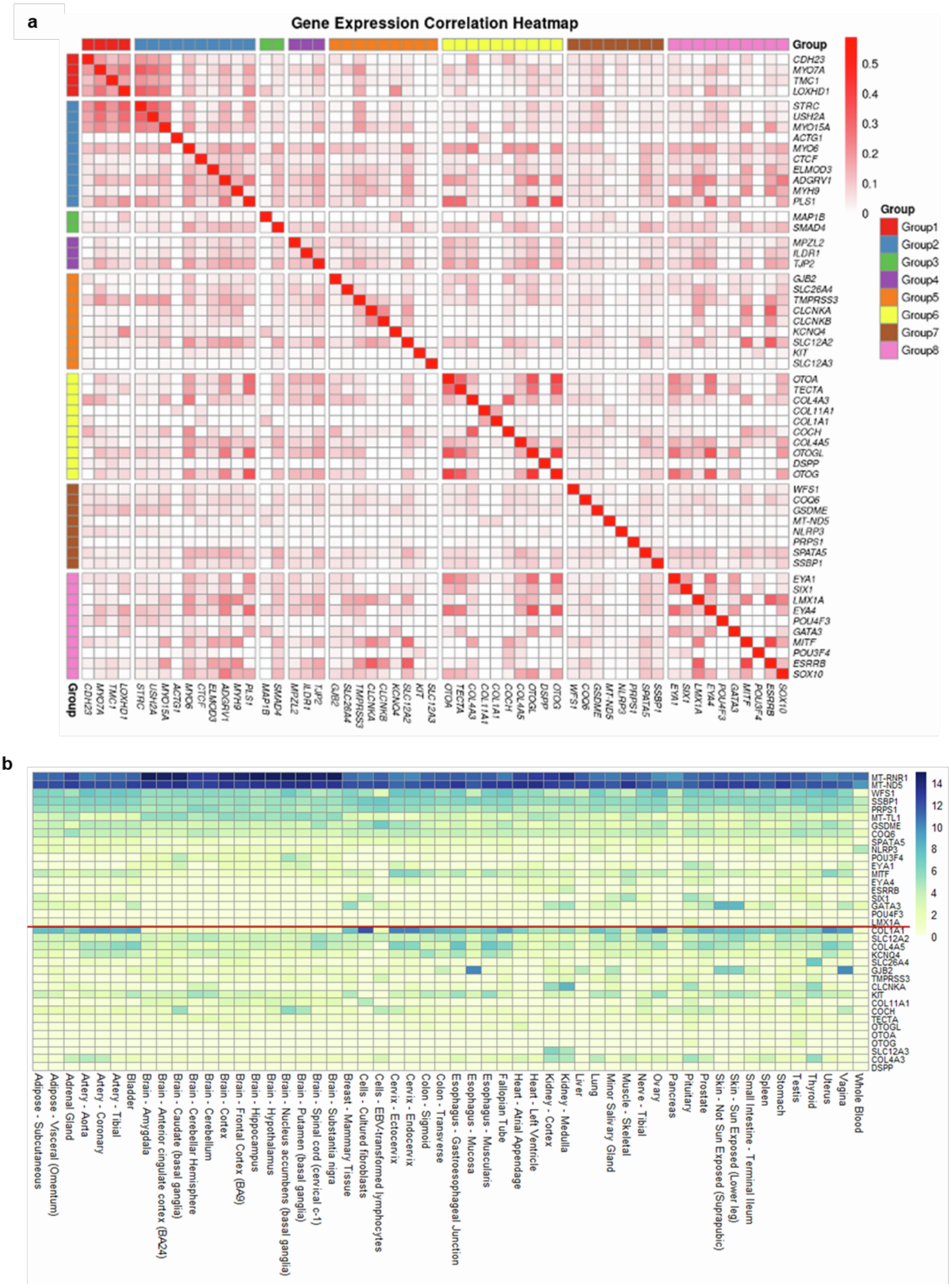

Figure S18. Gene expression profiles of SNHL genes across human organs or tissues

(a) The heatmap showing the correlation of gene expression patterns across different gene categories. (b) The heatmap visualizes the gene expression profiles of SNHL genes (Categories 5–8) across different human organs or tissues. Gene expression data were obtained from the GTEx portal (https://gtexportal.org/) and are represented on a log2TPM scale. Genes below the red line (Categories 5 and 6) likely have lower expression levels, while genes above the red line (Categories 7 and 8) tend to be more highly and broadly expressed in different organs or tissues.

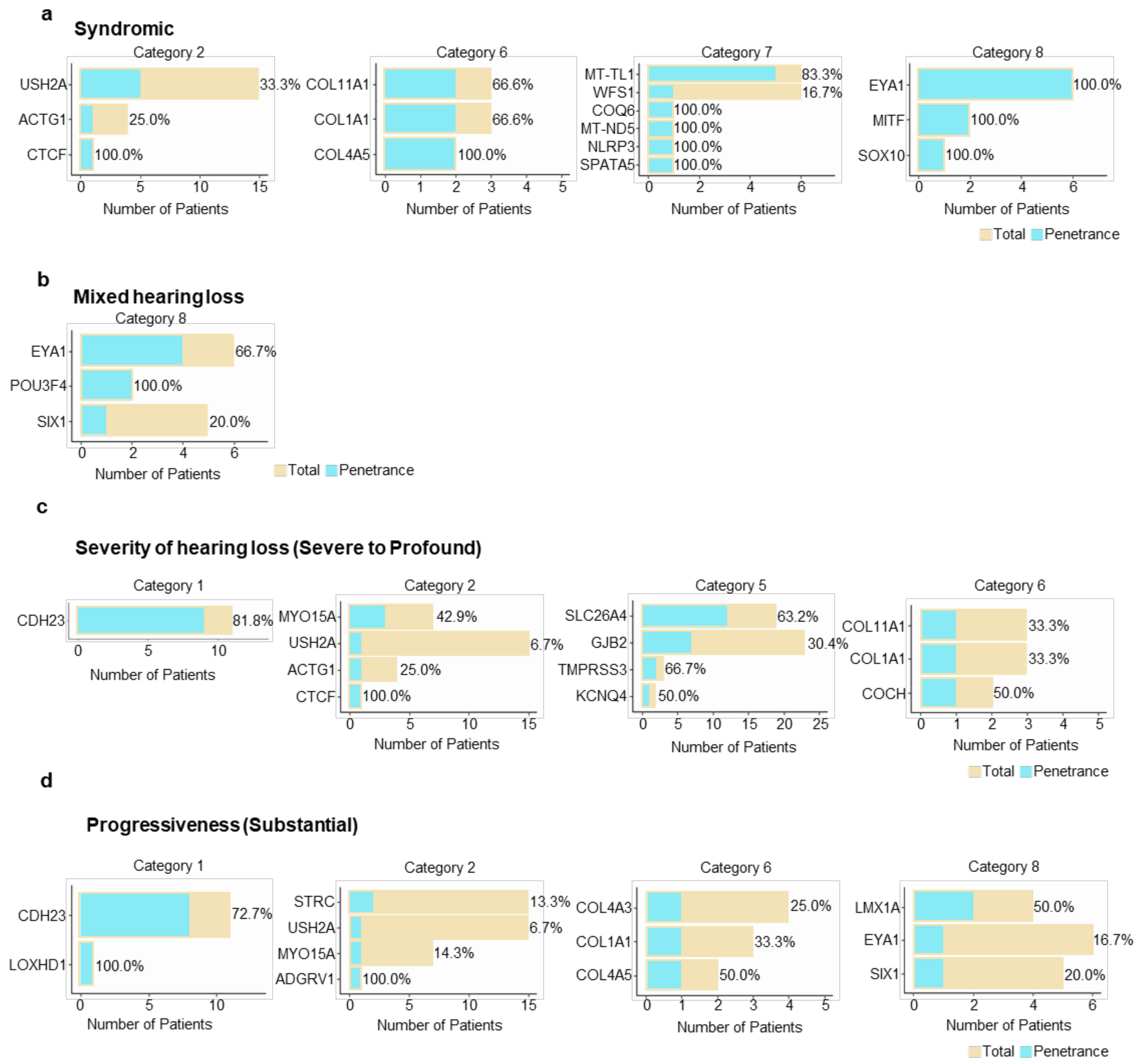

Figure S19. Penetrance of causative genes in each category with significant associations in Figure 5D.

(a) Significant associations between syndromic features and the functional categories, with detailed penetrance of each gene assigned to these categories. (b) Significant associations between mixed hearing loss and the functional categories, with detailed penetrance of each gene assigned to these categories. (c) Significant associations between severity of hearing loss (e.g., severe-to-profound) and the functional categories, with detailed penetrance of each gene assigned to these categories. (d) Significant associations between extent of progressive hearing loss and the functional categories, with detailed penetrance of each gene assigned to these categories. The x-axis represents the total number of probands (yellow bars) in each functional category, and the y-axis lists the causative genes in that category. Blue bars indicate the proportion of affected probands for each gene.

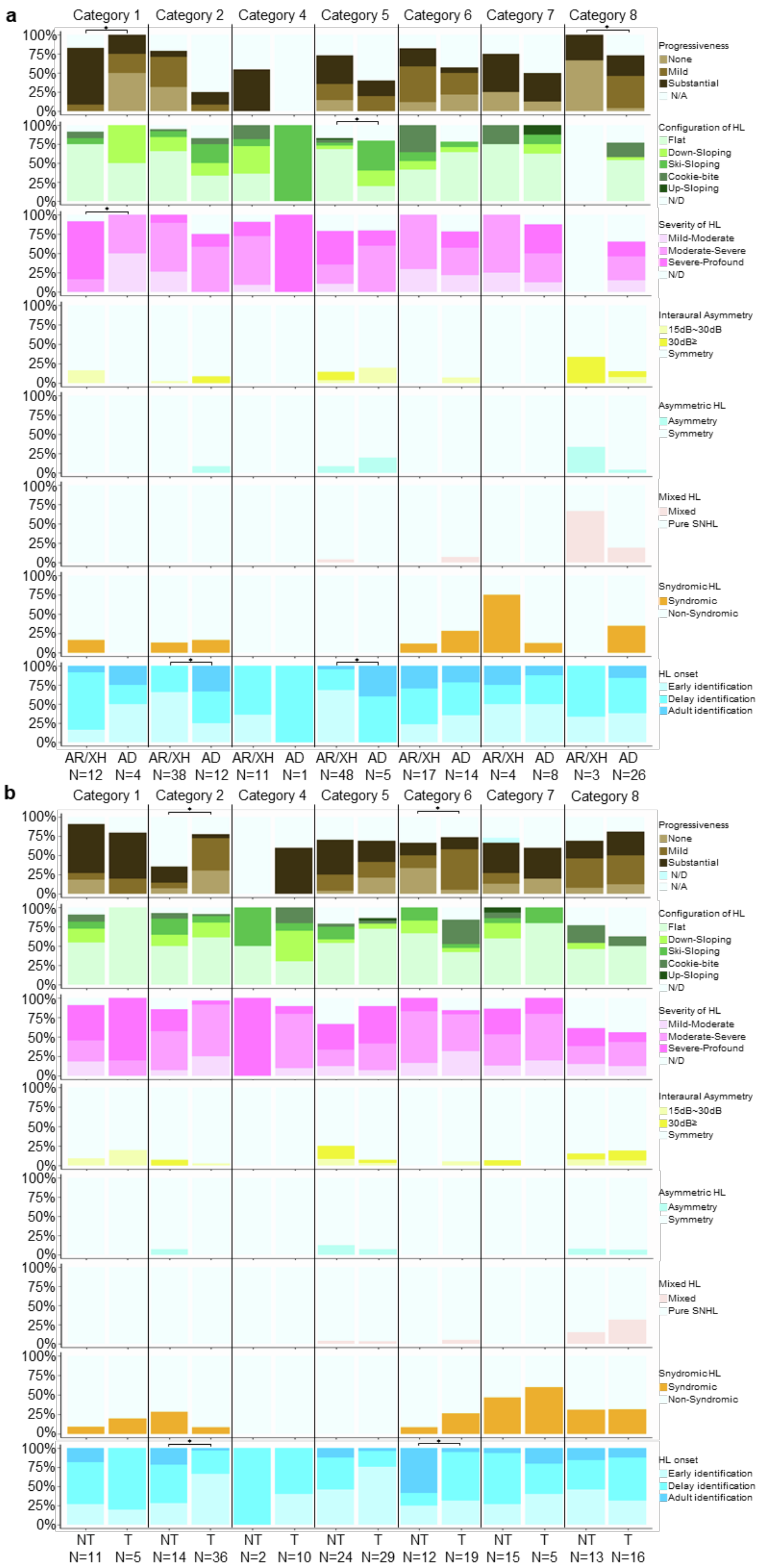
a
Category 1
Category 2
Category 4
Category 5
Category 6
Category 7
Category 8
100%
75%
50%
25%
0%
Progressiveness
None
Mild
Substantial
N/A
Configuration of HL
Flat
Down-Sloping
Ski-Sloping
Cookie-bite
Up-Sloping
N/D
Severity of HL
Mild-Moderate
Moderate-Severe
Severe-Profound
N/D
Interaural Asymmetry
15dB~30dB
30dB≥
Symmetry
Asymmetric HL
Asymmetry
Symmetry
Mixed HL
Mixed
Pure SNHL
Snydromic HL
Syndromic
Non-Syndromic
HL onset
Early identification
Delay identification
Adult identification
AR/XH N=12
AD N=4
AR/XH N=38
AD N=12
AR/XH N=11
AD N=1
AR/XH N=48
AD N=5
AR/XH N=17
AD N=14
AR/XH N=4
AD N=8
AR/XH N=3
AD N=26
b
Category 1
Category 2
Category 4
Category 5
Category 6
Category 7
Category 8
Progressiveness
None
Mild
Substantial
N/D
N/A
Configuration of HL
Flat
Down-Sloping
Ski-Sloping
Cookie-bite
Up-Sloping
N/D
Severity of HL
Mild-Moderate
Moderate-Severe
Severe-Profound
N/D
Interaural Asymmetry
15dB~30dB
30dB≥
Symmetry
Asymmetric HL
Asymmetry
Symmetry
Mixed HL
Mixed
Pure SNHL
Snydromic HL
Syndromic
Non-Syndromic
HL onset
Early identification
Delay identification
Adult identification
NT N=11
T N=5
NT N=14
T N=36
NT N=2
T N=10
NT N=24
T N=29
NT N=12
T N=19
NT N=15
T N=5
NT N=13
T N=16

Figure S20. Comparison of phenotypic presentations by inheritance patterns and mutation types within the same functional categories.

(a) Comparison of phenotypes by inheritance patterns (autosomal dominant vs. autosomal recessive or X-linked hemizygous). The x-axis shows the classification by inner-ear molecular functions, and the y-axis represents the proportion of clinical phenotypes (e.g., progressiveness, configuration, severity, asymmetry, type of hearing loss, syndromic features, and onset) in affected probands. Patients with mtDNA genes variants (*MT-TL1*, *MT-RNR1*, *MT-ND5*) in category 7 were excluded from the analysis. In Category 1, autosomal recessive or X-linked hemizygous cases showed greater severity (P = 0.005 by Fisher's test) and progressiveness (P = 0.018 by Fisher's test) than autosomal dominant cases. In Category 2, autosomal recessive or X-linked hemizygous cases had an earlier onset of hearing loss than autosomal dominant cases (P = 0.001 by Fisher's test). In Category 5, autosomal recessive or X-linked hemizygous cases had an earlier onset of hearing loss (P = 0.003 by Fisher's test) and displayed a significantly higher frequency of flat audiogram configurations instead of ski-slope patterns (P = 0.030 by Fisher's test), compared with autosomal dominant cases. In Category 8, autosomal dominant cases exhibited severe progressiveness (P = 0.02 by Fisher's test) than autosomal recessive or X-linked hemizygous cases. (b) Comparison of phenotypes by mutation type (truncated vs. non-truncated variants). In this analysis, variants such as frameshift, nonsense, canonical splicing-induced premature stop codon, and structural variations were classified as truncated variants. Conversely, missense variants and in-frame deletions/duplications were categorized as non-truncated variants. Hereafter, patients with at least one truncated allele will be referred to as the "truncated", while those with biallelic non-truncated alleles will be designated as the "non-truncated". In Category 2, non-truncated cases experienced a later onset of hearing loss (P = 0.02 by Fisher's test) but having substantial progressive hearing loss (P = 0.02 by Fisher's test), compared to truncated cases. In Category 6, truncated cases experienced an earlier onset of hearing loss (P = 0.002 by Fisher's test) and a tendency toward progressive nature (P = 0.008 by Fisher's test), compared with non-truncated cases.

Table S1. Demographics and clinical characteristics of SNHL cohort[a]

	N=394 families
**Sex (Proband)**	
Male	183(46.19)
**Age at Genetic Test, Median (range, year)**	13(0-76)
0~10	170(43.15)
11~20	61(15.48)
21~30	37(9.39)
31~40	30(7.61)
41~50	31(7.87)
51~60	38(9.64)
61~70	21(5.33)
71~76	6(1.52)
**Hearing Loss Onset**	
Early Identification (failed NHS)	148(37.56)
Delay Identification (passed NHS, pediatric onset)	163(41.37)
Adult Onset	83(21.06)
**Family History**	
Positive	87(22.08)
**Syndromic Features**	
Syndromic	53(13.45)
**Type of HL**	
Sensorineural HL	374(94.92)
Mixed HL	20(5.08)
**Asymmetry of HL[b]**	
Symmetric	338(90.37)
Asymmetric	36(9.63)
Interaural asymmetry(≥30dB)	40(10.70)
Interaural asymmetry(15-30dB)	26(6.60)
**Severity of HL[b] (Rt / Lt)**	
Mild-to-Moderate / Mild-to-Moderate	59(15.78)
Mild-to-Moderate / Moderate-to-Severe	13(3.48)
Mild-to-Moderate / Severe-to-Profound	6(1.60)
Moderate-to-Severe / Mild-to-Moderate	8(2.14)
Moderate-to-Severe / Moderate-to-Severe	147(39.30)
Moderate-to-Severe / Severe-to-Profound	19(5.08)
Severe-to-Profound / Mild-to-Moderate	2(0.53)
Severe-to-Profound / Moderate-to-Severe	15(4.01)
Severe-to-Profound / Severe-to-Profound	105(28.07)
**Configuration of HL[b] (Rt / Lt)**	
Flat / Flat	238(63.64)
Flat / Down-Sloping	8(2.14)
Flat / Cookie-Bite	3(0.80)
Flat / Up-Sloping	1(0.27)
Down-Sloping / Flat	5(1.34)
Down-Sloping / Down-Sloping	40(10.70)
Down-Sloping / Ski-Sloping	1(0.27)
Ski-Sloping / Flat	2(0.53)

Ski-Sloping / Down-Sloping	3(0.80)
Ski-Sloping / Ski-Sloping	36(9.63)
Cookie-Bite / Flat	2(0.53)
Cookie-Bite / Cookie-Bite	28(7.49)
Up-Sloping / Up-Sloping	7(1.87)
**Steps for Genetic Testing**	
Step1 (real-time PCR kit & GJB2 sequencing)	341(86.55)
Step2-1 (TPS/WES)	348(88.32)
TPS	99(25.13)
WES	249(63.20)
Step2-2 (MLPA & mtDNA panel sequencing)	65(16.50)
MLPA	54(13.71)
mtDNA	11(2.79)
Step3 (WGS)	120(30.46)
Singleton	63(15.99)
Duo	5(1.27)
≥Trio	52(13.20)

Abbreviations: SNHL, sensorineural hearing loss; HL, hearing loss; NHS, newborn hearing screening; Rt. Right; Lt, left; TPS, targeted-panel sequencing; WES, whole-exome sequencing; MLPA, Multiplex Ligation-dependent Probe Amplification; mtDNA, mitochondria DNA; WGS, whole-genome sequencing

[a]Data are presented as number (percentage) of patients in our study cohort.

[b]Patients with mixed hearing loss were excluded from the analysis.

Table S3. Clinical factors associated with genetic diagnosis of SNHL[a]

	Genetically Diagnosed (N=219, 55.6%)	Genetically Undiagnosed (N=175, 44.4%)	Crude OR (95%CI)	Adjusted OR[d] (95% CI)
Sex				
Male	103 (47.0)	80 (45.7)	1.05 (0.71-1.57)	
Hearing Loss Onset				
Early Identification (Failed NHS)	97 (44.3)	51 (29.1)	1.93 (1.27-2.95)*	1.32 (1.11-1.57)*
Delay Identification (passed NHS, pediatric onset)	96 (43.8)	67 (38.3)	1.26 (0.84-1.89)	
Adult Onset	26 (11.9)	57 (32.6)	0.28 (0.16-0.47)*	0.50 (0.35-0.68)*
Family History				
Positive	67 (30.6)	20 (11.4)	3.39(1.99-6.0)*	1.55 (1.31-1.82)*
Syndromic Features				
Positive	40 (18.3)	13 (7.4)	2.76 (1.46-5.56)*	1.44 (1.17-1.70)*
Type				
Mixed	13 (5.9)	7 (4.0)	1.50 (0.59-4.13)	
Asymmetry[b]	(n=206)	(n=168)		
Asymmetric	8 (3.9)	28 (16.7)	0.21 (0.08-0.45)*	0.38 (0.18-0.64)*
Interaural asymmetry (≥30dB)	10 (4.9)	30 (17.9)	0.24 (0.11-0.49)*	0.43 (0.23-0.68)*
Interaural asymmetry (15-30dB)	11 (5.3)	15 (8.9)	0.18 (0.22-0.17)	
Severity[c]	(n=183)	(n=127)		
Mild-Moderate	34 (18.58)	25 (19.7)	0.94 (0.53-1.68)	
Moderate-Severe	92 (50.27)	54 (42.5)	1.35 (0.86-2.14)	
Severe-Profound	57 (31.05)	48 (37.8)	0.75 (0.47-1.21)	
Configuration[c]	(n=190)	(n=133)		
Flat	125 (65.8)	88 (66.2)	0.98 (0.61-1.57)	
Down-Sloping	25 (13.2)	14 (10.5)	1.28 (0.64-2.64)	
Ski-Sloping	19 (10.0)	17 (12.8)	0.76 (0.38-1.54)	
Cookie-Bite	19 (10.0)	9 (6.8)	1.52 (0.68-3.66)	
Up-Sloping	2 (1.1)	5 (3.8)	0.29 (0.04-1.41)	

Abbreviations: SNHL, sensorineural hearing loss; OR, odds ratio; CI, confidence interval; NHS, newborn hearing screening.

[a]Data are presented as number (percentage) of patients in each group.

[b]Patients with mixed hearing loss were excluded from this analysis.

[c]Patients with discordant phenotypes of SNHL between the two ears, including severity and configuration, in conjunction with mixed hearing loss, were excluded from this analysis.

[d]Adjusted ORs (95% CIs) were calculated using multiple logistic regression, using the significant variables based on the Crude ORs.

*Statistically significant by Pearson's chi-squared test.

Table S4. Factors influencing the probability of genetic diagnosis by whole-genome sequencing[a]

	**Genetically Diagnosed (N=23,19.2%)**	**Genetically Undiagnosed (N=97,80.8%)**	**Crude OR (95%CI)**	**Adjusted OR[e] (95% CI)**
Sex				
Male	13 (56.5)	45 (46.4)	1.49 (0.59-3.85)	
Hearing Loss Onset				
Early Identification (Failed NHS)	12 (52.2)	26 (26.8)	2.94 (1.14-7.68)*	2.35(1.13-4.97)*
Late Identification (Passed NHS, Pediatric Onset)	8 (34.8)	40 (41.2)	0.77 (0.28-1.96)	
Adult Onset	3 (13.0)	31 (32.0)	0.33 (0.07-1.08)	
Family History				
Positive	7 (30.4)	13 (13.4)	2.81 (0.92-8.16)	
Syndromic Features				
Positive	8 (34.8)	13 (13.4)	3.41 (1.16-9.74)*	2.51 (1.15-5.04)*
WGS Type				
Singleton	6 (26.1)	57 (58.8)	0.25 (0.08-0.68)*	0.32 (0.12-0.71)*
Duo	0 (0.0)	5 (5.2)	-	
≥ Trio	17 (73.9)	35 (36.1)	4.88 (1.83-14.84)*	3.71 (1.67-9.68)*
Type				
Mixed	1 (4.3)	4 (4.1)	1.16 (0.04-8.89)	
Asymmetry[c]	(n=22)	(n=93)		
Asymmetric	1 (4.5)	12 (12.9)	0.36 (0.01-2.04)	
Interaural asymmetry (≥30dB)	0 (0.0)	13 (14.0)	-	
Interaural asymmetry (15-30dB)	3 (13.6)	10 (10.8)	1.35 (0.27-5.03)	
Severity[d]	(n=18)	(n=73)		
Mild-Moderate	0 (0.0)	17 (23.3)	-	
Moderate-Severe	12 (66.7)	35 (47.9)	2.13 (0.73-6.83)	
Severe-Profound	6 (33.3)	21 (28.8)	1.25 (0.38-3.72)	
Configuration[d]	(n=21)	(n=74)		
Flat	12 (57.1)	44 (59.5)	0.91 (0.34-2.51)	
Down-Sloping	4 (19.0)	7 (9.5)	2.26 (0.52-8.64)	
Ski-Sloping	2 (9.5)	15 (20.3)	0.44 (0.06-1.79)	
Cookie-Bite	1 (4.8)	6 (8.1)	0.63 (0.02-4.16)	
Up-Sloping	2 (9.5)	2 (2.7)	3.72 (0.37-37.70)	

Abbreviations: OR, odds ratio; CI, confidence interval; HL, hearing loss; NHS, newborn hearing screening; NSHL, non-syndromic hearing loss

[a]Data are presented as number (percentage) of patients in each group.

[b]Previously identified pathogenic genetic carrier during the genetic testing before WGS.

[c]Patients with mixed hearing loss were excluded from this analysis.

[d]Patients with discordant phenotypes of SNHL between the two ears, including severity and configuration, in conjunction with mixed hearing loss, were excluded from this analysis.

[e]Adjusted ORs (95% CIs) were calculated using multiple logistic regression, using the significant variables derived from the crude ORs.

*Statistically significant by Pearson’s chi-squared test.

Table S5. Detailed information of three outlier variants

Gene symbol	Variant information	dbSNP ID	Allele frequency			Disease entity listed in OMIM (ID)
			gnomAD (exome)	gnomAD (genome)	gnomAD (Grpmax)	
SLC12A3	NM_001126108.2:c.539C>A, p.Thr180Lys (chr16-56869762-C-A)	rs146158333	3.00E-04	2.00E-04	0.00841	Gitelman syndrome, AR (#263800)
ESRRB	NM_004452.4:c.1144C>T, p.Arg382Cys (chr14-76498300-C-T)	rs373131497	6.00E-04	3.00E-04	0.00705	Deafness, autosomal recessive 35, AR (#608565)
GJB2	NM_004004.6:c.109G>A, p.Val37Ile (chr13-20189473-C-T)	rs72474224	7.70E-03	4.00E-03	0.0415	Bart-Pumphrey syndrome, AD (#149200)<br>Deafness, autosomal dominant 3A, AD (#601544)<br>Deafness, autosomal recessive 1A, AR/DD (#220290)<br>Hystrix-like ichthyosis with deafness, AD (#602540)<br>Keratitis-ichthyosis-deafness syndrome, AD (#148210)<br>Keratoderma, palmoplantar, with deafness, AD (#148350)<br>Vohwinkel syndrome, AD (#124500)

Grpmax, group maximum; AR, autosomal recessive; AD, autosomal dominant; DD, digenic dominant

Table S7. Functional assays for variants uncertain significance and their pathogenicity reclassification

Family ID	Presenting clinical features	Gene symbol	Variant information	Functional assays	Molecular consequences	Reclassification (ACMG-AMP)
**Homozygous, hemizygous, or compound heterozygous sequence variants**						
SNUH485	SNHL (USH2 mimics)	*USH2A*	NM_206933.4:c.7120+1475A>G (p.?)	Minigene splicing assay	Aberrant splicing caused by pseudoexon inclusion	VUS → P (PVS1,PM2,PM3,PP1)
SNUH503	SNHL (USH2 mimics)	*USH2A*	NM_206933.4:c.14134-3169A>G (p.?)	Minigene splicing assay	Aberrant splicing caused by pseudoexon inclusion	VUS → P (PVS1,PM2,PM3,PP5)
SNUH513	SNHL (USH2 mimics)	*USH2A*	NM_206933.4:c.4628-26037A>G (p.?)	Minigene splicing assay	Aberrant splicing caused by pseudoexon inclusion	VUS → P (PVS1,PM2,PM3)
SNUH501	SNHL	*TMPRSS3*	NM_024022.3:c.743C>T (p.Thr248Met)	Molecular modeling	Loss of hydrogen bonds and reduced loop stability	VUS → LP (PS3,PM2,PM3,PP3)
				Yeast-based protease assay	Abolished proteolytic ctivity[1]	
SNUH635	SNHL	*ESRRB*	NM_004452.4:c.1144C>T (p.Arg382Cys)	Molecular modeling and dynamics	Disrupted intramolecular interactions and conformational changes leading to compromised loop stability	VUS → LP (PS3,PM3,PP3,PP5, BS1)
				RT-qPCR	Altered expression of downstream target genes	
				Luciferase reporter assay	Reduced transcriptional activity[2]	
**Structural variants**						
SNUH536	BOR/BO	*EYA1*	NC_000008.11:g.[71211857_71228236inv; 71211857_71215145del]	RT-qPCR	Decreased EYA1 mRNA expression and altered expression of downstream target genes	.
				Western blot	Decreased EYA1 protein expression	
				Luciferase reporter assay	Reduced transcriptional activity	
				CRISPR editing	Restored expression of mRNA and protein and improved transcriptional activity by paired gRNA with Cas9 nuclease or CRIPSR activator[3]	
SNUH481	SNHL	*CLCNKA*	NM_004070.4: c.1804_ [NC_000001.11:g.16046349]del	Digital droplet PCR	One copy loss of *CLCNKA*	.
SNUH799	NEDHSB	*SPATA5*	NM_145207.3:c.2227-3015_2354+1415del	Oxygen consumption rate	Reduced basal respiration, maximal respiration, and ATP production	.

Other variants						
SNUH529	Atypical BOR/BO	*SIX1*	NM_005982.4:c.501G>C (p.Gln167His)	3D modeling and structure analysis	Loss of hydrogen bonds between SIX1 and DNA phosphor backbone	VUS → LP (PS3,PM1,PM2,PP3)
				Western blot	Decreased SIX1 protein expression	
				Coimmunoprecipitation	Loss of EYA1-SIX1 interaction	
				DNA-binding assay	Decreased DNA binding affinity	
				Luciferase reporter assay	Reduced transcriptional activity[4]	
SNUH392	SNHL	*LMX1A*	NM_177398.4:c.719A>G (p.Gln240Arg)	Molecular modeling	Collapsed hydrogen bonding and steric hinderance between DNA double helix	VUS → LP (PS3,PM2,PP3,BP1)
				Luciferase reporter assay	Abolished transcriptional activity[5]	
SNUH421	SNHL	*LMX1A*	NM_177398.4:c.721G>A (p.Val241Met)	Molecular modeling	Reduced LMX1A-DNA interaction	VUS → LP (PS3,PM2,PM5,PP3)
				Luciferase reporter assay	Abolished transcriptional activity[5]	
SNUH497	SNHL	*KCNQ4*	NM_004700.4:c.1168C>T (p.Arg390Cys)	Immunocytochemistry	Stable plasma membrane trafficking	VUS → LP (PS3,PM1,PM2,PP3)
				Patch clamping	Impaired potassium ion conductance and channel activity	
SNUH386	SNHL	*TMC1*	NM_138691.3:c.1256T>C (p.Phe419Ser)	Molecular modeling	Altered hydrophobicity in the membrane and compromised protein-lipid interaction	VUS → LP (PS3,PM1,PM2,PP3)
				CHX chase assay	Rapid protein degradation[6]	
SNUH676	SNHL	*TMC1*	NM_138691.3:c.1444T>C (p.Trp482Arg)	Molecular modeling	Disrupts the cation-π interaction with phospholipids and destabilized protein-lipid interaction	VUS → LP (PS3,PM1,PM2)
				CHX chase assay	Rapid protein degradation[6]	
SNUH518	SNHL, Dentinogenesis	*DSPP*	NM_014208.3:c.51+5G>A (p.?)	Minigene splicing assay	Exon skipping-induced aberrant splicing	VUS → P (PVS1,PM2,PP1,PP3)
SNUH566	SNHL	*ELMOD3*	NM_001135022.2:c.640G>A (p.Gly214Ser)	3D modeling and structure analysis	Disrupted intramolecular interaction and structure instability	VUS → LP (PS3,PM1,PM2,PP3)

CHX chase assay	Rapid protein degradation
Immunocytochemistry	Failed to F-actin colocalization[7]

Abbreviations: SNHL, sensorineural hearing loss; BOR/BO, Branchio-oto-renal syndrome/branchio-otic syndrome; NEDHSB, Neurodevelopmental disorder with hearing loss, seizures, and brain abnormalities; RT-qPCR, real-time quantitative PCR; CHX, cycloheximide; VUS, variant of uncertain significance; LP, likely pathogenic.

1. Lee, S. J. et al. Structural analysis of pathogenic TMPRSS3 variants and their cochlear implantation outcomes of sensorineural hearing loss. Gene 865, 147335 (2023). https://doi.org:10.1016/j.gene.2023.147335
2. Choi, W. H. et al. Functional pathogenicity of ESRRB variant of uncertain significance contributes to hearing loss (DFNB35). Sci Rep 14, 21215 (2024). https://doi.org:10.1038/s41598-024-70795-8
3. Yi, H. et al. CRISPR-based editing strategies to rectify EYA1 complex genomic rearrangement linked to haploinsufficiency. Mol Ther Nucleic Acids 35, 102199 (2024). https://doi.org:10.1016/j.omtn.2024.102199
4. Lee, S. et al. Phenotypic and molecular basis of SIX1 variants linked to non-syndromic deafness and atypical branchio-otic syndrome in South Korea. Sci Rep 13, 11776 (2023). https://doi.org:10.1038/s41598-023-38909-w
5. Lee, S. Y. et al. Novel Molecular Genetic Etiology of Asymmetric Hearing Loss: Autosomal-Dominant LMX1A Variants. Ear Hear 43, 1698-1707 (2022). https://doi.org:10.1097/AUD.0000000000001237
6. Cho, S. H. et al. Novel autosomal dominant TMC1 variants linked to hearing loss: insight into protein-lipid interactions. BMC Med Genomics 16, 320 (2023). https://doi.org:10.1186/s12920-023-01766-7
7. Yun, Y. et al. Confirmatory insights into ELMOD3-associated autosomal dominant non-syndromic hearing loss. medRxiv 2025.02.11.25321773. https://doi.org/10.1101/2025.02.11.25321773

Table S9. Functional categories and the assigned gene list from the 63 identified SNHL-related genes

Gene symbol	Protein name	OMIM	Reference<br>(related to functional category)
**1. Auditory mechanoelectrical transduction machinery**			
CDH23	Cadherin related 23	605516	Petit et al. 2023 (PMID:37173518)<br>Delmaghani et al. 2020 (PMID:32708116)
MYO7A	Myosin VIIA	276903	Petit et al. 2023 (PMID:37173518)<br>Delmaghani et al. 2020 (PMID:32708116)
TMC1	Transmembrane channel like 1	606706	Petit et al. 2023 (PMID:37173518)<br>Delmaghani et al. 2020 (PMID:32708116)
LOXHD1	Lipoxygenase homology PLAT domains 1	613072	Delmaghani et al. 2020 (PMID:32708116)
**2. Actin cytoskeleton dynamics and stereocilia associated proteins**			
STRC	Stereocilin	606440	Delmaghani et al. 2020 (PMID:32708116)
USH2A	Usherin	608400	Zaw et al. 2022 (PMID:36041150)
MYO15A	Myosin XVA	602666	Petit et al. 2023 (PMID:37173518)<br>Delmaghani et al. 2020 (PMID:32708116)
ACTG1	Actin gamma 1	102560	Petit et al. 2023 (PMID:37173518)<br>Delmaghani et al. 2020 (PMID:32708116)
MYO6	Myosin VI	600970	Petit et al. 2023 (PMID:37173518)<br>Delmaghani et al. 2020 (PMID:32708116)
CTCF	CCCTC-binding factor	604167	Ma et al. 2018 (PMID:30107916)
ELMOD3	ELMO domain containing 3	615427	Petit et al. 2023 (PMID:37173518)<br>Delmaghani et al. 2020 (PMID:32708116)
ADGRV1	Adhesion G protein-coupled receptor V1	602851	McMillan et al. 2010 (PMID:21618827)
MYH9	Myosin heavy chain 9	160775	Petit et al. 2023 (PMID:37173518)<br>Delmaghani et al. 2020 (PMID:32708116)
PLS1	Plastin 1	602734	Petit et al. 2023 (PMID:37173518)<br>Delmaghani et al. 2020 (PMID:32708116)
**3. Synaptic transmission and spiral ganglion neurons deficiency**			
MAP1B	Microtubule-associated protein 1B	157129	Cui et al. 2020 (PMID:33268592)
SMAD4	SMAD family member 4	600993	Liu et al. 2016 (PMID:26491026)
**4. Hair cell adhesion and maintenance**			
MPZL2	Myelin protein zero like 2	604873	Delmaghani et al. 2020 (PMID:32708116)
ILDR1	Immunoglobulin like domain containing receptor 1	609739	Delmaghani et al. 2020 (PMID:32708116)
TJP2	Tight junction protein 2	607709	Delmaghani et al. 2020 (PMID:32708116)
**5. Cochlear ion homeostasis**			
GJB2	Gap junction protein beta 2	121011	Petit et al. 2023 (PMID:37173518)<br>Delmaghani et al. 2020 (PMID:32708116)
SLC26A4	Solute carrier family 26 member 4	605646	Petit et al. 2023 (PMID:37173518)<br>Delmaghani et al. 2020 (PMID:32708116)

TMPRSS3	Transmembrane serine protease 3	605511	Petit et al. 2023 (PMID:37173518)<br>Delmaghani et al. 2020 (PMID:32708116)
CLCNKA	Chloride channel kidney A	602023,<br>602024	Rickheit et al. 2008 (PMID:18833191)<br>Yun et al. 2023 (PMID:38069401)
KCNQ4	Potassium voltage-gated channel subfamily Q member 4	603537	Petit et al. 2023 (PMID:37173518)<br>Delmaghani et al. 2020 (PMID:32708116)
SLC12A2	Solute carrier family 12 member 2	600840	Petit et al. 2023 (PMID:37173518)<br>Delmaghani et al. 2020 (PMID:32708116)
KIT	KIT proto-oncogene, receptor tyrosine kinase	164920	Xu et al. 2020 (PMID:33042408)
SLC12A3	Solute carrier family 12 member 3	600968	Møller et al. 2015 (PMID:25486439)
**6. Transmembrane and extracellular matrix**			
OTOA	Otoancorin	607038	Delmaghani et al. 2020 (PMID:32708116)
TECTA	Tectorin alpha	602574	Delmaghani et al. 2020 (PMID:32708116)
COL4A3	Collagen type IV alpha-3	120070	Cosgrove et al. 1998 (PMID:9682811),<br>Dufek et al. 2020 (PMID:32234583)
COL11A1	Collagen type XI alpha-1	120280	Delmaghani et al. 2020 (PMID:32708116)
COL1A1	Collagen type I alpha-1	120150	Bonadio et al. 1990 (PMID:2402497)
COCH	Cochlin	603196	Delmaghani et al. 2020 (PMID:32708116)
COL4A5	Collagen type IV alpha-5	303630	Cosgrove et al. 1998 (PMID:9682811),<br>Dufek et al. 2020 (PMID:32234583)
OTOGL	Otogelin like	614925	Delmaghani et al. 2020 (PMID:32708116)
DSPP	Dentin sialophosphoprotein	125485	Xiao et al. 2001 (PMID:11175790)
OTOG	Otogelin	604487	Delmaghani et al. 2020 (PMID:32708116)
**7. Oxidative stress, autoinflammation, and mitochondria**			
MT-TL1	Mitochondrially encoded tRNA leucine 1	590050	Chung et al. 2022 (PMID:34836781)
WFS1	Wolframin ER transmembrane glycoprotein	606201	Delmaghani et al. 2020 (PMID:32708116)
COQ6	Coenzyme Q6, monooxygenas	614650	Heeringa et al. 2011 (PMID:21540551)
GSDME	Gasdermin E	608798	Petit et al. 2023 (PMID:37173518)<br>Delmaghani et al. 2020 (PMID:32708116)
MT-ND5	Mitochondrially encoded NADH dehydrogenase 5	516005	Chung et al. 2022 (PMID:34836781)
MT-RNR1	Mitochondrially encoded 12S RNA	561000	Petit et al. 2023 (PMID:37173518)<br>Delmaghani et al. 2020 (PMID:32708116)
NLRP3	NLR family pyrin domain containing 3	606416	Petit et al. 2023 (PMID:37173518)<br>Delmaghani et al. 2020 (PMID:32708116)
PRPS1	Phosphoribosyl pyrophosphate synthetase 1	311850	Delmaghani et al. 2020 (PMID:32708116)
SPATA5	Spermatogenesis associated protein 5	-	Tanaka et al. 2015 (PMID:26299366)
SSBP1	Single stranded DNA binding protein 1	600439	Cha et al. 2024 (PMID:39104869)
**8. Transcriptional regulation**			
EYA1	EYA transcriptional coactivator and phosphatase 1	601653	Niu et al. 2006 (PMID:16488112)
SIX1	SIX homeobox 1	601205	Petit et al. 2023 (PMID:37173518)<br>Delmaghani et al. 2020 (PMID:32708116)

LMX1A	LIM homeobox transcription factor 1 alpha	600298	Petit et al. 2023 (PMID:37173518)<br>Delmaghani et al. 2020 (PMID:32708116)
EYA4	EYA transcriptional coactivator and phosphatase 4	603550	Petit et al. 2023 (PMID:37173518)<br>Delmaghani et al. 2020 (PMID:32708116)
POU4F3	POU class 4 homeobox 3	602460	Petit et al. 2023 (PMID:37173518)<br>Delmaghani et al. 2020 (PMID:32708116)
GATA3	GATA binding protein 3	131320	Luo et al. 2013 (PMID:23666531)
MITF	Melanocyte inducing transcription factor	156845	Hai et al. 2017 (PMID:29094203)
POU3F4	POU class 3 homeobox 4	300039	Petit et al. 2023 (PMID:37173518)<br>Delmaghani et al. 2020 (PMID:32708116)
ESRRB	Estrogen related receptor beta	602167	Petit et al. 2023 (PMID:37173518)<br>Delmaghani et al. 2020 (PMID:32708116)
SOX10	SRY-box transcription factor 10	602229	Hai et al. 2017 (PMID:28639938),<br>Hao et al. 2018 (PMID:29922125)

Table S10. Distinct phenotypes according to inner ear functional categorizations

	Category 1 (N=16)	Category 2 (N=50)	Category 3 (N=2)	Category 4 (N=12)	Category 5 (N=53)	Category 6 (N=31)	Category 7 (N=20)	Category 8 (N=29)	*P*-value	Significant Pair (*P*-value)
Hearing loss onset										
Early Identification	4(25.0)	28(56.0)	1(50.0)	4(33.3)	33(62.3)	9(29.0)	6(30.0)	11(37.9)	0.01*	
Delay Identification	10(62.5)	18(36.0)	1(50.0)	8(66.7)	16(30.2)	14(45.2)	12(60.0)	14(48.3)	0.07	
Adult onset	2(12.5)	4(8.0)	0(0.0)	0(0.0)	4(7.6)	8(25.8)	2(10.0)	4(13.8)	0.26	
Syndromic HL	2(12.5)	7(14.0)	1(50.0)	0(0.0)	0(0.0)	6(19.4)	10(50.0)	9(31.0)	0.00*	2&5(0.02), 2&7(0.02), 4&7(0.02), 5&6(0.02), 5&7(0.00), 5&8(0.00)
Mixed HL	0(0.0)	0(0.0)	1(50.0)	0(0.0)	2(3.8)	1(3.2)	0(0.0)	7(24.1)	0.00	2&8(0.02)
Asymmetricity										
Asymmetric of HL	0(0.0)	1(2.0)	0(0.0)	0(0.0)	5(9.4)	0(0.0)	0(0.0)	2(6.9)	0.36	
Interaural Asymmetry 15~30dB	2(12.5)	1(2.0)	1(50.0)	0(0.0)	3(5.7)	1(3.2)	0(0.0)	2(6.9)	0.13	
Interaural Asymmetry 30dB ≥	0(0.0)	1(2.0)	0(0.0)	0(0.0)	5(9.4)	0(0.0)	1(5.0)	3(10.3)	0.32	
Severity of HL										
Mild-Moderate	2(12.5)	10(20.0)	0(0.0)	1(8.3)	5(9.4)	8(25.8)	3(15.0)	4(13.8)	0.63	
Moderate-Severe	4(25.0)	31(62.0)	1(50.0)	7(58.3)	15(28.3)	17(54.8)	9(45.0)	8(27.6)	0.00*	2&5(0.02)
Severe-Profound	9(56.2)	6(12.0)	0(0.0)	3(25.0)	22(41.5)	3(9.7)	6(30.0)	5(17.2)	0.00*	1&2(0.01), 1&6(0.01), 2&5(0.01), 5&6(0.02)
Configuration of HL										
Flat	11(68.8)	29(58.0)	0(0.0)	4(33.3)	34(64.2)	16(51.6)	13(65.0)	14(48.3)	0.24	
Down-Sloping	2(12.5)	9(18.0)	0(0.0)	4(33.3)	3(5.7)	3(9.7)	3(15.0)	1(3.5)	0.11	
Ski-Sloping	1(6.3)	6(12.0)	1(50.0)	2(16.7)	4(7.6)	3(9.7)	2(10.0)	0(0.0)	0.20	
Cookie-Bite	1(6.3)	2(4.0)	0(0.0)	2(16.7)	2(3.8)	6(19.4)	1(5.0)	5(17.2)	0.09	
Up-Sloping	0(0.0)	0(0.0)	0(0.0)	0(0.0)	1(1.9)	0(0.0)	1(5.0)	0(0.0)	0.50	
Progressiveness										
None	2(12.5)	12(24.0)	0(0.0)	0(0.0)	7(13.2)	5(16.1)	3(15.0)	3(10.3)	0.58	
Mild	2(12.5)	16(32.0)	1(50.0)	0(0.0)	11(20.8)	12(38.7)	2(10.0)	11(37.9)	0.02*	
Substantial	10(62.5)	5(10.0)	1(50.0)	6(50.0)	19(35.8)	5(16.1)	8(40.0)	8(27.6)	0.00*	1&2(0.02), 1&6(0.02), 2&4(0.03), 2&5(0.02), 2&7(0.04)

Abbreviations: HL, hearing loss; *, statistical significance ($P < 0.05$).
