# Supplementary material for: Comprehensive genetic profiling of sensorineural hearing loss using an integrative diagnostic approach
Source: Cell Rep Med. 2025 Jun 30;6(7):102206. doi: 10.1016/j.xcrm.2025.102206 (PMC12281402; doi:10.1016/j.xcrm.2025.102206)
Supplement: Data S1. Informed consent form for patient with SNHL [file mmc5.zip › Hereditary_Hearing_Loss_Consent_EN.docx]

# Research Participant (Adult) Information and Consent Form

Participant Information Sheet

## 1. Title of Clinical Study

Mechanistic Study for Diagnosis and Therapeutic Development of Hereditary Hearing Loss

## 2. Principal Investigator

Prof. Sang‑Yeon Lee, Department of Otorhinolaryngology, Seoul National University Hospital

## 3. Overview

This study examines the causative genes underlying hereditary hearing loss disorders. You have been invited to take part because you or your child is affected by hereditary hearing loss, or because you have been asked to confirm whether you have a hereditary predisposition. The investigator, Prof. Sang‑Yeon Lee (Department of Otorhinolaryngology, Seoul National University Hospital), will outline the study procedures to you. Participation is entirely voluntary. Before you decide, it is important that you understand why the study is being conducted, how your information will be used, what the study involves, and the possible benefits, risks, and inconveniences. Please read the following explanation carefully and take as much time as you need. Feel free to discuss it with your doctor, family, or friends. If you have questions, the study staff will answer them in detail.

## 4. Purpose of the Study

The purpose of this study is to determine whether your (or your child’s) hearing loss is caused by mutations in specific genes associated with hereditary hearing loss. If such mutations are identified, they may help guide treatment decisions. Whole‑genome sequencing (WGS) or whole‑exome sequencing (WES) may be used for this research.

## 5. Anticipated Side Effects, Risks, and Inconveniences

### a. Blood sampling

— Five millilitres of blood will be drawn to extract and analyse your DNA. Whenever possible, this will be collected at the same time as clinically required blood tests.

— If no pathogenic variants are detected in the blood sample, or if further study of genes highly expressed in dermal fibroblasts is required, a small skin biopsy may be obtained with your consent. This procedure will be performed under local anaesthesia in the outpatient procedure room using a sterile dermal punch (SPICA biopsy punch) by Prof. Sang‑Yeon Lee. The sample will be taken from the left forearm or behind the ear, measuring approximately 0.3 cm in diameter and 0.2 cm in depth. Because only a tiny amount of tissue is collected, visible scarring is expected to be minimal.

— After sampling, the wound will be sutured. Sutures are usually removed after one week. Although serious complications are unlikely, additional suturing may be required if wound closure is inadequate. Outpatient follow‑up and wound care will be provided. Should a scar develop, topical scar treatment will be offered free of charge upon request.

### b. Issues related to WGS/WES

— The genetic data obtained will primarily be used to identify pathogenic variants and may also be used to develop predictive software.

— Because genetic information can pose privacy and psychosocial risks (e.g., stigma or discrimination), all data will be anonymised and strictly protected from unauthorised disclosure. Research data will be destroyed after a required retention period.

— You may request disclosure of any personal information obtained during the study.

## 6. Expected Benefits

Participation does not guarantee direct medical benefit. If a pathogenic variant is identified, the information may assist in determining treatment. It is also possible that no variant will be found. The results will be explained during your outpatient visit.

## 7. Ongoing Provision of New Information

If information arises during the study that may influence your decision to continue participation, you or your representative will be informed immediately.

## 8. Confidentiality

Identifiable records will be kept confidential. If results are published, your identity will not be revealed. Data will be stored securely under the responsibility of Prof. Seung‑Ha Oh, Department of Otorhinolaryngology, Seoul National University Hospital, and protected by law as part of the medical record.

## 9. Voluntary Participation

Your participation is voluntary. You may refuse or withdraw at any time without penalty or loss of benefits to which you are otherwise entitled, and without affecting your future medical care.

## 10. Human Biospecimens

Biospecimens collected will be stored, provided, or discarded according to the storage period and secondary‑use preferences you indicate in the Biospecimen Research Consent Form. You may withdraw your consent at any time. Once the storage period you select has elapsed, specimens will be destroyed in accordance with the Waste Management Act. If the study terminates abnormally, specimens will be transferred following legal procedures. You will not have proprietary rights to any products or patents that may result. Research findings may be presented in conferences or journals under the investigators’ names without revealing your personal information.

## 11. Study Contacts

For further information, you may contact your treating physician, Prof. Sang‑Yeon Lee (telephone 02‑2072‑1478), at any time during the study. Questions about your rights as a participant may be directed to the Institutional Review Board of Seoul National University Hospital (telephone 02‑2072‑0694).

## Participant Consent

I have received an oral explanation and have read the above information sheet. I have discussed this study with the study staff and have had all of my questions answered. I voluntarily agree to participate. I understand that I may refuse or withdraw at any time without affecting my future care. By signing this form, I agree that my personal data may be collected and processed within the limits of applicable laws and regulations for the purposes of this medical research. I will receive a copy of this consent form.

Participant Name ______________________ Signature __________ Date (YYYY/MM/DD)

Person Obtaining Consent ______________ Signature __________ Date (YYYY/MM/DD)

Principal Investigator _________________ Signature __________ Date (YYYY/MM/DD)

— If applicable —

Legal Representative __________________ Signature __________ Date (YYYY/MM/DD)

Witness ______________________________ Signature __________ Date (YYYY/MM/DD)

# B. Participant (Minor) Information and Assent Form

Study Title: Mechanistic Study for Diagnosis and Therapeutic Development of Hereditary Hearing Loss

Principal Investigator: Prof. Seung‑Ha Oh, Department of Otorhinolaryngology, Seoul National University Hospital

Some words in this document may be hard to understand. If anything is unclear, please ask the study doctor or staff (Prof. Sang‑Yeon Lee, 2072‑1478).

## 1. Why is this study being done?

The investigator wants to learn more about children who have hereditary hearing loss. We will explain the study to you and ask whether you want to join.

## 2. Why am I being asked?

Children across the country who have hereditary hearing loss, or who want to know whether they have it, may join. The study team thinks you may be one of those children and is asking if you wish to participate.

## 3. Do I have to take part?

No. You can say no, and nothing bad will happen.

## 4. What will happen in the study?

We will take a small amount of your blood. We may also ask your parent or guardian some questions. If you and your parent agree, information from this study may be shared with other researchers, but your name will not be given.

If no results are found in your blood, or more tests are needed, we may take a very small piece of skin from your arm or behind your ear. The area will be numbed first, so it will only hurt at the moment of anaesthesia. The skin will be stitched, and the stitches will be removed after one week.

## 5. Will this study help me?

This study may not help you personally, but it may help children like you in the future.

## 6. What if I have questions?

Ask the study doctor, study staff, your parents, or guardian about anything you don’t understand. You can also read the ‘Parent Information Sheet’.

## 7. What happens to the blood and skin samples?

Your samples will be stored at Seoul National University Hospital for the period written in the Biospecimen Research Consent Form. They may be used for other research if you allow it, and will be destroyed after the period ends. Research results may be published, but your personal information will not be revealed.

Please keep a copy of this sheet. Your parents or legal guardian must also sign a separate consent form for you to participate.

If you want to join, please sign below.

1. I have read this sheet.

2. All of my questions have been answered.

3. I agree to join this study.

Child Participant Name ____________ Signature _________ Date (YYYY/MM/DD)

Person Obtaining Assent __________ Signature _________ Date (YYYY/MM/DD)

Principal Investigator ____________ Signature _________ Date (YYYY/MM/DD)

# Legal Representative Consent Form

1. I have received an oral explanation and have read the Information Sheet with the study staff.

2. I have been told about the risks and benefits and had my questions answered.

3. On behalf of the participant (my child), I consent to participate in this study.

4. I know that the participant may refuse or withdraw at any time without affecting future treatment.

5. By signing, I agree that the participant’s personal data may be collected and processed within the limits of the law for research purposes.

6. I will receive a copy of this consent form.

Participant Name ______________________ Signature __________ Date (YYYY/MM/DD)

Person Obtaining Consent ______________ Signature __________ Date (YYYY/MM/DD)

Principal Investigator _________________ Signature __________ Date (YYYY/MM/DD)

(if applicable)

Legal Representative __________________ Signature __________ Date (YYYY/MM/DD)

Witness ______________________________ Signature __________ Date (YYYY/MM/DD)
