# Supplementary material for: Comprehensive genetic profiling of sensorineural hearing loss using an integrative diagnostic approach
Source: Cell Rep Med. 2025 Jun 30;6(7):102206. doi: 10.1016/j.xcrm.2025.102206 (PMC12281402; doi:10.1016/j.xcrm.2025.102206)
Supplement: Data S1. Informed consent form for patient with SNHL [file mmc5.zip › Hereditary_Hearing_Loss_Consent_KOR.docx]

**연구대상자 (성인) 설명서 및 동의서**

**가. 연구대상자 (성인) 설명서 및 동의서**

**연구대상자 설명문**

**1. 임상 연구 제목**

유전성 난청의 진단, 치료법 개발을 위한 기전의 연구

**2. 시험 책임자**

서울대학교병원 이비인후과 이상연 교수

**3. 개요**

이 연구는 유전성 난청 질환의 원인 유전자에 대한 연구입니다. 귀하는 유전성 난청 질환 환자 (또는 환자의 부모)이거나 유전성 난청 소인 여부를 확인하기 위해 이 연구에 참여하도록 권유 받았습니다. 이 연구를 수행하는 서울대학교 이비인후과 소속 이상연 교수(연구책임자)가 귀하에게 이 연구 참여 과정에 대하여 설명해 줄 것입니다. 이 연구는 자발적으로 참여 의사를 밝히신 분에 한하여 수행될 것이며, 귀하께서는 본 임상 연구에 참여 의사를 결정하기에 앞서, 본 임상연구가 왜 수행되고, 귀하의 정보가 어떻게 사용될지, 본 임상연구가 어떤 것을 포함하고 있는 지와 가능한 이점, 위험, 불편함은 무엇인지에 대하여 이해하는 것이 중요합니다. 다음의 설명을 신중하게 시간을 가지고 주의 깊게 읽으시기 바라며, 필요하시면 귀하의 주치의 또는 가족이나 친구들과 상의하시기 바랍니다. 만일 어떠한 질문 사항이 있으시면 담당 연구원이 자세하게 설명해 줄 것입니다.

**4. 임상연구의 목적**

이 연구의 목적은 귀하 (또는 귀하의 자녀)가 난청이 있을 경우 이 원인이 유전성 난청 질환이 특정 유전자의 이상에 의한 것인지를 확인하는 것입니다. 유전성 난청 질환이 특정 유전자의 이상에 의한 것으로 밝혀질 경우, 이 질환에 대한 치료법을 결정하는 것에 도움을 받을 수 있습니다. 본 연구는 유전성 난청 질환의 연구를 위하여 전장 유전체 시퀀싱/전장 엑솜 시퀀싱(Whole Genome Sequencing/Whole Exome Sequencing) 기법을 연구에 활용할 수 있습니다.

**5. 연구대상자에게 예견되는 부작용, 위험과 불편함**

a. 부작용과 불편함

- 이 연구는 귀하의 혈액 (5mL)을 채취하여 해당 세포로부터 귀하의 유전자를 추출하여 검사하는 것입니다. 따라서 귀하로부터 5mL의 혈액을 채취하여야 하며 이는 다른 혈액 검사와 마찬가지 방법이며, 가능한 경우 귀하의 진료에 필요한 혈액검사를 시행할 당시 함께 채취할 것입니다.

- 만약 위의 혈액 샘플에서 유전자 돌연변이가 검출되지 않거나, 피부 내 섬유아모세포에 발현이 많이 되는 난청 유전자의 기전 및 치료법 연구가 필요한 경우에는 연구대상자의 피부 조직 일부를 추가적으로 채취하여 유전자 검사를 진행할 예정입니다. 피부 채취는 환자 혹은 환자 보호자의 동의를 얻은 후 진행할 예정이며, 모든 환자에서 진행하는 과정은 아닙니다. 시술은 대한외래 이비인후과 외래 처치실에서 국소 마취 하 무균 상태에서 dermal punch (SPICA biopsy punch) 제품을 이용해 시행할 예정입니다. 채취는 좌측 전완(forearm) 혹은 귀 뒤(retroauricular area) 부위에서 이상연 교수가 직접 진행합니다. 조직 샘플은 직경 0.3cm, 두께 0.2cm의 원형 모양으로 채취할 예정으로, 진단을 위해서만 극소량의 조직을 채취하기에 육안적으로 피부 조직을 채취한 부위의 흉터는 거의 보이지 않을 것입니다.

- 채취가 완료되면 피부 봉합을 시행합니다. 통상적으로 봉합 실은 1주일 뒤에 제거하도록 하며, 큰 합병증을 일으킬 가능성은 높지 않으나 봉합이 적절하게 완료되지 않아 피부 결손이 발생한다면 추가적인 피부 봉합술이 필요할 수 있습니다. 봉합 부위에 대한 주기적인 관리, 소독 및 외래 통원 치료를 지속할 수 있도록 외래 진료를 제공할 예정입니다. 조직 검사 이후 피부 반흔(흉터)이 발생할 가능성이 있으나 표피에 국한된 상처이므로 반흔의 크기는 굉장히 작을 것으로 예상합니다. 반흔의 발생에 대해서 환자 및 보호자가 원할 경우 본과 내에서 흉터 연고 등 적절한 치료를 무상으로 제공할 예정입니다.

b. 전장 유전체 시퀀싱/전장 엑솜 시퀀싱(Whole Genome Sequencing/Whole Exome Sequencing) 기법 적용에 따른 문제

- 얻어진 유전자 정보는 일차적으로 특정 유전자의 이상 여부를 확인하는데 쓰일 것이며 예후 예측을 위한 지능형 소프트웨어 개발을 위한 연구에 활용될 수 있습니다.

- 유전자 정보로 사생활 침해의 가능성 및 심리사회적 위험(사회적 오명과 차별 등)의 발생 가능성이 문제가 되고 있으므로, 환자의 유전자 정보는 외부유출이 엄격히 금지되며 연구과정에서도 익명화되어 처리됩니다. 연구가 종료되면 일정기간이 지난 후 연구데이터는 파기될 것입니다.

- 연구대상자는 연구과정에서 획득된 자기정보에 대해 공개 청구를 할 수 있습니다.

**6. 연구대상자에게 예견되는 이득**

귀하가 본 임상 연구에 참여함으로서 귀하에게 의학적 혜택이 보장되는 것은 아닙니다. 연구로 유전자 이상이 발견되는 경우에는 치료방침의 결정에 도움이 될 수 있습니다. 그러나 유전자 이상이 발견되지 않을 수도 있습니다.

연구로 얻은 유전정보에 관하여 해당 사실을 명시하고, 해당 유전정보를 대상자에게 제공할 예정입니다. 외래 진료시 유전분석 결과에 대한 설명을 드릴 예정입니다.

**7. 연구 관련 새로운 정보의 지속적 제공**

본 임상 연구 기간 중 귀하의 시험 참여 여부를 결정하는데 영향을 줄 수 있는 새로운 유의한 정보가 얻게 되는 즉시 귀하 또는 귀하의 대리인에게 알려 드릴 것입니다.

**8. 비밀 보장**

귀하의 신원을 파악할 수 있는 기록은 비밀로 보장될 것이며, 임상 연구의 결과가 출판될 경우 귀하의 신원은 비밀상태로 유지될 것입니다. 귀하의 자료는 연구책임자인 서울대학교병원 이비인후과 오승하 교수의 책임 하에 안전하게 보관될 것이며 귀하 (또는 귀하의 자녀)의 진료를 담당하는 의사에게 통보된 연구의 결과는 의무기록으로써 법률에 따라 그 비밀이 보장될 것입니다.

**9. 자발적 참여**

본 임상 연구에 참여하시는 것은 귀하에게 달려 있습니다. 귀하는 언제든지 시험에 참여하지 않기로 결정할 수 있고 또한 시험을 그만 둘 수 있습니다. 귀하가 본 연구에 참여하지 않아도 아무런 불이익을 받지 않으며 귀하의 결정은 향 후 귀하가 진료를 받는 것에 영향을 미치지 않습니다.

**10. 인체유래물 관련 사항**

연구대상자로부터 수집한 인체유래물은 연구대상자가 인체유래물연구동의서에 선택한 보존기간, 다른 연구 목적에 대한 제공 여부, 제공 시 개인정보 처리에 관한 사항에 따라 보관 및 폐기가 결정될 것입니다. 보존기간 내 2차적 사용에 동의하지 않은 연구대상자의 인체유래물은 보존 기간 이후 폐기한다. 보존기간 내 2차적 사용에 동의한 연구대상자의 인체유래물은 보존기간 내에 다른 연구 목적으로 제공이 가능하며 보존기간 이후의 인체유래물은 폐기한다.

귀하가 귀하의 인체유래물등을 아래의 연구 목적에 이용하도록 동의하는 경우, 귀하의 인체유래물등의 보존기간, 다른 사람 또는 다른 연구 목적에 대한 제공 여부, 제공 시 개인정보 처리에 관한 사항 및 폐기 등을 결정할 수 있습니다. 또한 동의한 사항에 대해 언제든지 동의를 철회할 수 있습니다.

귀하는 이 연구 참여와 관련하여 귀하의 동의서 및 귀하의 인체유래물등의 제공 및 폐기 등에 관한 기록을 본인 또는 법정대리인을 통하여 언제든지 열람할 수 있습니다.

귀하가 결정한 보존기간이 지난 인체유래물은 「폐기물관리법」 제13조에 따른 기준 및 방법에 따라 폐기되며, 해당 기관의 휴업ㆍ폐업 등 해당 연구가 비정상적으로 종료될 때에는 법에서 정한 절차에 따라 인체유래물등을 이관할 것입니다.

귀하의 인체유래물등을 이용한 연구결과에 따른 새로운 약품이나 진단도구 등 상품개발 및 특허출원 등에 대해서는 귀하의 권리를 주장할 수 없으며, 귀하가 제공한 인체유래물등을 이용한 연구는 학회와 학술지에 연구자의 이름으로 발표되고 귀하의 개인정보는 드러나지 않을 것입니다.

**11. 임상연구 관련 책임자 및 연락처**

귀하는 귀하 (또는 귀하의 자녀)의 진료를 담당하는 의사 (이상연 02-2072-1478)에게 임상연구 기간 중에 언제든지 추가적인 정보를 요청할 수 있습니다. 또한 귀하는 연구 연구대상자로서의 귀하의 권리에 대해 의문이 있을 경우 서울대학교병원 의학연구 윤리심의위원회(02-2072-0694)로 연락할 수 있습니다.

**연구대상자 동의서**

1. 본인은 임상연구에 대해 구두로 설명을 받고 상기 연구대상자 설명문을 읽었으며 담당 연구원과 이에 대하여 의논하였습니다.
2. 본인은 위험과 이득에 관하여 들었으며 나의 질문에 만족할 만한 답변을 얻었습니다.
3. 본인은 이 연구에 참여하는 것에 대하여 자발적으로 동의합니다.
4. 본인은 이후의 치료에 영향을 받지 않고 언제든지 연구의 참여를 거부하거나 연구의 참여를 중도에 철회할 수 있고 이러한 결정이 나에게 어떠한 해가 되지 않을 것이라는 것을 알고 있습니다.
5. 본인은 이 설명서 및 동의서에 서명함으로써 의학 연구 목적으로 나의 개인정보가 현행 법률과 규정이 허용하는 범위 내에서 연구자가 수집하고 처리하는데 동의합니다.
6. 본인은 이 동의서 사본을 받을 것을 알고 있습니다.

연구대상자 성명 서명 날짜 (년/월/일)

동의서 받은 연구원 성명 서명 날짜 (년/월/일)

연구책임자 성명 서명 날짜 (년/월/일)

(해당 되는 경우)

법적 대리인 성명 서명 날짜 (년/월/일)

입회인 성명 서명 날짜 (년/월/일)

**나. 미성년 연구대상자 설명서 및 동의서**

연구 과제명: 유전성 난청의 진단, 치료법 개발을 위한 기전의 연구

연구 책임자: 서울대학교병원 이비인후과 오승하 교수

이 설명서에는 이해되지 않는 말들이 포함되어 있을 수 있습니다.

이해가 분명하게 되지 않는 말이나 정보에 대해서는 연구를 담당하는 연구원 선생님 서울대학교 이비인후과 소속 이상연 교수(연구책임자, 2072-1478)에게 문의하시기 바랍니다.

1. 이 연구를 왜 하나요?

이 연구는 이상연 교수님과 연구원 선생님들이 유전성 난청을 가진 어린이들에 대해 알기 위해 많은 정보를 얻고자 이 연구를 실시하고 있습니다. 우리는 여러분에게 이 연구에 대해 설명한 후 여러분이 이 연구에 참여할지 물어볼 것입니다.

2. 왜 저에게 참여하라고 하시는 건가요?

전국에서 유전성 난청을 가진 어린이 또는 유전성 난청 여부를 알고 싶은 어린이가 이 연구에 참여할 것입니다. 연구원은 여러분이 그 어린이들 중 하나가 될 수 있다고 생각하여 참여하고 싶은 지를 묻는 것입니다.

3. 꼭 참여해야 하나요?

원하지 않으면 참여하지 않아도 되며 참여하지 않아도 여러분에게 해가 되는 일은 없습니다.

4. 연구 중에 어떤 일을 하나요?

연구원 선생님이 여러분에게서 피를 뽑아 갈 것입니다. 보호자에게도 여러분과 관련된 몇 가지 질문을 할 수 있습니다. 여러분과 보호자가 허락하면 이 연구에서 얻은 정보들을 연구하는 다른 선생님들과 공유하게 될 것입니다. 이 때 여러분의 이름은 알려지지 않을 것입니다.

만약 피에서 검사 결과가 나오지 않거나 추가적인 검사가 필요하다면, 추가적으로 앞쪽 팔이나 귀 뒤 부분의 피부 아주 조금을 채취할 것입니다. 마취 후에 피부 일부분을 채취할 것이므로 통증은 처음 마취 시에만 있으며, 이후엔 아프지 않을 것입니다. 피부는 실로 봉합을 하며, 실은 일주일 뒤에 병원에서 제거할 것입니다.

5. 이 연구가 저에게 어떠한 도움이 되나요?

이 연구는 여러분에게 직접적인 도움이 되지 않을 수도 있습니다. 그러나 이 연구가 나중에 여러분과 같은 어린이들에게 도움이 될 수 있습니다.

6. 궁금한 것이 있으면 어떻게 하나요?

연구에 대해 궁금한 것이 있거나 읽고 나서 이해가 안 가는 것은 무엇이든 연구원 선생님

이나 부모님 혹은 보호자에게 설명을 해 달라고 하십시오. 원한다면 “보호자용 설명서”를 읽어 볼 수도 있습니다.

7. 뽑은 피와 피부 조직은 어떻게 보관되고 언제 폐기되나요?

채혈한 피와 피부 조직은 인체유래물연구동의서에 기입한 기간 동안 서울대학교병원 이비인후과에서 보관할 것이고, 다른 연구 목적에 대한 제공 여부에 따라 이외 연구에 활용될 수 있고, 기입한 기한이 지나면 정해진 절차에 따라 폐기할 예정입니다. 제공한 혈액과 피부 조직을 이용해 유전분석을 하고 연구 결과가 나오면 이를 학회와 학술지에 발표할 예정이지만, 환자의 이름 등 개인정보는 노출되지 않습니다.

이 설명서는 여러분이 보관할 수 있도록 연구원 선생님이 복사해 줄 것입니다.

여러분이 이 연구에 참여하기 위해서는 부모님이나 법적 보호자도 별도의 동의서 양식에 서명해야 합니다.

아래 사항을 확인한 후 연구에 참여하길 원한다면 서명해 주십시오.

1. 나는 이 설명서를 읽었습니다.

2. 나의 모든 궁금한 점은 완전히 이해할 수 있도록 연구원에게서 설명 받았습니다.

3. 나는 이 연구에 참여 할 것을 동의합니다.

    연구대상자 아동 명                   서  명          날 짜 (년/월일)

  동의서 받은 연구원 명             서  명          날 짜 (년/월/일)

      연구 책임자 명                서  명           날 짜 (년/월/일)

**대리인 동의서**

1. 본인은 임상연구에 대해 구두로 설명을 받고 상기 연구대상자 설명문을 읽었으며 담당 연구원과 이에 대하여 의논하였습니다.

2. 본인은 연구로 인해 연구대상자에게 미치는 위험과 이득에 관하여 들었으며 나의 질문에 만족할 만한 답변을 얻었습니다.

3. 본인은 연구대상자(나의 자녀)가 연구에 참여하는 것에 대하여 연구대상자를 대신하여 동의합니다.

4. 본인은 연구대상자가 이후의 치료에 영향을 받지 않고 언제든지 연구의 참여를 거부하거나 연구의 참여를 중도에 철회할 수 있고 이러한 결정이 나에게 어떠한 해가 되지 않을 것이라는 것을 알고 있습니다.

5. 본인은 이 설명서 및 동의서에 서명함으로써 의학 연구 목적으로 연구대상자(나의 자녀)의 개인정보가 현행 법률과 규정이 허용하는 범위 내에서 연구자가 수집하고 처리하는데 동의합니다.

6. 본인은 이 동의서 사본을 받을 것을 알고 있습니다.

연구대상자 성명 서명 날짜 (년/월/일)

동의서 받은 연구원 성명 서명 날짜 (년/월/일)

연구책임자 성명 서명 날짜 (년/월/일)

(해당 되는 경우)

법적 대리인 성명 서명 날짜 (년/월/일)

입회인 성명 서명 날짜 (년/월/일)
