# Supplementary material for: Comprehensive genetic profiling of sensorineural hearing loss using an integrative diagnostic approach
Source: Cell Rep Med. 2025 Jun 30;6(7):102206. doi: 10.1016/j.xcrm.2025.102206 (PMC12281402; doi:10.1016/j.xcrm.2025.102206)
Supplement: Data S1. Informed consent form for patient with SNHL [file mmc5.zip › IRB Decision Notification Document (SNUH)(0905-041-281)_2.pdf]

SNUCM/SNUH IRB
Seoul National University College of Medicine
Seoul National University Hospital Institutional Review Board

Tel :82-2-2072-0694
FAX:82-2-3675-6824 101 Daehak-ro, Jongno-gu, Seoul, 03080, Korea

IRB SNUCM SNUH 의학연구윤리심의위원회

IRB Decision Notification Document

IRBNO : H-0905-041-281

RESEARCH TITLE : Development of diagnostic and therapeutic modalities in hereditary hearing loss

INVESTIGATOR : SANG YEON LEE

SPONSOR :

REVIEW LIST
1. Continuing Review
2. Protocol Amendment

REVIEW DATE: 2024-10-18

REVIEW RESULT AND COMMENTS
- According to [the Study Progression Checklist], the research progression was reviewed.
- According to [the IRB Approval Criteria], the IRB approves the research.

IRB APPROVAL OF PROTOCOL WILL EXPIRE ON 2025-10-20.

ALL CONDITIONS OF APPROVAL PREVIOUSLY ESTABLISHED BY SNUCM/SNUH IRB
FOR THIS RESEARCH PROJECT CONTINUE TO APPLY.
IF YOU HAVE ANY QUESTIONS, CONTACT SNUCM/SNUH IRB (Tel: 82-2-2072-0694)

Institutional Review Board Chairman 의학연구윤리심의위원회위원장인

All investigators performing SNUH IRB approved projects must comply with the followings:

1. Enrollment of participated subjects before the IRB approval of protocol/protocol amendment is forbidden.

2. To conduct the study according to the approved protocol. To conduct the study differently from the original protocol is forbidden.

3. To use the approved Informed Consent Form.

4. The informed consent process shall be conducted based on sufficient explanation under no coercion or unfair influence, and a potential subject shall be provided with sufficient opportunity to consider the study participation.

5. Except for the unavoidable cases to protect subjects during the study conduct, any amendment of the study shall be implemented after getting the prior approval of the Board, and any amendment taken in an emergency situation for protection of subjects shall be immediately reported to the Board.

6. In case the study should be conducted differently from the original protocol since the immediate risk factor occurring to subjects should be eliminated, the amendment item that may increase risk factors occurring to subjects or have serious effects of the study conduct, items on the unexpected serious adverse drug reaction, or items on new information that may have negative effects on subjects' safety or study conduct shall be promptly reported to the Board.

7. The subject recruitment advertisement approved by the Board shall be used.

8. To submit an annual continuation report before the expiration date of the IRB approval. This is not required for the exempt research.

9. In the case of a decision by the Board to disapprove, you may have the opportunity to submit an appeal in writing within 1 month since when the IRB decision is notified. You can appeal only once for the same item.

10. In the case of a decision by the Board to disapprove, you may have the opportunity to submit an appeal in writing. However, you should not file an appeal 2 times in a row with the same reason.

11. When completing the research, Study completion report and Study result report shall be submitted.

12. You shall comply with Bioethics and Safety Act, Pharmaceutical Affairs Act/Medical Device Affairs Act as defined under the Ministry of Food and Drug Safety (MFDS) regulations, the International Conference on Harmonization (ICH) guidelines and the Declaration of Helsinki.

13. According to the Declaration of Helsinki, all clinical studies shall be disclosed in the database that allows public access(primary registry) prior to the first subject enrollment; for example, you may use http://register.clinicaltrials.gov. For details, please refer to the IRB website.

14. The internal audit or inspection from the regulatory agency for the approved study could be conducted. Investigator shall cooperate in helping this to carry out, when requested for reading of study document (including electronic document) by internal auditor or monitor from sponsor, or inspector from regulatory agency.

This is to certify that the information contained herein is true and correct as reflected in the records of the SNUCM/SNUH IRB. **We certify that SNUCM/SNUH IRB is in full compliance with Good Clinical Practice as defined under the Korean Ministry of Food and Drug Safety (MFDS) regulations and the International Conference on Harmonisation (ICH) guidelines.**
